# Non-lytic viral immunotherapy induces long-term glioblastoma survival and tumor-specific immunity without eliciting an antiviral response

**DOI:** 10.1038/s41467-026-72746-5

**Published:** 2026-05-19

**Authors:** Alexander F. Haddad, Atul Saha, Sara A. Collins, Isabella Lovalvo, Sabraj Gill, Megan L. Montoya, Poojan Shukla, Jinpyo Hong, Elaina Wang, Pavlina Chuntova, Meeki Lad, Robert Osorio, Jia-Shu Chen, Melissa Sathavipat, Saket Jain, Eric Chalif, Noriyuki Kasahara, Manish K. Aghi

**Affiliations:** 1 Department of Neurological Surgery, University of California, San Francisco, San Francisco, CA USA; 2 Department of Radiation Oncology, University of California, San Francisco, San Francisco, CA USA; 3 Present Address: School of Medicine, University of California, San Diego, La Jolla, CA USA; 4 Present Address: 4D Molecular Therapeutics, Emeryville, CA USA; 5 Present Address: Genentech, South San Francisco, CA USA; 6 Present Address: Penn State College of Medicine, Hershey, PA USA; 7 Present Address: Department of Neurosurgery, Brown University, Providence, RI USA; 8 Present Address: Department of Neurosurgery, Brigham and Women’s Hospital, Harvard Medical School, Boston, MA USA

**Keywords:** CNS cancer, Cancer immunotherapy, Tumour immunology, Gene therapy

## Abstract

Glioblastoma is a lethal brain tumor that is resistant to conventional therapies. Here we present a non-lytic replicating retrovirus (RRV) that delivers an IL-15-receptor-linked fusion protein (RLI) superagonist directly into glioblastoma cells, creating localized immunotherapy biofactories. In orthotopic mouse models, RRV-RLI dramatically suppresses tumor growth, prolongs survival, and induces lasting remission with immunologic memory. Mechanistically, we observe increased CD8⁺ T cell and natural killer cell infiltration and activation, alongside elevated antigen presentation pathways. Combining RRV-RLI with temozolomide, which is standard-of-care chemotherapy for glioblastoma, enhances antitumor immunity. T cell receptor sequencing reveals a polyclonal repertoire of T cells, enhanced by combining RRV-RLI with temozolomide. Analysis of the T-cell repertoire suggests it to be directed against tumor rather than viral antigens, supporting the specificity and re-applicability of our approach. These findings illustrate that RRV-RLI reprograms glioblastoma into an immunostimulatory hub, offering a viral immunotherapy against glioblastoma and potentially other therapy-resistant solid tumors.

## Abstract (web summary)

Glioblastomas represent a malignant brain tumour with high mortality rate due to resistance to traditional therapies. Here the authors propose a strategy of delivering an IL-15 superagonist IL-15-receptor-linked fusion protein by a non-lytic replicating retrovirus directly to the tumours, which shows high therapeutic efficacy in a mouse model.

## Introduction

Glioblastoma (GBM) is a devastating brain cancer and carries a poor prognosis despite standard-of-care treatment, including surgical resection, radiation, and chemotherapy^1^. The median survival for newly diagnosed GBM is 15 months, with only modest improvements over the past decade and a need for additional therapeutics^2–5^. Despite success in other cancer types, systemically delivered single-agent immunotherapy has thus far seen limited success in treating GBM^4^, due to the tumor’s uniquely immunosuppressive microenvironment^3,4,6–9^. Several characteristics contribute to the immunodeficient and immunosuppressive tumor microenvironment (TME) of GBM, including the downregulation of MHC I^7–9^, low tumor mutational burden (TMB)^10^, tumor intrinsic signaling pathways^11^, and infiltration of immunosuppressive myeloid cells^12^. A key contributor to the immunosuppression seen in GBM is the low number of tumor-infiltrating T cells, which are often exhausted and dysfunctional^6,13,14^. GBM patients also have low systemic T cell counts due to their sequestration in the bone marrow, further compounding the reduced antitumor T cell activity seen^15^.

Systemic immunotherapy for GBM faces additional challenges. The doses needed to overcome the severe immunosuppression and cross the blood-brain barrier may be high enough to cause systemic toxicities such as autoimmune side effects or cytokine storm^15–17^. Consequently, there is a growing interest in local immunotherapies that deliver immunostimulatory proteins like cytokines directly to tumor cells^18,19^. While local immunotherapies investigated to date have taken many forms, such as nanoparticles, scaffolds, and hydrogels, one of the most promising is the use of viral vectors to deliver therapeutic transgenes, such as a suicide gene or local immunotherapy directly to the tumor microenvironment^18,19^.

Replicating retrovirus (RRV) is a promising viral vector for cancer gene therapy that selectively infects, stably integrates, and replicates in tumor cells^20^. Unlike other viral vectors, RRV is relatively immunologically silent, allowing widespread tumor dissemination with minimal immune clearance^21^. RRV delivering the suicide gene yeast cytosine deaminase (CD) has been used to treat recurrent GBM in clinical trials, revealing an excellent safety profile^22,23^. Molecular profiling of treated patients confirmed the tumor selectivity and safety of RRV^22^, with virus only transiently detected in patient blood with quantitative signal in 6% of plasma samples and 3% of whole blood samples. While integration sites were identified in blood and tumor samples, there was no evidence to suggest clonal expansion associated with integration and no patient in an RRV trial has developed lymphoma to date^22^. Unfortunately, in a randomized phase III trial, RRV carrying CD did not meet survival endpoints overall, although there was some benefit in pre-specified patient subgroups, and further clinical evaluation is ongoing. Preclinical studies of RRV-CD implicated activation of antitumor immunity as critical for long-term survival after treatment, indicating that an immunostimulatory payload may enable improved therapeutic efficacy^24^.

Immunostimulatory cytokines, such as interleukin-2 (IL-2) and interleukin-15 (IL-15), represent promising candidates for transgene therapy due to their capacity to enhance the proliferation of Natural Killer (NK) and T cells^25^. Of particular interest is IL-15, which exhibits a favorable profile by stimulating the expansion of NK- and T cells while also promoting their cytotoxic function and release of additional inflammatory cytokines^25^. Unlike IL-2, IL-15 does not stimulate T regulatory (Treg) cells which can suppress antitumor immunity, thereby offering a therapeutic advantage^25^. The clinical utility of IL-15 alone is in part limited by its complex signaling pathway, involving trans-presentation from a presenting cell through IL-15/IL-15Rα complexes to NK- and T cells, where they can stimulate activation via binding to the β/γC receptor complex. In oncology patients, the expression of IL-15Rα is frequently downregulated, and IL-15 has a relatively short half-life, further complicating its application as a therapeutic agent.

These limitations have inspired the development of superagonists which can directly bind to effector cells and have increased stability^26–28^. One such superagonist is receptor-linked–IL-15 (RLI), which is bound to the sushi domain of the IL-15Rα, allowing it to bind directly to IL-15 receptors on NK and T cells and improving stability. Systemically administered RLI has been shown to slow tumor growth and promote antitumor immune activity in multiple preclinical cancer models^26–28^.

Given the promising preclinical results associated with systemic RLI treatment, we hypothesize that local RLI therapy delivered via RRV will stimulate an antitumor immune response without systemic side effects. In the present study, we engineer an RRV expressing RLI and demonstrate its ability to increase survival in poorly immunogenic mouse models of GBM in a T cell-dependent manner. RRV-RLI also functions in the setting of GBM standard-of-care chemotherapy with leukocyte single-cell sequencing revealing clonal T cell expansion, enhanced antigen-driven immune response, and favorable shifts in myeloid populations when the two treatments were combined. T-cell receptor sequencing revealed a selective expansion of tumor antigen-specific receptors, with no detectable evidence of an antiviral immune response. This approach provides a viral cancer immunotherapy for GBM with a potential for clinical translation.

## Results

### IL15 expression is associated with immune signaling and T cell Infiltration in glioblastoma, with implications for gene therapy

**Fig. 1:**
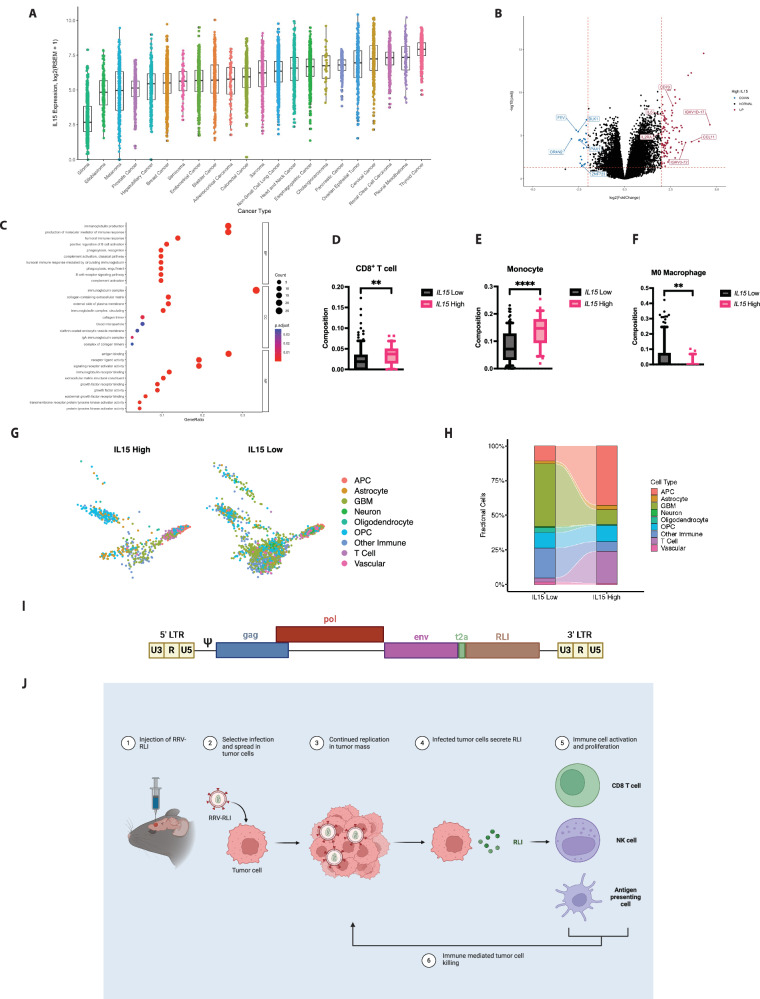
Human *IL15* expression in various cancers and its biologic effects in GBM informs the decision to create RRV-RLI. **A** *IL15* expression across cancer types (box plots show median and 1.5xIQR). **B** Volcano plot demonstrating differential gene expression between IL15 high and low glioblastoma tumors. **C** Gene ontology analysis comparing *IL15* high and low glioblastoma tumors. **D** Calculated CD8^+^ T cell infiltration based on bulk RNA sequencing with increased infiltration in *IL15* high samples (*n* = 120, *IL15* low, *n* = 31 *IL15* high, p = 0.007, Mann-Whitney U, two-tailed uncorrected). **E** Calculated monocyte infiltration based on bulk RNA sequencing demonstrating increased infiltration in *IL15* high tumors (*n* = 120, *IL15* low, *n* = 31 *IL15* high, *p* < 0.0001, Mann-Whitney U, two-tailed uncorrected). **F** Calculated M0 macrophage infiltration based on bulk RNA sequencing showing decreased infiltration in *IL15* high tumors (*n* = 120, *IL15* low, *n* = 31 *IL15* high, *p* = 0.009, Mann-Whitney U, two-tailed uncorrected). **D–F** Box plots show median, 25^th^–75^th^ percentiles (box), and 10^th^–90^th^ percentiles (whiskers). **G** Single-cell RNA sequencing UMAP of human glioblastoma tumors comparing *IL15* high vs. low tumors. **H** Alluvial plot demonstrating changes in populations between human *IL15* high and low samples with increased T cell and APC infiltration in an *IL15* high state (APC antigen-presenting cell, identified by SingleR annotation and expression of CIITA, CD74, and CD86). **I** Schematic of the RRV-RLI construct with RLI placed after the RRV env protein. **J** Proposed mechanism of RRV-RLI treatment. *p < 0.05; **p < 0.01; ***p < 0.001; ****p < 0.0001. Data represent biological replicates (individual patient datapoints). Source data available per data availability statement. Figure 1**I** (https://BioRender.com/qxms6jp) and **J** (https://BioRender.com/7k8j27s) created in BioRender. Lab, A. (2026).

To better understand the applicability of IL-15 as a cancer immunotherapy, we examined baseline *IL15* expression across various cancer types and investigated differences between *IL15* high and low glioblastoma (GBM) tumors. Analysis of bulk RNA sequencing data from the TCGA Pan Cancer Atlas revealed that gliomas of all grades, including GBM, exhibited among the lowest levels of *IL15* expression across cancer types, whereas thyroid cancer showed the highest expression (Fig. 1A). In GBM tumors with high *IL15* expression, we observed significantly increased expression of immune and inflammatory genes such as *IL2RA, CD70, IL6, and CCL11*, as well as immunoglobulin genes *IGKV1D-17* and *IGHV3-72* (Fig. 1B). Subsequent gene ontology analysis indicated upregulation of pathways associated with immunoglobulin production, phagocytosis, antigen binding, receptor-ligand activity, and molecular mediators of immune response (Fig. 1C).

CIBERSORT analysis of 22 different immune cell populations revealed a significant increase in infiltrating CD8⁺ T cells and monocytes in the *IL15* high tumors, along with a decrease in infiltrating M0 macrophages (Fig. 1D–F). Analysis of available single-cell RNA sequencing data from four resected GBM tumors supported these findings, demonstrating increased infiltration of T cells and antigen-presenting cells (APCs) (Fig. 1G, H)^29^. Because checkpoint molecule expression (including PDCD1/PD-1, CTLA4, LAG3, and TIGIT) was captured in only a small fraction of infiltrating immune cells, the dataset lacked sufficient depth to support a reliable comparison of immune-checkpoint expression between IL-15 high and low tumors. The exception was HAVCR2 (TIM3), which was present in 57% of lymphoid cells with no difference between the high and low IL-15 tumors (*p* = 0.66, Mann–Whitney U test). Together, these data identified GBM as an *IL15*-deficient tumor with a more favorable antitumoral immune microenvironment in those GBMs that happened to have high *IL15* expression, implicating IL-15 as an intriguing gene therapy strategy in GBM.

We therefore generated RRV-RLI, an RRV expressing a codon- and stability-optimized RLI insert following a T2A cleavage site on the RRV backbone as previously described (Fig. 1I)^20^. RRVs replicate well in tumors due to their reliance on two hallmarks of cancer, sustained proliferative signaling and immunosuppression in the tumor microenvironment (Supplementary Fig. 1A)^24^. As such, we aimed to utilize RRV-RLI to convert tumor cells into biofactories for secreted RLI in order to enhance the activation and proliferation of CD8^+^ T cells, NK cells, and antigen-presenting cells and generate an antitumor immune response (Fig. 1J).

**Fig. 2:**
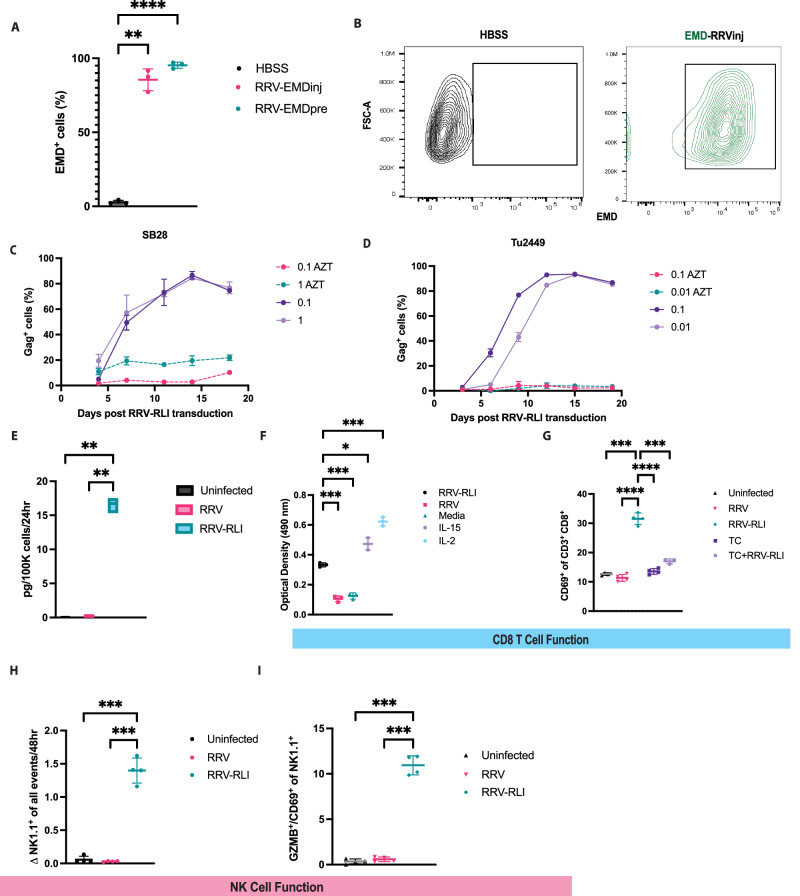
Validation of the biologic effects of RRV-RLI in cultured glioblastoma cells. **A** In vivo spread of RRV-EMD in the SB28 GBM murine tumor model at endpoint. RRV-EMDinj (*p* = 0.002) and RRV-EMDpre (*p* < 0.0001, *n* = 3 per group) demonstrated significantly higher infection rates compared to HBSS injection control without a difference between delivery modalities. **B** Example gating of RRV-EMD in vivo spread. **C** In vitro RRV-RLI replication in SB28 is efficient, reaching > 80% tumor cell infection by day 15 regardless of starting multiplicity of infection (MOI) as measured by the expression of viral Gag protein on flow cytometry with no change in AZT control groups (*n* = 3 per group). **D** In vitro RRV-RLI replication in Tu2449 is similarly effective, reaching > 90% tumor cell infection by day 15 regardless of starting multiplicity of infection (MOI) as measured by the expression of viral Gag protein on flow cytometry with no change in AZT control groups (*n* = 3 per group). **E** RRV-RLI stimulates production of 16.7 pg/100 K cells over 24 h in the SB28 cell line with minimal production in RRV (no transgene, 0.3 pg/100 K cells/24 h, *p* = 0.001) or uninfected control cells (0.02 pg/100 K cells/24 h, *p* = 0.002, *n* = 3 per group). **F** RLI produced by SB28 cells is functional and stimulates the growth of cytokine dependent CTLL-2 cells as compared to RRV (*p* = 0.0002) and media control (*p* = 0.0003). Nanomolar matched (0.361 nM) recombinant IL-15 (*p* = 0.02) and IL-2 (*p* = 0.0008, Welch’s t-test) demonstrated appropriate functional activity (*n* = 3 per group). **G** Co-culture of CD8 T cells with infected SB28 tumor cells demonstrates increased CD69 after 48 hours compared to RRV and uninfected cells (*p* < 0.0001 and *p* = 0.0002, *n* = 3 for TC + RRV-RLI, *n* = 4 for other groups). **H** Co-culture of NK cells with infected SB28 tumor cells demonstrates increased change in NK cell frequency relative to all events at 48 h when compared to RRV infected and uninfected (mean: 1.4 vs. 0.06, vs. 0.03, *p* = 0.0007 and *p* = 0.0004, *n* = 4 per group). **I** In NK cell: SB28 tumor cell co-culture, there was increased double positivity for GZMB and CD69 at 24 h in the RRV-RLI condition vs. RRV and uninfected (*p* = 0.0002 and *p* = 0.0001, *n* = 4 per group). *p < 0.05; **p < 0.01; ***p < 0.001; ****p < 0.0001. TC = T cell. Figure 2E shows floating bars from minimum to maximum values and a line at mean, otherwise graphs show mean +/− SD. All statistical tests in Fig. 2 represent uncorrected two-sided Welch’s t-test. Data represent biological replicates. Source data are provided as a Source Data file.

RRV-RLI efficiently infects and spreads in murine GBM, driving the secretion of functional RLI. We first sought to understand viral infectivity kinetics in mouse orthotopic GBM tumors in vivo through direct intratumoral injection. We confirmed that SB28 murine GBMs can support the replication of an RRV expressing the emerald (EMD) protein in vivo, with on average 85.5% of intracranial SB28 GBM cells expressing the EMD protein at tumor endpoint after direct intracranial injection, similar to what was seen after implanting a 4% virus positive ex vivo-transduced tumor (Fig. 2A, example gating Fig. 2B). This also confirmed the efficacy of intracranial injection for viral delivery. We then sought to assess replication and stability of the RRV-RLI viral construct in cultured murine GBM tumor cell lines. Figure 2C demonstrates the ability of RRV-RLI to efficiently spread through cultured SB28 cells beginning with multiplicities of infection (MOIs) of 0.1 and 1. Transduced cells exposed to azidothymidine (AZT), which inhibits viral spread, showed no increase in RRV-RLI over time, providing further assurance about the safety of this approach (Fig. 2C). Similar kinetics of RRV-RLI spread were noted in cultured Tu-2449 murine GBM cells (Fig. 2D). RRV-RLI was also noted to spread efficiently in GBM43 and G55, two human patient-derived cell lines (Supplementary Fig. 1B). Genetic stability of RRV-RLI over serial infection cycles, without transgene deletion during virus replication, was confirmed by polymerase chain reaction (PCR) using primers flanking the transgene insert site (Supplementary Fig. 1C).

Having confirmed viral kinetics and stability, we then aimed to investigate the production and function of RLI produced by infected tumor biofactories. Using an IL-15 ELISA, we found that SB28 cells infected with RRV-RLI secreted RLI at an average rate of 16.7 pg/100 K cells/24 hours (Fig. 2E). To assess whether the IL-15 secreted by RRV-RLI-transduced murine GBM cells retained its canonical functions, we performed a series of functional assays. First, conditioned media (CM) from RRV-RLI-infected SB28 cells was applied to CTLL-2 cells, a cytokine-dependent cytotoxic T cell line derived from C57BL/6 mice^30^. The RLI secreted from infected tumor cells was able to stimulate CTLL-2 growth to a level comparable to a recombinant IL-15 control given at the same concentration (0.33 OD vs. 0.47 OD, *p* = 0.01, Fig. 2F). Additionally, in a co-culture system with SB28 tumor cells and naïve isolated mouse CD8^+^ T cells, RRV-RLI infection of the SB28 cells significantly promoted the expression of the canonical T cell activation marker CD69 at a higher level than T cells cocultured with SB28 cells infected with RRV alone or uninfected SB28 tumor cells (*p* < 0.005, Fig. 2G); confirming the functional ability of RLI to activate T cells. We also observed increased CD3^+^CD8^+^ T  cell proliferation as indicated by Ki67 staining at 72 h of incubation (*p* < 0.05, Supplementary Fig. 1D) and improved CD8^+^ T cell persistence in culture (*p* < 0.05, Supplementary Fig. 1E**)**. Subsequent co-culture assays with RRV-RLI-transduced SB28 cells and murine NK cells revealed the ability of RLI to significantly drive NK-cell proliferation (*p* < 0.005, Fig. 2H). These co-culture assays also revealed the ability of RLI secreted by RRV-RLI-transduced SB28 cells to promote NK-cell activation, as evidenced by increased frequencies of double-positive CD69^+^GZMB^+^ NK-cells (*p* < 0.005, Fig. 2I) as well as CD69^+^ and GZMB^+^ single-positive NK-cells (Supplementary Fig. 1F and G, gating strategy Supplementary Fig. 1H). Additional analysis of our T cell co-culture system demonstrated a significant change in PD1^+^ T cells in the RRV-RLI treatment group relative to RRV-RLI added to T cells directly (*p* < 0.005, Supplementary Fig. 1I) without significant evidence of double positive PD1/TIM3 and PD1/LAG3 populations or CTLA4 expression, consistent with RLI activating T cells as PD-1 expression in the absence of other exhaustion markers with concurrent CD69 co-expression is a sign of T cell activation rather than exhaustion^31^. RRV-RLI virus added to T cells directly did not significantly impact activation or exhaustion marker expression (Fig. 2G, Supplementary Fig. 1I).

### RRV-RLI treatment decreases intracranial tumor growth and prolongs survival in two poorly immunogenic murine models of glioblastoma

**Fig. 3:**
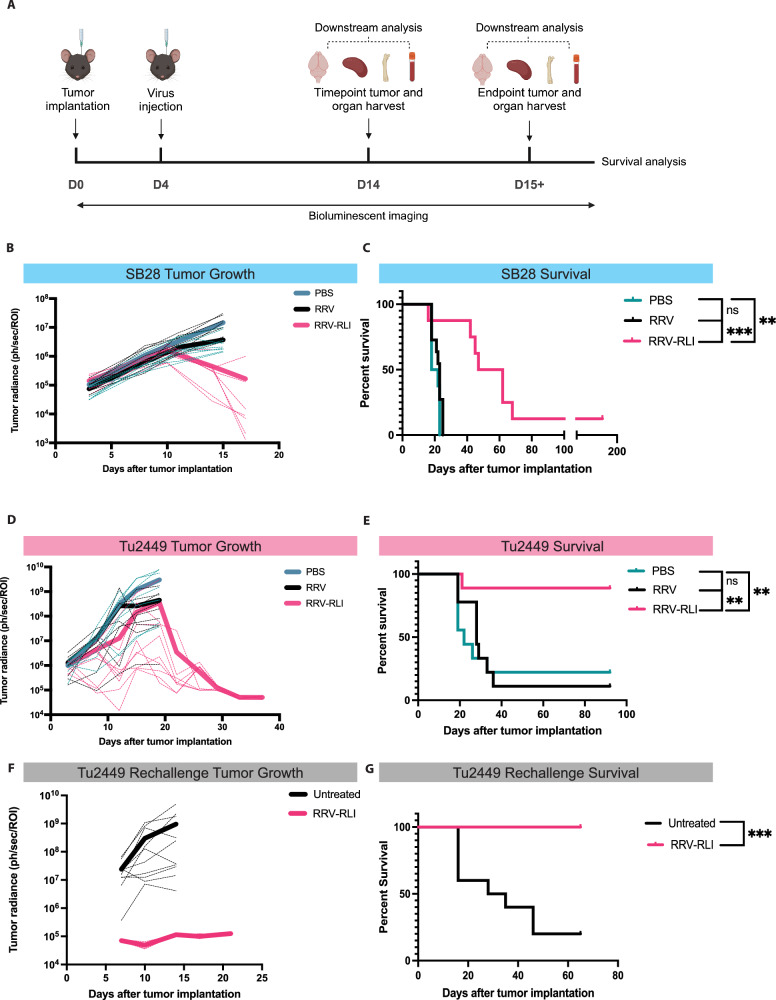
RRV-RLI therapy suppresses tumor growth and prolongs survival in two poorly immunogenic murine glioblastoma models. **A** Schematic illustration of RRV-RLI in vivo assessments. **B** RRV-RLI treatment reduces average bioluminescent signaling 10 days post-tumor implantation in SB28 tumors. **C** RRV-RLI significantly extends survival relative to PBS treated (median survival 54.5 days vs. 20 days, *p* = 0.002, Log-Rank Mantel-Cox) and RRV treated (median survival 54.5 days vs. 23 days, *p* = 0.0008, Log-Rank Mantel-Cox) mice with SB28 tumors (*n* = 11 RRV group, *n* = 8 PBS group, *n* = 8 RRV-RLI group). **D** RRV-RLI treatment decreases average bioluminescent signaling beginning 10 days after tumor implantation in Tu2449 tumors. **E** RRV-RLI significantly prolongs survival relative to PBS treated (median survival undefined vs. 22 days, *p* = 0.005, Log-Rank Mantel-Cox) and RRV treated (median survival undefined vs. 28 days, *p* = 0.002, Log-Rank Mantel-Cox) mice with Tu2449 tumors (*n* = 9 RRV group, *n* = 9 PBS group, *n* = 9 RRV-RLI group). **F** No evidence of tumor growth is seen in bioluminescent imaging after contralateral intracerebral injection of 10,000 Tu2449 tumor cells in previously cured long-term survivors. **G** Rechallenged mice display long-term survivorship relative to control (non-previously treated) mice (median survival undefined vs. 28 days, *p* = 0.001, Log-Rank Mantel-Cox) (*n* = 10 untreated control group, *n* = 8 RRV-RLI long term survivor group). *p < 0.05; **p < 0.01; ***p < 0.001; ****p < 0.0001. Data represent biological replicates. Source data are provided as a Source Data file. (**A**) created in BioRender. Lab, A. (2026) https://BioRender.com/nl1jrcq.

Having validated the stability and immunostimulatory effects of RRV-RLI in culture, we next evaluated its therapeutic potential in vivo using SB28 and Tu2449 murine GBM lines implanted intracranially in syngeneic C57BL/6 and B6C3F1 mice (Fig. 3A). In the SB28 model, RRV-RLI treatment significantly reduced tumor growth (Fig. 3B) and extended median survival compared to control PBS (20 days, *p* = 0.002 vs. RRV-RLI) or RRV alone (23 days *p* = 0.0008 vs. RRV-RLI) vs. 54.5 days (RRV-RLI)), with long-term survival in 12% of treated mice (Fig. 3C). These findings were recapitulated and improved in the Tu2449 murine GBM model. In Tu2449 tumors, RRV-RLI treatment led to pronounced tumor growth reduction on bioluminescent imaging (BLI) (Fig. 3D). Survival of RRV-RLI-treated mice with Tu2449 GBMs was similarly significantly increased relative to control mice, with the majority of RRV-RLI-treated mice experiencing complete tumor regression and long-term survival (*p* = 0.005, Fig. 3E). Rechallenge of mice cured from Tu2449 GBMs with contralateral intracranial injections of Tu2449 tumor cells demonstrated immunologic memory and rejection of injected cells with sustained survival relative to untreated age-matched tumor-bearing controls (Fig. 3F, G). Quantitative PCR of tumors sampled 10 days after virus injection detected the presence of RRV-RLI viral copies in tumor DNA (13.3 vs. 0 viral copies/ngDNA, *p* < 0.01, Supplementary Fig. 2A). Subsequent ELISA of tumor samples similarly demonstrated high intratumoral RLI protein expression in RRV-RLI-treated tumors compared to tumors receiving intratumoral PBS (381 vs. 0 pg/mL, Supplementary Fig. 2B). Blood samples from RRV-RLI-treated SB28 mice showed no detectable RLI, confirming the localized effect of the treatment within the tumor immune microenvironment and the absence of systemic spread (Supplementary Fig. 2C). Further demonstration of the safety and tumor-specificity of intratumoral RRV-RLI was revealed when contralateral brain in RRV-RLI treated mice demonstrated no evidence of significant viral replication or spread via qPCR for viral DNA at endpoint relative to tumor in the same mouse (259 vs. 0.57 viral copies/ng DNA, *p* < 0.005, Supplementary Fig. 2D).

### RRV-RLI induces significant antitumor modulation of the GBM immune microenvironment

**Fig. 4:**
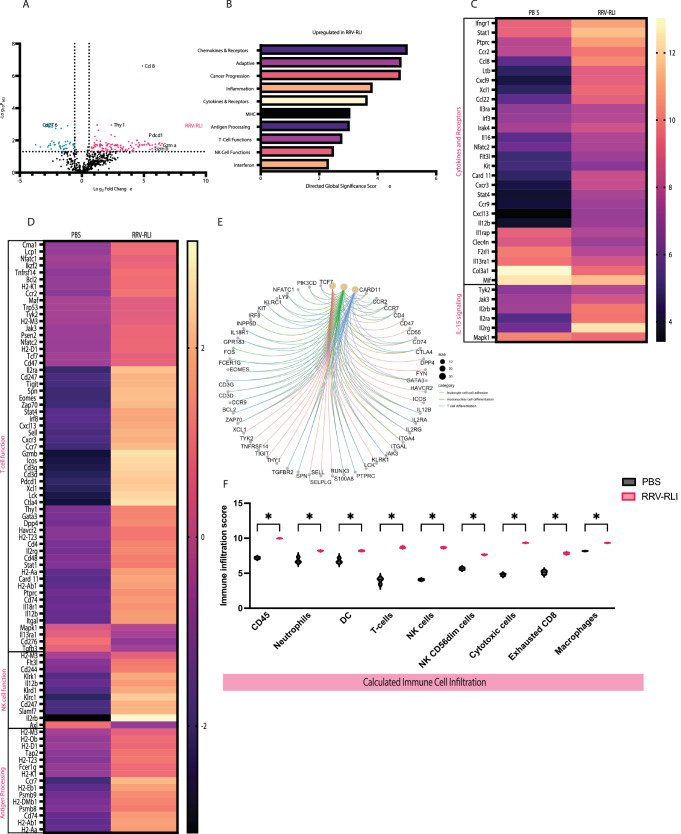
Comprehensive bulk transcriptomic analysis reveals extensive antitumor immune modulation induced by therapy. **A** Volcano plot showing differentially expressed genes between RRV-RLI and PBS treatment groups at day 14 post-tumor implantation timepoint (DESeq2, Benjamini-Hochberg adjusted *p* < 0.05 and log2FC > 0.5). **B** Directed global significance scores of the top 10 upregulated gene pathways in RRV-RLI treatment vs. PBS treatment. **C** Heat map detailing differential gene expression for genes related to cytokines and receptors and IL-15 signaling. **D** Heat map detailing differential gene expression for genes related to T cell function, NK cell function, and antigen processing between RRV-RLI and PBS treatment groups. **E** Gene network analysis of upregulated genes in RRV-RLI treated glioblastomas compared to PBS-treated controls reveals a highly interconnected immune response network. **F** Violin plot demonstrating calculated immune infiltration scores between RRV-RLI and PBS treatment groups at day 14 post-tumor implantation timepoint with significantly increased infiltration in RRV-RLI across cell type (uncorrected two-sided Welch’s t-test, macrophage: *p* < 0.0001, NK cells: *p* < 0.0001, cytotoxic cells: *p* = 0.0002, CD45: *p* = 0.0005, NK CD56dim cells *p* = 0.0009, exhausted CD8: *p* = 0.001, T cells: *p* = 0.002, DC: *p* = 0.02, Neutrophils: *p* = 0.03). *p < 0.05; **p < 0.01; ***p < 0.001; ****p < 0.0001. Data represent biological replicates, *n* = 3 for all groups shown. Source data are provided as a Source Data file.

To better understand the mechanisms behind RRV-RLI’s therapeutic benefit, we transcriptomically analyzed the effect of RRV-RLI treatment on immune cells in the SB28 microenvironment using the NanoString nCounter platform and a multiplex panel of 770 genes encoding markers of different immune cell types, common checkpoint inhibitors, and mediators of both the adaptive and innate immune responses. Differential gene expression analysis comparing RRV-RLI-treated tumors to PBS control tumors revealed that 10 days after RRV-RLI treatment, tumors exhibited elevated transcription of the T cell modulating and co-stimulatory protein *Thy1* (fold change = 5.2, padj = 0.001); chemokine *Ccl8* (fold change = 29, padj < 0.001); cytotoxic T-lymphocyte granule granzymes *Gzma* (fold change = 81, padj < 0.05) and *Gzmb* (fold change = 51, padj < 0.05); and T cell activation marker *Pdcd1* (fold change=42, padj < 0.05), as well as reduced expression of *Cd276* (fold change = −4.3, padj < 0.01), which encodes the immune checkpoint protein B7-H3 (associated with inhibiting cytotoxic T cell function and suppressing antitumor immune responses)^32,33^ (Fig. 4A). Gene set enrichment analysis (GSEA) comparing RRV-RLI to PBS treatment revealed that RRV-RLI promoted the upregulation of T cell and NK-cell functionality, antigen processing, and MHC pathways (Fig. 4B). Further interrogation of genes related to IL-15 mediated functions, including T cell and NK-cell functionality, antigen processing, and cytokine signaling revealed a diffuse upregulation in RRV-RLI-treated mice, including genes involved in the IL-15 signaling pathway (Fig. 4C, D). Significantly upregulated genes of specific relevance to IL-15 functions included those involved in MHC class I function and pathways (*H2-m3*, *H2-d1*, *Tap2*, *H2-t23*, *H2-k1*, *Psmb8*) and lymphocyte trafficking (*Ccr2, Ccr7, Ccr9, Cxcr3*) (Fig. 4C, D). Gene network analysis uncovered a highly interconnected immune response with extensive interactions among genes involved in leukocyte cell-cell adhesion, mononuclear cell differentiation, and T cell differentiation, supporting the notion of a coordinated immune response elicited by RRV-RLI treatment (Fig. 4E). Overall, RRV-RLI-treated SB28 GBMs demonstrated a transcriptomic profile reflecting increased tumor-infiltrating T cells, NK-cells, and dendritic cells relative to SB28 GBMs treated with PBS (Fig. 4F). In addition, the ratio of exhausted CD8^+^ to cytotoxic T cells, based on immune infiltration scores, was lower in the RRV-RLI treatment group (1.1 vs. 0.84, *p* = 0.04, uncorrected two-sided Welch’s t-test). These findings suggest that RRV-RLI treatment promotes a robust and coordinated antitumor immune response.

When comparing RRV-RLI to RRV to isolate the effect of RLI, we similarly observed increases in pathways related to NK-cell function, interferon, adhesion, T cell function, dendritic cell function, antigen processing, and MHC proteins (Supplementary Fig. 2E). Differential gene expression between RRV-RLI and RRV-treated GBMs revealed increases in *Klra6* (modulator of NK cell function, promotes killing in the setting of downregulated MHC I, fold change = 48, padj < 0.001) and *Tnfrsf9* (4-1BB, powerful co-stimulatory molecule critical for enhancing T cell immune responses, fold change = 160, padj < 0.001)^34^ (Supplementary Fig. 2F). Upregulation of genes involved in mediating T- and NK-cell function or lymphocyte trafficking was also seen (e.g., *Ccr2, Cxcr3, Cxcl12*) (Supplementary Fig. 2G). When comparing RRV-RLI-treated GBMs harvested at day 14 post-implantation versus at endpoint, we observed a significant decline in most infiltrating immune cell populations, including T cells and NK-cells. This suggests that the initial robust antitumor immune response induced by RRV-RLI treatment lacks durability in those mice that eventually failed to respond (Supplementary Fig. 2H). Additionally, mice treated with RRV alone showed a paucity of immune infiltration at the day 14 timepoint, implicating RLI as the primary driver of the therapeutic effect observed (Supplementary Fig. 2H).

### CD8^+^ and CD4^+^ T cells drive the antitumoral effects of RRV-RLI in murine GBM

**Fig. 5:**
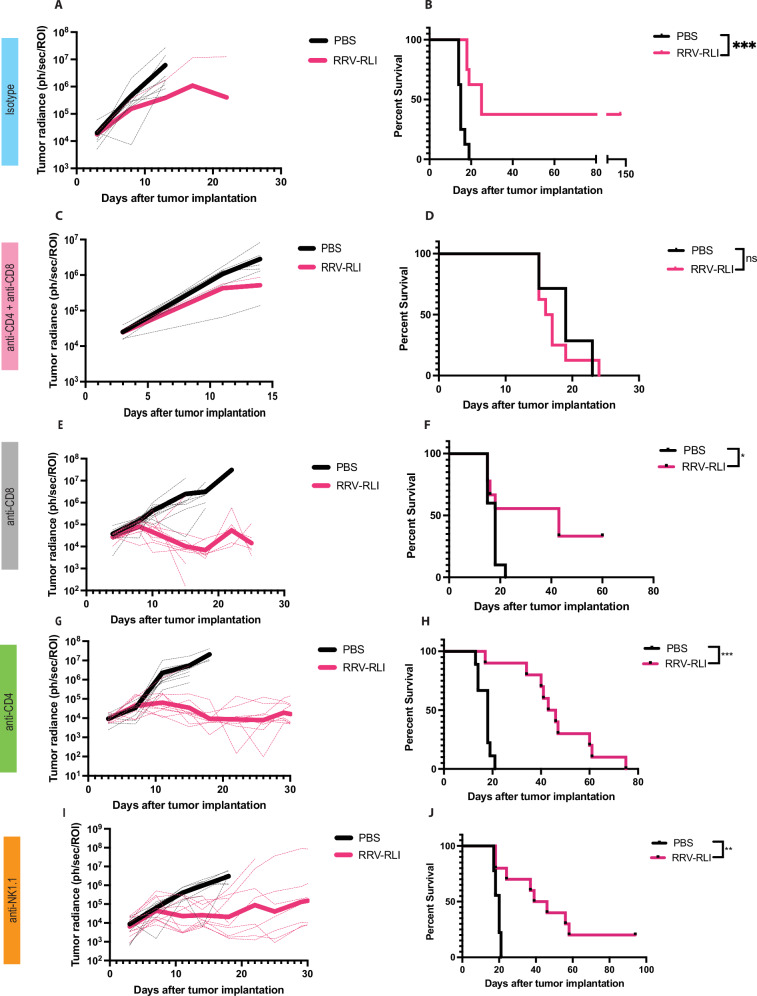
The survival benefit of RRV-RLI therapy depends on both CD4⁺ and CD8⁺ T Cells. **A** Bioluminescent imaging in SB28 tumors with RRV-RLI and isotype treatment. **B** In isotype antibody-treated mice bearing SB28 tumors, RRV-RLI therapy significantly extended survival compared to PBS-treated controls (median survival 25 days vs. 15 days; *p* = 0.0005, 37% long-term survivorship, Log-Rank Mantel-Cox test, *n* = 8 for both groups). **C** Bioluminescent imaging in SB28 tumors with RRV-RLI and anti-CD4 and anti-CD8 treatment. **D** In anti-CD4 and anti-CD8 antibody-treated mice bearing SB28 tumors, RRV-RLI does not significantly alter survival (median survival 16.5 days vs. 19 days, *p* = 0.44, Log-Rank Mantel-Cox, *n* = 7 PBS group, *n* = 8 RRV-RLI group) when compared to PBS treated mice. **E** Bioluminescent imaging in SB28 tumors with RRV-RLI and anti-CD8 treatment. **F** In anti-CD8 antibody-treated mice bearing SB28 tumors, RRV-RLI significantly increases survival (median survival 43.0 days vs. 18.0 days, *p* = 0.03, Log-Rank Mantel-Cox, *n* = 10 PBS group, *n* = 9 RRV-RLI group) when compared to PBS treated mice. **G** Bioluminescent imaging in SB28 tumors with RRV-RLI and anti-CD4 treatment. **H** In anti-CD4 antibody-treated mice bearing SB28 tumors, RRV-RLI significantly increases survival (median survival 44.5 days vs. 18.0 days, *p* = 0.0001, Log-Rank Mantel-Cox, *n* = 9 PBS group, *n* = 10 RRV-RLI group). **I** Bioluminescent imaging in SB28 tumors with RRV-RLI and anti-NK1.1 treatment. **J** In anti-NK1.1 antibody-treated mice bearing SB28 tumors, RRV-RLI significantly increases survival (median survival 42.5 days vs. 20.0 days, *p* = 0.0009, Log-Rank Mantel-Cox, *n* = 9 PBS group, *n* = 10 RRV-RLI group) when compared to PBS treated mice. *p < 0.05; **p < 0.01; ***p < 0.001; ****p < 0.0001. Data represent biological replicates. Source data are provided as a Source Data file.

We then investigated the role of T cells and their subtypes in the antitumoral effects of RRV-RLI in SB28 GBM treatment through antibody-mediated depletion studies. As expected, with isotype control depletion alone, we continued to see a therapeutic benefit from RRV-RLI treatment relative to PBS control (*p* < 0.005, Fig. 5A, B). However, when systemically depleting CD4^+^ and CD8^+^ T cells prior to tumor implantation and throughout the experiment after treatment, we observed complete abrogation of the RRV-RLI therapeutic benefit (*p* = 0.44, Fig. 5C, D). The efficacy of depletion was confirmed using the blood of tumor-bearing mice at 1 and 15 days after tumor implantation with reductions in CD3^+^, CD8^+^, and CD4^+^ populations to near undetectable levels (*p* < 0.05 for all populations, Supplementary Fig. 3A–C). To better understand immune cell populations contributing to the RRV-RLI therapeutic benefit we then investigated systemic CD8^+^ T cell depletion alone. In the setting of CD8 depletion, RRV-RLI treatment also continued to demonstrate a significant survival benefit (*p* < 0.05, Fig. 5E, F). Similarly, RRV-RLI treatment efficacy was unaffected by CD4 or NK1.1 depletion (*p* < 0.005, Fig. 5G–J). Between PBS treatment groups, survival differences in the various depletion cohorts were minor (Supplementary Fig. 3D). In the RRV-RLI treatment groups, the CD4/8 combined depletion demonstrated significantly reduced survival relative to all other depletion cohorts (Supplementary Fig. 3E).

### Anti-PD1 therapy fails to overcome tumor escape from RRV-RLI and treatment is dose dependent

Given the dependence of RRV-RLI on T cell-mediated antitumor immune responses, the observed upregulation of *Pdcd1* seen in our transcriptomic analysis, and the efficacy of anti-PD1 therapies in clinical practice^35^, we explored the therapeutic potential of combining RRV-RLI with anti-PD1 blockade. Consistent with the low immunogenicity of SB28 and its known resistance to checkpoint blockade^36^, systemic anti-PD1 treatment alongside PBS intracranial injection did not elicit a statistically significant improvement in bioluminescent imaging or survival in tumor-bearing mice compared to isotype control (median survival 20 days vs. 23 days, *p* = 0.132, Supplementary Fig. 3F–I).

We then assessed whether inhibiting PD-1 signaling on circulating and intratumoral T cells via systemic anti-PD1 therapy could enhance the therapeutic efficacy of intratumoral RRV-RLI in the SB28 murine GBM model. While there was a trend towards decreased bioluminescent tumor signal (*p* = 0.23, Supplementary Fig. 3H) and a higher percentage of long-term survivorship (20% vs. 10%, Supplementary Fig. 3I) this combination failed to yield a significant improvement in median survival or tumor regression when compared to RRV-RLI plus isotype control (median survival 43 days vs. 51.5 days, *p* = 0.699, Supplementary Fig. 3I), suggesting that, while RRV-RLI enhanced intratumoral immune cell infiltration and prolonged survival of GBM-bearing mice, PD-1 signaling alone was not a primary driver of treatment failure.

We next asked if RRV-RLI therapeutic efficacy is related to treatment timing and dose. Delaying RRV-RLI injection from day 4 to 7 after tumor implantation still improved survival relative to PBS control (*p* < 0.05, Supplementary Fig. 3J), although there was a trend towards reduced survival when injected 7 days after tumor vs. 4 days (*p* = 0.06, Supplementary Fig. 3J). Similarly, reducing the RRV-RLI treatment dose 10-fold still improved survival relative to PBS control (*p* < 0.05, Supplementary Fig. 3K), but the degree of survival improvement was less than that seen at the higher RRV-RLI treatment dose (*p* < 0.05, Supplementary Fig. 3K).

### Antitumoral efficacy of RRV-RLI against murine GBM is potentiated by systemic chemotherapy

**Fig. 6:**
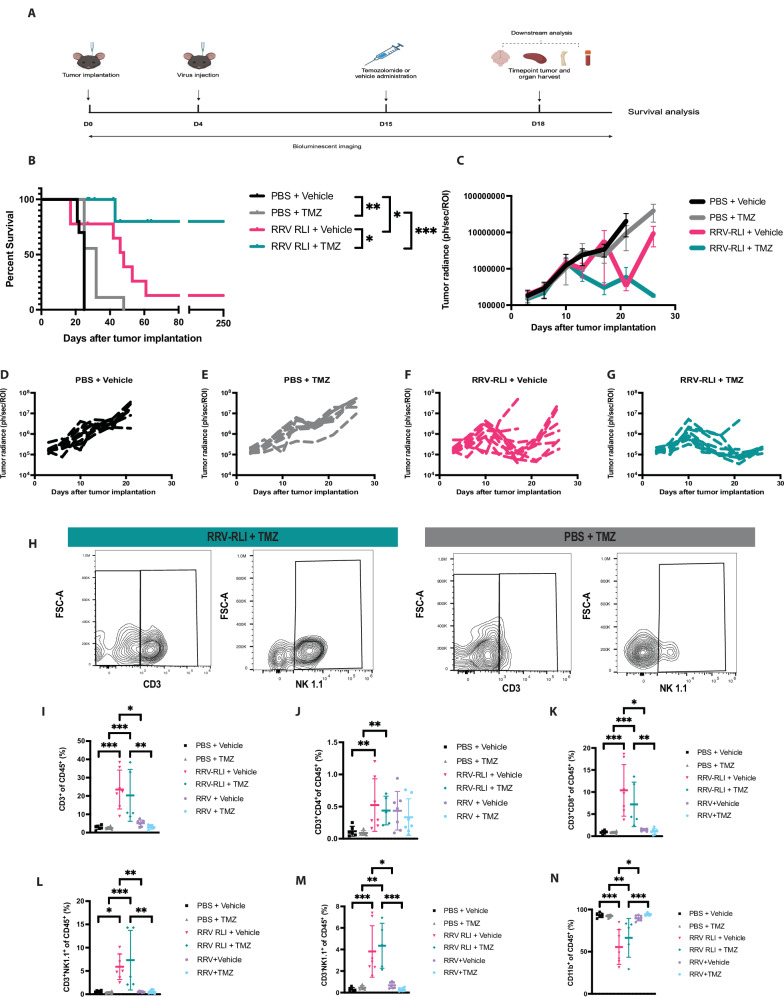
RRV-RLI synergizes with temozolomide chemotherapy to enhance treatment response with effector cell immune infiltration sustained despite systemic myelosuppression. **A** Schematic illustration of RRV-RLI in vivo assessment in temozolomide (TMZ) combination experiments. **B** RRV-RLI + TMZ improves survival relative to RRV-RLI + Vehicle (median survival undefined vs. 48 days; *p* = 0.03, Log-Rank Mantel-Cox test, *n* = 9 both groups) and PBS + TMZ (median survival undefined vs. 32 days; *p* = 0.0004, Log-Rank Mantel-Cox test, *n* = 9 both groups). Mice reaching non-tumor endpoint were censored at that time as shown (TMZ toxicity, *n* = 7). **C** Average bioluminescent tumor imaging. **D–G** Individual mouse bioluminescent imaging for all treatment groups (*n* = 10 PBS + Vehicle, *n* = 9 all other groups). **H** Example flow cytometric gating for CD3^+^ T cells (already gated on live CD45^+^CD11b^−^) as well as NK cells (already gated on live CD45^+^CD11b^−^CD3^−^) at day 18 post-implantation timepoint. **I** Percent tumor infiltrating CD3^+^ cells with significant increases in RRV-RLI vs. associated PBS and RRV treatment groups (RRV-RLI + Vehicle vs. PBS + Vehicle: *p* = 0.0005; RRV-RLI + TMZ vs. PBS + TMZ: *p* = 0.0003; RRV-RLI + Vehicle vs. RRV + Vehicle: *p* = 0.04; RRV-RLI + TMZ vs. RRV + TMZ: *p* = 0.004). **J** Percent tumor infiltrating CD3^+^CD4^+^ cells with significant increases in RRV-RLI vs. associated PBS treatment groups (RRV-RLI + Vehicle vs. PBS + Vehicle: *p* = 0.006; RRV-RLI + TMZ vs. PBS + TMZ: *p* = 0.005). **K** Percent tumor infiltrating CD3^+^CD8^+^ cells with significant increases in RRV-RLI vs. associated PBS and RRV treatment groups (RRV-RLI + Vehicle vs. PBS + Vehicle: *p* = 0.0002; RRV-RLI + TMZ vs. PBS + TMZ: *p* = 0.0009; RRV-RLI + Vehicle vs. RRV + Vehicle: *p* = 0.03; RRV-RLI + TMZ vs. RRV + TMZ: *p* = 0.005). **L** Percent tumor infiltrating CD3^+^NK1.1^+^ cells with significant increases in RRV-RLI vs. associated PBS and RRV treatment groups (RRV-RLI + Vehicle vs. PBS + Vehicle: *p* = 0.01; RRV-RLI + TMZ vs. PBS + TMZ: *p* = 0.0006; RRV-RLI + Vehicle vs. RRV + Vehicle: p = 0.003; RRV-RLI + TMZ vs. RRV + TMZ: *p* = 0.003). **M** Percent tumor infiltrating CD3^+^NK1.1^−-^ cells with significant increases in RRV-RLI vs. associated PBS and RRV treatment groups (RRV-RLI + Vehicle vs. PBS + Vehicle: *p* = 0.0001; RRV-RLI + TMZ vs. PBS + TMZ: *p* = 0.008; RRV-RLI + Vehicle vs. RRV + Vehicle: p = 0.04; RRV-RLI + TMZ vs. RRV + TMZ: *p* = 0.005). **N** Percent tumor infiltrating CD11b^+^ cells with significant decreases in RRV-RLI vs. associated PBS and RRV treatment groups (RRV-RLI + Vehicle vs. PBS + Vehicle: *p* = 0.0005; RRV-RLI + TMZ vs. PBS + TMZ: *p* = 0.01; RRV-RLI + Vehicle vs. RRV + Vehicle: *p* = 0.02; RRV-RLI + TMZ vs. RRV + TMZ: *p* = 0.0004). All flow cytometry samples were collected at day 18 post-implantation timepoint. *p < 0.05; **p < 0.01; ***p < 0.001; ****p < 0.0001. For flow cytometry data: *n* = 6 for PBS + Vehicle and PBS + TMZ groups, *n* = 7 for RRV-RLI + Vehicle and RRV + Vehicle and RRV + TMZ, *n* = 5 for RRV-RLI + TMZ. Data represent biological replicates. (**I**–**N**) graphs show mean +/− SD and utilize Kruskal-Wallis with uncorrected Dunn’s test post-hoc. Source data are provided as a Source Data file. (**A**) created in BioRender. Lab, A. (2026) https://BioRender.com/s4uhpg2.

Given the importance of antigen quality and dominance in orchestrating an effective antitumor immune response^37^, we hypothesized that temozolomide (TMZ) - a cytotoxic, DNA-damaging alkylating chemotherapy and the standard of care for newly diagnosed GBM - could enhance antigen availability in the tumor microenvironment (TME) by inducing tumor cell death. By combining RRV-RLI with systemically administered TMZ, we aimed to potentiate the T- and NK-cell infiltration and activation induced by RRV-RLI treatment (Fig. 6A). Indeed, the combination of systemic TMZ with intratumoral RRV-RLI resulted in improved survival relative to RRV-RLI + Vehicle (median survival undefined vs. 48 days, *p* = 0.03) or PBS injection with systemic TMZ monotherapy (median survival undefined vs. 32 days, *p* = 0.0004) (Fig. 6B) though there was expected TMZ toxicity given the high dose utilized (*n* = 7).

This combination benefit was further reflected in reductions of bioluminescent signal in the RRV-RLI + TMZ combination cohort relative to RRV-RLI + Vehicle and control groups (Fig. 6C–G). Flow cytometric analysis of intratumoral CD45^+^ immune cells revealed significantly enhanced infiltration of antitumoral T cell populations, including CD3^+^ and CD3^+^CD8^+^ T cells in the RRV-RLI treatment groups (RRV-RLI + Vehicle or RRV-RLI + TMZ) relative to PBS or RRV control groups (*p* < 0.05, Fig. 6H–K). T-regulatory cells (CD3^+^CD4^+^FOXP3^+^CD25^+^) were not a significant population in any treatment group.

In line with the mechanism of RLI, further flow cytometric analysis demonstrated increased populations of NKT and NK cells in the RRV-RLI treatment groups (Fig. 6L, M). Notably, there was no difference in T cell or NK-cell populations between RRV-RLI and RRV-RLI + TMZ treatment arms. A higher percentage of infiltrating CD3^+^ and CD3^+^CD8^+^ immune cells in RRV-RLI-treated mice demonstrated positive Ki67 expression relative to PBS and RRV control treatment groups (Supplementary Fig. 4B, gating strategy Supplementary Fig. 4A).

We next focused on the behavior of myeloid populations in the TME. RRV-RLI treated groups had significantly reduced infiltration of cells expressing the pan-myeloid marker CD11b with no difference between RRV-RLI + Vehicle and RRV-RLI + TMZ (Fig. 6N; gating strategy Supplementary Fig. 4C). Conventional dendritic cell (CD11b^+^ CD11c^+^ MHCII^+^) infiltration did not significantly differ between treatment groups (Supplementary Fig. 4D). Similarly, there was only a minor difference between RRV-RLI and PBS treatment groups in infiltrating CD11b^+^ CD11c^+^ MHCII^+^ CD8^+^ dendritic cells (*p* < 0.05, Supplementary Fig. 4E) with overall low infiltration and no difference between PBS and RRV groups (*p* > 0.05, Supplementary Fig. 4E).

Flow cytometric analysis of peripheral blood from treated mice demonstrated a significant reduction in live CD45^+^ cells per 100 uL of blood in the TMZ-treated cohort compared to vehicle-treated animals, consistent with the lymphodepletion commonly observed in patients undergoing TMZ therapy. This reduction extended to CD3^+^ and CD3^+^CD8^+^ populations when comparing RRV-RLI + Vehicle and RRV-RLI + TMZ (Supplementary Fig. 4F–H). These findings overall suggested that adding systemic TMZ to intratumoral RRV-RLI allowed the same levels of immune cell populations to infiltrate tumors and augmented the proliferation of these cells to the same degree despite the systemic myelosuppressive effects associated with TMZ treatment.

### Single-cell sequencing of intratumoral leukocytes after treatment with RRV-RLI and temozolomide reveals potent antitumor immune modulation

**Fig. 7:**
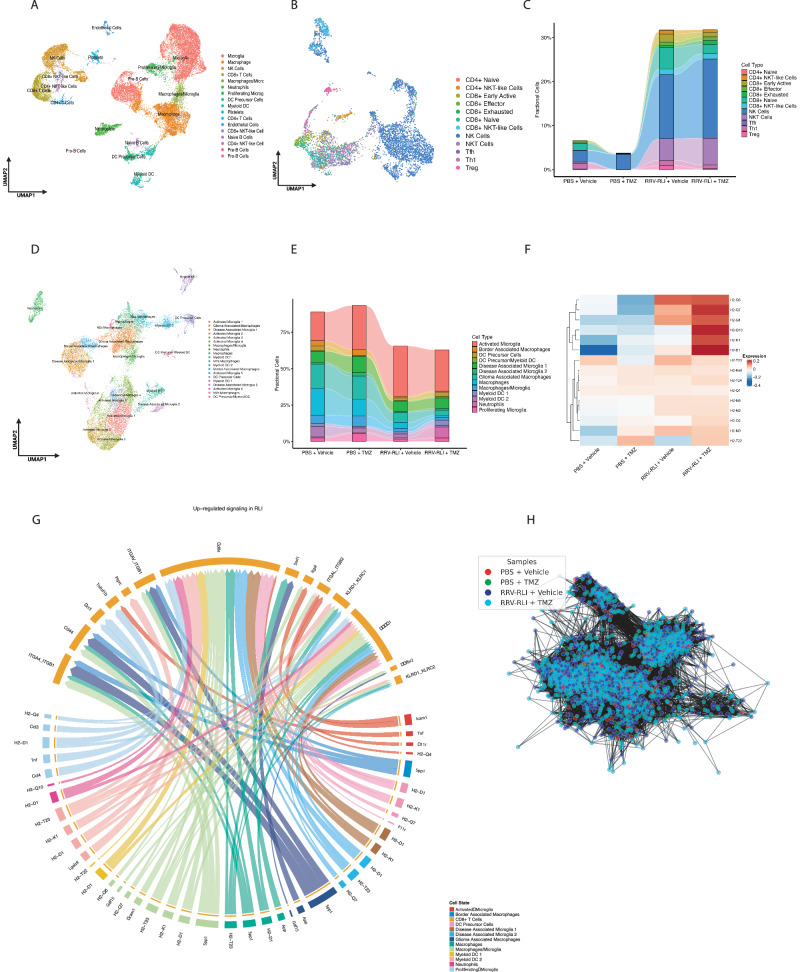
Single-cell RNA sequencing reveals cellular mechanisms driving therapeutic efficacy and its enhancement with TMZ. **A** Single-cell RNA sequencing UMAP of all sequenced transcriptomes from CD45^+^ cells isolated from SB28 tumor-bearing mice, demonstrating myeloid, T, and NK cell clusters. **B** T and NK cell focused UMAP including all samples. **C** Alluvial plot demonstrating changes in gross T and NK cell infiltration between samples. **D** Myeloid cell-focused UMAP including all samples. **E** Alluvial plot demonstrating changes in gross myeloid cell infiltration between samples. **F** Heat map of MHC class I expression in myeloid populations across samples highlighting increased expression in RRV-RLI + Vehicle and enhancement in RRV-RLI + TMZ. **G** Cell chat analysis of incoming signaling in RRV-RLI + Vehicle vs. PBS + Vehicle. **H** Beta chain clustering of TCRs from all samples within 80 distance units with labeling of sample specific TCRs. Data represent FACS-sorted CD45^+^ cells from a mouse per condition, infiltration data validated by flow cytometric analysis across independent biological replicates (Fig. 6I–N). See data availability for GEO information.

To understand how TMZ was potentiating the effects of RRV-RLI without altering levels of the immune cell populations we interrogated by flow cytometry, we performed 5’ single-cell sequencing of CD45^+^ leukocytes, isolated by fluorescence-activated cell sorting from SB28 GBMs treated with intratumoral RRV-RLI and/or systemic temozolomide. After processing, a total of 28,125 cells were annotated into 17 different clusters (Fig. 7A). T and NK cells were then relabeled into 12 phenotypic clusters based on predetermined markers (Fig. 7B), with the distribution of critical gene markers provided in Supplementary Fig. 5A. RRV-RLI treatment groups exhibited higher levels of T and NK cell infiltration as compared to PBS control groups, with marked expansions in specific clusters including NKT cells, NK cells, and CD8^+^ T cell populations (Fig. 7C).

Differential gene expression analysis of tumor-infiltrating CD8^+^ T cells between RRV-RLI + TMZ and RRV-RLI + Vehicle demonstrated upregulation of genes indicative of cytotoxic immune activity and antitumor immunity including *Gzmb* (log2FC = 0.67, padj = 2.13e-12)*, Gzmc* (log2FC = 2.23, padj = 1.5e-14)*, Gzmd* (log2FC = 5.72, padj = 1.32e-10)*, Gzme* (log2FC = 6.27, padj = 2.53e-7)*, Gzmf* (log2FC = 6.81, padj=4.72e-17), and *Gzmg* (log2FC = 8.50, padj = 4.14e-7) (Supplementary Fig. 5B). Additionally, there was notable downregulation of *Tox* (log2FC = −1.0, padj=4.45e-5), an HMG-box transcription factor that transcriptionally and epigenetically programs CD8^+^ T cell exhaustion such that *Tox* downregulation is a sign of increased T cell activation (Supplementary Fig. 5B)^38^. Further gene set enrichment analysis (GSEA) revealed enrichment of biological processes related to chemotaxis, cell killing, and response to chemokines in RRV-RLI + TMZ as compared to RRV-RLI + Vehicle (Supplementary Fig. 5B). Similar trends in gene expression and GSEA were seen when comparing NKT cells between these groups (Supplementary Fig. 5C)^37^. Together, these findings highlight the synergistic effects of temozolomide and RRV-RLI in enhancing cytotoxic immune activity, reducing T cell exhaustion, and promoting antitumor immune processes within the GBM TME.

Attention was then turned to myeloid populations across the different treatment groups, identifying 20 different myeloid sub-clusters using scType^39^ and expression of canonical marker genes (Fig. 7D, Supplementary Fig. 5D)^40–42^. As observed in flow cytometric analyses (Fig. 6N), myeloid populations were a lower fraction of total CD45^+^ cells in RRV-RLI treatment groups, with a disproportionate decrease in the macrophage population when compared to PBS groups (Fig. 7E). However, MHC class I gene expression was increased in RRV-RLI treatment groups, with even higher expression overall in the RRV-RLI + TMZ combination. This increase was particularly evident in genes including *H2-q6, H2-q7, H2-q4, H2-q10, H2-k1, H2-d1* (Fig. 7F). Specific myeloid cell populations with increased MHC class I gene expression varied but included myeloid DC 1 and 2 cells, neutrophils, and precursor myeloid DC cells (Supplementary Fig. 5E, J). Additional differential gene expression of tumor-infiltrating myeloid cells, demonstrated a shift toward reactive phagocytic macrophages, mature activated dendritic cells and a reduction in the immunosuppressive myeloid-derived suppressor cell (MDSC) neutrophil subset with RRV-RLI treatment (Supplementary Fig. 5F–H). In addition, dendritic cells demonstrated a further increase in their antigen presentation machinery in RRV-RLI + TMZ vs. RRV-RLI + Vehicle groups beyond the increase already occurring with RRV-RLI treatment alone (Supplementary Fig. 5I). These findings supported the hypothesis that TMZ-induced cell death and antigen release led to enhanced antigen-presentation by myeloid cells in the TME in the setting of RRV-RLI treatment.

To assess potential interactions between these gene expression changes we conducted a ligand-receptor analysis using CellChat. Comparing CD8^+^ T cells between RRV-RLI + Vehicle and PBS + Vehicle identified increased receptor-ligand signaling dominated by antigen presentation signaling primarily through MHC class I proteins from multiple myeloid cell populations (including neutrophils, myeloid DC 1, myeloid DC 2, and macrophages/microglia) to the CD8^+^ T cell population (Fig. 7G) suggestive of cross-presentation of antigens. This antigen presentation signaling was further amplified when comparing CD8^+^ T cells between RRV-RLI + TMZ vs. RRV-RLI + Vehicle (Supplementary Fig. 5G). Inferred SPP1 signaling, a marker of glioma associated macrophages, which may represent migratory or counter-regulatory pathways was also increased in both comparisons (Fig. 7G and Supplementary Fig. 5G)^43–45^. Subsequent in vitro T cell priming assays utilizing SB28 OVA cell lines and OT-1 CD8^+^ T cells demonstrated significantly higher T cell activation (CD69^+^, *p* < 0.05) and division (Ki67^+^, *p* < 0.05) in groups treated with TMZ (Supplementary Fig. 6A, B). These results demonstrate that the combination of temozolomide with RRV-RLI enhances myeloid cell-mediated antigen presentation, likely amplifies cross-presentation to CD8 + T cells, and drives robust immune activity in the TME.

### T-cell receptor sequencing suggests tumor antigen-specific T-cell responses induced by RRV-RLI treatment and potentiated by the addition of TMZ to RRV-RLI

To further elucidate the impact of each treatment on the T-cell receptor (TCR) repertoire, TCR sequencing was performed on tumor-infiltrating T cells. Comparison of TCR repertoire between treatment groups revealed over double the percent recurrent TCRs sequenced in the RRV-RLI + TMZ treatment group vs. the RRV-RLI + Vehicle group (15.3% vs. 7.2% Supplementary Fig. 6C), suggestive of clonal expansion and an antigen-driven immune response^46^. This was further supported by a higher percentage of the TCR repertoire space being occupied by recurrent clonotypes in tumors treated with RRV-RLI + TMZ vs. RRV-RLI + Vehicle (Supplementary Fig. 6D), indicating that combining RRV-RLI and TMZ enhanced the antigen-specific immune response relative to RRV-RLI alone.

T-cell receptor (TCR) clustering was performed using CDR3 beta-chain sequences from all samples. We found that 1810 out of 1813 TCRs were within 80 distance units of each other (Supplementary Fig. 6E). The top 10 clusters accounted for 91.5% of the sequenced TCRs (1659 out of 1813 TCRs), and each of these clusters included TCRs from a PBS-treated control group (non-virus exposed, Fig. 7H and Supplementary Fig. 6F), suggesting that the majority of TCRs after treatment were directed against tumoral antigens rather than viral antigens.

To further assess how much of the response to RRV-RLI was directed against tumoral versus viral antigens, we then assessed the similarity between our TCR sequences and those specific to pathogens and murine leukemia virus (MLV) as reported in the McPAS-TCR database^47^. In total, 42 out of 1813 beta chain TCRs (2.3%) were within 18 distance units of a pathogen-specific TCRs from McPAS-TCR. Notably, none of the TCRs reactive to the envelope protein of MLV were within 100 distance units for paired chains or 18 distance units for beta chains in our samples.

Subsequently, we performed exact matching on the beta chain TCR data. There were 45 exact matches across all TCR sequences. Importantly, no exact matches were found in our data for beta chains of TCRs reactive to MLV envelope proteins. We identified a total of 24 exact matches corresponding to pathogen-specific TCRs, which mapped to herpes simplex virus type 1 (HSV-1), influenza virus, and murine cytomegalovirus (mCMV). In paired alpha-beta chain analysis, we have identified 1/1364 exact alpha-beta matches to a pathogen in McPAS (influenza) and 35/1364 alpha-beta pairs within 100 unit distance of McPAS pathogen antigens. Together, these findings suggested that the TCRs we had identified were part of a tumor-specific response enhanced by RRV-RLI and RRV-RLI + TMZ rather than an antiviral response triggered by RRV-RLI.

## Discussion

The IL-15 superagonist RLI is a potent immunostimulatory agent with the ability to enhance an antitumor immune response through T- and NK-cell modulation. Our study evaluates RRV-RLI as a viral immunotherapy for the treatment of GBM. We demonstrate that tumor cells infected with RRV-RLI produce functional RLI with canonical functions such as supporting T- and NK-cell proliferation. Single-agent intratumoral RRV-RLI treatment resulted in significantly improved survival in the immunosuppressive SB28 model and long-term survival with immunologic memory to contralateral orthotopic rechallenge in the Tu2449 GBM model. RRV-RLI also synergizes with GBM standard of care in the form of TMZ chemotherapy to enhance antitumor immunity and improve survival. RRV is a highly translatable vector platform with a strong safety profile in patients even when delivered intravenously^24^. This work establishes RRV-RLI as a translatable viral cancer immunotherapy underscoring its potential for clinical application in humans.

IL-15 is regarded as a promising immunocytokine for cancer immunotherapy^48^. While other cytokines, such as IL-2, promote T cell growth, they also act as a ‘double-edged sword’ by concurrently upregulating T-regulatory cells and triggering activation-induced cell death (AICD) or capillary leak syndrome. In comparison, IL-15 supports the proliferation and activation of CD8^+^ T cells (including memory phenotype), NKT cells, and NK cells^49–52^. Moreover, IL-15 induces pro-inflammatory changes within the tumor microenvironment and has demonstrated synergy when combined with other immune and chemotherapeutic agents^53,54^. Interestingly, IL-15 also reprograms the myeloid compartment, reducing immunosuppressive myeloid populations, promoting inflammatory signaling in macrophages, and skewing intratumoral immune cell proportions toward T and NK cells at the expense of myeloid cells^55,56^.

Local delivery of immunotherapies directly to the TME mitigates the dose-limiting toxicities associated with systemic administration. In the brain, this approach also circumvents the blood-brain barrier (or blood-tumor barrier), a major obstacle for many systemically administered treatments. In this study, we aimed to transform GBM tumor cells into biofactories for RLI through delivery via RRV, a virus that selectively spreads in dividing cells without inherently killing host cells. Our rationale was that this strategy would create a portion of tumor cells secreting RRV-RLI and a field effect across the tumor with the goal of in situ tumor vaccination.

We observed that local delivery and tumor-mediated secretion of RLI in intracranial tumors produced outcomes in line with or superior to those observed with systemic administration of RLI in other cancer types or ALT-803, an alternative IL-15 superagonist, in GBM^26–28^. An advantage of our study is that our findings were in two syngeneic GBM models with best-in-class translatability with regard to the investigation of immunotherapies^36^. Directed tumor delivery of RRV-RLI led to a significant survival benefit in both the SB28 and Tu2449 GBM models. Notably, treated Tu2449 mice exhibited over 85% long-term survivorship and developed immunologic memory upon intracranial rechallenge with uninfected Tu2449 cells, effectively demonstrating successful vaccination against tumor cells, rather than viral antigens. We attribute the differential response to single-agent RRV-RLI treatment between the two models to variations in their underlying tumor immunogenicity. While Tu2449 is also poorly immunogenic relative to most murine GBM models, it does exhibit higher T cell infiltration and a higher (but still low) baseline rejection rate compared to the SB28 model, indicating increased immunogenicity. SB28 being one of the least immunogenic models makes it more representative of human GBM, characterized by low T cell infiltration and a low tumor mutational burden^36,57^. RRV-RLI treatment alone significantly increased CD8 T cell, NKT cell, and NK-cell infiltration in treated SB28 mice from near undetectable levels. We believe these findings are promising for the treatment of human GBM, where CD8^+^ T cell infiltration is estimated to be under 5% of tumor-infiltrating immune cells, similar to the SB28 model^58^. RRV-RLI treatment also induced broad transcriptomic inflammatory changes within the TME and upregulated antigen-presenting pathways. Transcriptomic and flow cytometric analyses largely demonstrated an increase in effector immune populations, including improved “exhausted” to cytotoxic T cell ratios, in the RRV-RLI treatment groups relative to both PBS and RRV controls. This occurred without a large increase in dendritic cells on flow cytometric analysis, suggesting a modification of existing antigen-presenting cell populations rather than driving additional cell infiltration. These findings align with existing literature highlighting the role of IL-15 in the maturation and function of antigen-presenting cells^59^. Collectively, this manipulation of the local TME enhanced the immunogenicity of an otherwise immunologically silent tumor.

Given the increased infiltration of both T cell and NK-cell populations in the TME, we sought to examine the critical mediators of the RRV-RLI treatment response. We performed these depletion experiments using RRV-RLI versus PBS because RRV treatment provided no survival benefit and had a similar immunologic profile on transcriptomic and flow cytometric analysis to PBS, whereas both had marked differences with RRV-RLI treatment. As such, a comparison between RRV-RLI and PBS is likely more clinically relevant. Simultaneous depletion of both CD4^+^ and CD8^+^ T cells abrogated the survival benefit imbued by RRV-RLI treatment, indicating a reliance on those T cell populations for treatment efficacy, despite increased NK-cell infiltration in treated tumors. This is in line with previous work using ALT-803 in GBM, which saw a reduction in treatment efficacy with CD4^+^ or CD8^+^ T cell depletion, but not with NK-cell depletion^60^. The role of NK-cells in mediating antitumor efficacy in response to systemic IL-15 immunotherapy has varied, with some cancer models demonstrating a dependence on NK-cells^61^ and others proving reliant only on tissue-resident CD8^+^ T cells^27,62^. Interestingly, the therapeutic benefit of RRV-RLI continued even in the setting of CD8, CD4, and NK depletion. Because abrogation of RRV-RLI treatment efficacy only occurred with combined CD4 and CD8 depletion, we believe this may suggest a dynamic compensatory role for CD4^+^ T cells and NK cells in the RRV-RLI response in the absence of CD8^+^ T cells. Furthermore, our findings suggest that CD8^+^ and CD4^+^ T cells coordinate to drive RRV-RLI therapeutic efficacy. Both CD8^+^ and CD4^+^ T cells are needed for the survival benefit, such that the therapeutic benefit can continue as long as one of these T cell subtypes is present, but is lost once both are gone. This versatility has implications for the translatability of RRV-RLI to the clinic, where patients may be immunosuppressed from previous treatments. Differences seen in the RRV-RLI survival benefit with reduced dosing or delayed timing suggested a kinetic constraint for the virus to reach adequate tumor transduction given the fast-growing SB28 model. Even so, these data provide valuable information for clinical dosing.

An inherent challenge to the translation of immunotherapies to GBM is understanding interactions with standard of care, especially temozolomide, which causes myelosuppression as a known dose-limiting toxicity^63,64^. Interestingly, the efficacy of RRV-RLI treatment was maintained, and antitumor immunity even enhanced, with concurrent temozolomide treatment. Flow cytometric analysis of the TME revealed no gross differences in immune cell infiltration between the RRV-RLI monotherapy and the combination therapy with high-dose temozolomide, highlighting the sustained presence of tumor-infiltrating T cells despite systemic myelosuppression. This has important implications for clinical translation given the widespread use of TMZ in patients. 5’ single-cell RNA sequencing of the treatment groups also revealed similar immune cell infiltration, consistent with the flow cytometry, with no difference between RRV-RLI alone and with TMZ. Interestingly, RRV-RLI treatment appeared to favor activation of T cells and NKT cells, evidenced by the upregulation of cytotoxicity-associated genes such as *Gzmb*, and the downregulation of *Tox*, an HMG-box transcription factor that is a central regulator transcriptionally and epigenetically programming CD8 + T cell exhaustion^38,65^. Additionally, single-cell T -cell receptor (TCR) sequencing revealed more than double the percentage of recurrent TCR clonotypes in the RRV-RLI + temozolomide (TMZ) group, indicative of a robust antigen-specific response. While a viral specific immune response cannot be excluded, the presence of non-virus exposed samples (PBS and PBS + TMZ) in the majority of dominant TCR clusters in combination with the paucity of MLV and pathogen-specific TCR matches or similarities support a tumor-specific reaction rather than one against the viral therapy. The observed immunologic memory against untreated tumors in the Tu2449 model also supports a tumor specific response. These findings, coupled with the increased expression of MHC class I genes in myeloid populations within both the RRV-RLI + Vehicle and RRV-RLI + TMZ groups, support the hypothesis that TMZ-induced cell death and tumor antigen release in the TME provides additional antigenic material for antigen-presenting myeloid cells. These cells, likely further activated by local RLI expression, facilitate cross-presentation to CD8^+^ T cells. This is further supported by CellChat analysis, which demonstrated increased MHC class I-CD8 T cell receptor-ligand interactions in the RRV-RLI + Vehicle group compared to control, with even greater enhancement observed in the RRV-RLI + TMZ group compared to RRV-RLI + Vehicle. In addition, in vitro cross-presentation experiments demonstrated increased antigen presence and cross-presentation after treatment with TMZ. These data support a model where TMZ cell death increases antigen supply which then synergizes with RRV-RLI enhancement of T cell, NK cell, and antigen presentation responses. We utilized TMZ at a dose on the higher end of reported preclinical ranges to increase biological insight into the combination with RRV-RLI and model clinically relevant myelosuppression, which resulted in expected TMZ-related toxicity^66^. Future investigation into the combination of RRV-RLI and standard of care, including radiation and TMZ dosing, is warranted. In addition, the combination of RRV-RLI with other potentially synergistic modalities of inducing cell death and antigen release with lower off-target toxicity should be considered.

Others have employed various oncolytic platforms, such as herpesviruses (HSV), vesicular stomatitis virus (VSV), and poxviruses, to deliver local immunotherapies to tumors^67–69^. Although these vectors can replicate in neoplastic tissue, their lytic nature leads to early tumor cell destruction and can trigger robust antiviral host responses - such as NK-cell activity and type I interferon signaling - that limit the duration and breadth of intratumoral transgene delivery^70,71^. Recent work in VSV has demonstrated the generation of antiviral T-cell responses that can, in fact, detract from concurrent immune checkpoint inhibition^72^. Previous work has also demonstrated improved delivery of an HSV oncolytic platform with pre-delivery immunosuppression via TGF-β administration^73^. In contrast, our non-lytic replicating retrovirus can suppress type I interferon signaling and spreads throughout the tumor mass without inducing immediate cell death or a robust antiviral response, enabling sustained and uniform intratumoral delivery of the therapeutic transgene^21^. We achieved over 85% tumor cell infection in orthotopic GBM models by 18 days post-injection and do not see evidence of an antiviral immune response in our TCR sequencing. Furthermore, non-replicating viral vectors such as adeno-associated viruses (AAVs) and lentiviruses, while safe and stable, lack the capacity for in situ amplification in rapidly dividing tumor cells. As a result, these non-replicating vectors cannot effectively scale with tumor growth and have difficulty reaching a significant number of tumor cells, limiting their ability to achieve extensive and persistent therapeutic gene expression. Our use of a non-lytic, replicating retroviral platform addresses key limitations of both lytic oncolytic viruses and non-replicating vectors for IL-15 delivery in the challenging context of solid tumors.

This study positions RRV-RLI as a potent and clinically translatable viral immunotherapy for GBM, showcasing its ability to drive robust activation of T cells, NK-cells, and enhanced antigen presentation in the TME. Treatment with RRV-RLI significantly extends survival in two distinct syngeneic murine GBM models and synergizes effectively with the current standard of care chemotherapy. Our TCR-sequencing demonstrating the lack of immunogenicity of the RRV backbone supports RRV as a delivery vehicle for the potent RLI transgene and supports the tolerability of repeat treatments in future studies. Overall, our findings provide compelling preclinical evidence to support a Phase I clinical trial to evaluate the safety and efficacy of RRV-RLI in human patients. Moreover, the versatility of this therapeutic approach holds promise for broader application across other cancer types, offering a different approach to the treatment of solid tumors.

## Methods

### Study design

The objective of this study was to assess the therapeutic potential of RRV-RLI and to elucidate the underlying biological mechanisms driving its efficacy. For in vivo experiments, 8-12 week-old female mice were utilized, with 6 to 10 mice per group, ensuring adequate statistical power based on preliminary data. Mice were randomized into treatment arms following tumor implantation, with randomization based on bioluminescent imaging to equalize the average starting tumor sizes across groups. All experiments adhered to Institutional Animal Care and Use Committee (IACUC) guidelines, with predetermined survival endpoints applied. Mice reaching non-tumor endpoints were censored from survival analyses.

### Construction of RRV-RLI

Gibson assembly cloning was used to place a codon- and stability-optimized genetic sequence for human RLI^28^ into pAC3-P2A-yCD. A T2A cleavage peptide sequence was positioned at the C-terminus of the viral envelope protein followed by the RLI sequence (Fig. 1I).

### Cell lines and culture

Human embryonic kidney 293T (Lenti-X cells, purchased from Takara, Inc.), murine glioblastoma Tu2449 (generously provided by Dr. Noriyuki Kasahara, University of California, San Francisco), and human glioblastoma U87 (generously provided by Dr. Noriyuki Kasahara, University of California, San Francisco) were cultured in Dulbecco’s modified Eagle’s medium (DMEM) supplemented with 10% fetal bovine serum (FBS) and 1X Gibco GlutaMAX (Gibco, Inc.). Murine glioblastoma SB28 (generously provided by Dr. Hideho Okada, University of California, San Francisco) were cultured in RPMI 1640 medium supplemented with 10% FBS, 2% Gibco GlutaMAX, 1% non-essential amino acids (Gibco, Inc.), 1% HEPES (Gibco, Inc.), 1% Penicillin-Streptomycin (Gibco, Inc.), and 0.1% beta-mercaptoethanol. Cells were screened bimonthly for mycoplasma and validated every six months by Short Tandem Repeat (STR) analysis at the University of California Cell Culture Facility. Material requests can be sent to the corresponding author.

### Viral production and concentration

Virus was produced via transient transfection and the use of stable producer cell lines. For transient transfection protocols, reverse transfection utilizing Fugene HD (Promega) and 10 micrograms of viral plasmid DNA was implemented. Stable U87 producer cell lines were also utilized for viral production. Virus-containing supernatant was collected at approximately 36-48 hours after initial transfection. For in vivo studies virus was concentrated using column-based retrovirus purification with buffer exchange to phosphate buffer solution (PBS) (Bioland Scientific LLC.). Functional viral titers in transducing units per mL (TU/mL) were determined via staining for the viral gag-protein and flow-cytometry.

### In vitro viral replication and stability

Viral replication and stability were performed in a similar manner to previous studies^74^. In brief, specific multiplicities of infection (MOI) were added to tumor cells in vitro and allowed to replicate over time, with transduction levels measured at regular intervals via staining for the gag viral protein and flow cytometry. Azidothymidine, which inhibits viral spread, was utilized for control groups. For viral stability, an MOI of 0.01 was added to tumor cells and allowed to spread to 100% transduced cells at 14 days. The resulting supernatant was then placed on tumor cells at an MOI of 0.01, and the protocol was repeated for a total of 8 weeks. Genomic DNA was then isolated using the Monarch® Genomic DNA Purification Kit (NEB Biolabs). Polymerase chain reaction with primers flanking the RLI insert was used to determine stability of the construct. Primer sequences were as follows (5’−3’): Forward: ggaccttgcattctcaatcgattgg Reverse: cccctttttctggagactaaataa.

### RLI production and function

RLI production levels were determined using supernatant from 100% infected SB28 tumor cells. RLI levels in the supernatant were measured via Human IL-15 Quantikine enzyme-linked immunosorbent assay (R&D Systems). RLI function was determined using the CTLL-2 proliferation assay similarly to previous studies^30^. In brief, RLI from transduced cells was added to cytokine-starved CTLL-2 cells with proliferation of these cells subsequently measured via Colorimetric MTS Assay (Promega). Co-culture assays were conducted by incubating SB28 tumor cells with freshly isolated NK cells (BioLegend MojoSort™ NK Cell Isolation Kit) or CD8^+^ T cells (BioLegend MojoSort™ CD8 T Cell Isolation Kit) from C57BL/6 spleens. Tumor and effector cells were co-cultured at varying tumor-to-effector cell ratios and conditions. Activation and exhaustion markers were measured via flow cytometric analysis.

### In vitro antigen presentation assay

The protocol used was similar to those previously described in the literature^75^. SB28 tumor cells (both OVA expressing and wildtype) were seeded then 16 h later exposed to vehicle or temozolomide (1 mM) for 24 h. Tumor media was then refreshed, and the cells allowed to incubate for an additional 48 h. Finally, supernatant was harvested from the various treatment groups, mixed with dendritic cells (BioLegend MojoSort™ Pan Dendritic Cell Isolation Kit) freshly isolated from C57BL/6 spleens, then allowed to incubate overnight. The next day, CD8^+^ T cells (BioLegend MojoSort™ CD8 T Cell Isolation Kit) isolated from OT-1 mouse spleens were mixed with the aforementioned supernatant-exposed dendritic cells at a 1:1 ratio. This mixture was allowed to incubate for 60 h prior to flow cytometric analysis for markers for T cell activation.

### Animal studies

Animal experiments were approved by the University of California, San Francisco Institutional Animal Care and Use Committee (IACUC protocol #AN105170-02). C57BL/6 (Jax # 000664) and B6C3F1 (Jax #100010) mice (8–12 weeks old) were obtained from Jackson Laboratories and housed in a standard specific pathogen-free facility at the University of California, San Francisco. Mice from different treatment groups were housed separately. Experiments utilizing Tu2449 and SB28 murine glioblastoma cell lines were conducted under comparable conditions. All cell lines expressed luciferase to facilitate bioluminescent imaging. On day 0, 10,000 tumor cells were implanted intracranially using a stereotactic frame at the following coordinates relative to the bregma: anteroposterior (AP), 0 mm; mediolateral (ML), 1.2 mm; and dorsoventral (DV), 3.5 mm. Mice were imaged on day 3 or day 4 post-tumor injection to establish pretreatment bioluminescent baselines, after which they were randomized into treatment groups. On day 4 post-tumor implantation, mice were injected with 2.5 × 10^5 TU of either RRV-RLI, control RRV, or PBS, depending on the assigned treatment group, unless otherwise specified. In the Tu2449 rechallenge experiment, untreated age-matched tumor bearing mice were utilized as the comparator group. Mice were injected with the assigned treatment on day 7 for experiments requiring delayed injection. In vivo viral genome presence or RLI expression were determined using qPCR on isolated tissue genomic DNA or IL-15 ELISA (R&D Systems), respectively. Samples comparing tumor to contralateral brain were taken from mice injected with 2.5 × 10^4 TU as described in Supplementary Fig. 3D. Female mice were used to reduce variability in tumor growth kinetics. Sex was not considered a design variable for this study. Primers utilized for virus detection via qPCR are as follows (5’ to 3’): Virus probe: ccccaaatgaaagacccccgctgacg. Virus forward: agcccacaacccctcactc. Virus reverse: tctcccgatcccggacga.

For the anti-PD1 combination experiments, mice were administered either anti-PD1 antibody (RMP1-14, BioXcell) or isotype control (BioXcell) at a dose of 200 µg, delivered via intraperitoneal injection on day 7 and day 24 post-tumor implantation, with subsequent dosing every other day for a total of four doses. In the TMZ combination experiments, mice were treated with temozolomide (TMZ) at a total dose of 400 mg/kg, administered over three days (diluted in 10% DMSO, T2577 Sigma Aldrich) starting on day 15 post-tumor implantation, or with vehicle control (10% DMSO).

For T cell depletion studies, depletion antibodies were administered two days prior to tumor implantation and subsequently continued biweekly post-implantation. For the combined CD4 and CD8 depletion, a total of 400 µg of anti-CD8 antibody (200 µg YTS 169.4, BioXcell; 200 µg 53-6.7, BioXcell) and 200 µg of anti-CD4 antibody (GK1.5, BioXcell) were administered. For the CD8 depletion, a total of 400 µg of anti-CD8 antibody (200 µg YTS 169.4, BioXcell; 200 µg 53-6.7, BioXcell) was administered. In CD4 depletion alone, 200 µg of anti-CD4 antibody (GK1.5, BioXcell) was administered. In NK1.1 depletion alone, 200 µg of anti-NK1.1 antibody (PK136, BioXcell) was administered. Control mice received 600 µg of isotype antibody (LTF-2, BioXcell). Depletion was confirmed on days 1 and 15 after tumor implantation. CD8^+^ dendritic cell populations were not investigated in the setting of depletion given their low abundance in the tumor microenvironment (Supplementary Fig. 4E). Survival data were plotted using the Kaplan-Meier method, with statistical comparisons between groups performed using the Log-rank test (GraphPad Prism 10).

### Analysis of tumor microenvironment immune alterations

Mice were euthanized at endpoint, day 14 post-tumor injection timepoint (non-TMZ studies), or day 18 timepoints (TMZ studies) via CO_2_ inhalation followed by cervical dislocation. The following tissues were harvested: spleen, bone marrow, blood, and brain tumor. Brain tumors were minced and digested in collagenase type IV (Thermo Fisher Scientific, #17104019) and Deoxyribonuclease I (Worthington Biochemical Corporation) solutions while agitated at 37 °C. Tumor suspensions were subsequently filtered through 70 µm filters, and red blood cells were lysed using Ammonium-Chloride-Potassium (ACK) lysing buffer (Lonza). Spleens were dissociated through 40 µm filters and similarly subjected to ACK lysis, as was the bone marrow.

Flow cytometric analysis was then performed on processed tissues. A detailed list of antibodies can be found in Supplementary Table 1. Briefly, cells were first blocked with mouse Fc block in PBS containing 2% fetal bovine serum (FBS). After Fc blocking, cells were washed and stained with Zombie Aqua fixable viability dye (BioLegend, #423101) in PBS. Following viability staining, cells were washed again and stained for surface markers in PBS with 2% FBS. Intracellular marker staining was performed using the eBioscience™ Foxp3/Transcription Factor Staining Buffer Set (Thermo Fisher Scientific, #00-5523-00). Data acquisition was conducted using an Attune NxT Flow Cytometer (Thermo Fisher Scientific), and flow cytometry data were analyzed using FlowJo software.

### Nanostring multiplex transcriptomic analysis

RNA was extracted from murine tumor single-cell suspensions using the RNeasy Mini kit (Qiagen) and stored at −80 °C until further use. RNA quality and quantity were assessed using a bioanalyzer. For each sample, 100 ng of RNA was hybridized with the Nanostring Mouse PanCancer Immune Profiling codeset for 18 h. A 30 µL aliquot of the reaction was loaded into the nCounter cartridge and processed on the nCounter SPRINT Profiler. Quality control and raw data alignment were conducted using nSolver (Nanostring).

Differential gene expression analysis was performed using the DESeq2 package in R, followed by pathway expression analysis as defined by the KEGG 2019 Human database, utilizing the Enrichr pipeline. Simultaneously, raw files were analyzed on the Rosalind online platform (OnRamp Bio) to calculate normalized gene expression counts, determine significance of differentially expressed genes, and derive cell type scores. Visualizations, including heatmaps and volcano plots, were generated using GraphPad Prism 10.

### Mouse scRNA sequencing and immune cell profiling analysis

Brain tumors were processed as previously described on day 18 after tumor implantation. CD45^+^ cells were isolated via fluorescence-activated cell sorting. 5’ scRNA-seq with TCR sequencing was carried out using the 10X Genomics platform and per manufacturer instructions. Sequencing was performed on the Illumina Novaseq 6000. All samples were processed and sequenced in a single batch.

Single-cell FASTQ files, along with paired TCR V(D)J immune profiling FASTQ files, were aligned using CellRanger (version 7.2, 10X Genomics). The resulting expression matrices were imported into R (version 4.3.2) for downstream analysis. Standard pre-processing was conducted using Seurat (version 5.0.3). The dataset included 2,928 cells in the PBS condition, 8,716 cells in the TMZ condition, 6,225 cells in the RRV-RLI + Vehicle condition, and 10,872 cells in the RRV-RLI + TMZ condition. Cells expressing more than 200 genes with less than 10% mitochondrial gene expression were retained for further analysis.

Samples were integrated using the standard Seurat v5 workflow. In total, 28,125 cells were assigned across 17 clusters. Cell types were identified using a combination of suggested labels from scType and marker gene expression based on literature review. Alluvial bar graphs showing sample composition by cell type were created using ggalluvial (version 0.12.5).

The integrated samples were divided into lymphocyte and myeloid subsets based on cluster identity. The lymphocyte subset included cells labeled as NK Cells, CD4 + T Cells, CD8 + T Cells, CD8 + NKT-like Cells, and CD4 + NKT-like Cells, while the myeloid subset contained Macrophages, Microglia, Proliferating Microglia, Macrophages/Microglia, Myeloid DCs, Neutrophils, and DC Precursor Cells. Cells in the lymphocyte subset were relabeled according to phenotypes determined by ProjecTILs (version 3.3.0). Cells assigned an “NA” label were presumed to be NK cells, and cells expressing both Cd3e and Klrb1c above the 20th percentile were classified as NKT cells.

Volcano plots were generated using EnhancedVolcano (version 1.2.0). Gene Set Enrichment Analysis (GSEA) was performed using ClusterProfiler (version 4.10.1). Ligand-receptor interaction analysis was conducted with CellChat (version 2.1.2).

T cell V(D)J sequencing data was analyzed using immunarch (version 1.0.0), and unique clonotypes were identified based on paired TRA and TRB gene sequences. By sample, 66 T cells were sequenced in the PBS + Vehicle condition, 15 in the PBS + TMZ condition, 755 in the RRV-RLI + Vehcile condition, and 1,052 in the RRV-RLI + TMZ condition. Immunarch was also used to find matching TCR CDR3 beta sequences in the McPAS database. TCR distance analysis was performed using the tcrdist3 package (v0.2.2) in Jupyter notebook (Python 3.12.7). All single-cell sequencing data analyzed in this study can be accessed from the Gene Expression Omnibus repository, accession code GSE278988.

### Human bulk RNA-sequencing analysis

To evaluate the gene expression of *IL15* across different cancer types, the TCGA PanCancer Atlas from Hoadley et al. was accessed via cBioPortal (cbioportal.org) for *IL15* RSEM scaled estimates in available solid tumor samples^76^. Because no new tissue samples were collected for this study, IRB approval was not needed. To investigate differences according to magnitude of *IL15* expression in glioblastoma, the TCGA cohort of primary glioblastoma samples was stratified into high and low *IL15* expression at the 80th percentile after extracting HTSeq counts via TCGAbiolinks and converting into counts per million (CPM) via edgeR^77^. Differentially expressed genes were evaluated between high and low *IL15* glioblastoma patients via DESeq2 and defined as genes with a Benjamini–Hochberg adjusted *p*-value < 0.05 and a log2Fold Change > 2. Gene ontology enrichment analysis was performed on overexpressed genes in high *IL15* glioblastoma patients to identify the overrepresented biological processes, cellular components, and molecular functions in those tumors. To quantify and compare the immune cell infiltration between high and low *IL15* glioblastoma patients, RSEM-scaled estimates were converted into transcripts per million (TPM) and processed through the CIBERSORT web application (https://cibersortx.stanford.edu/), a deconvolution algorithm that infers the proportion of 22 types of tumor-infiltrating immune cells from bulk RNA-sequencing data^78^. The degree of immune cell infiltration was compared using the Mann-Whitney U test. A two-tailed *p*-value < 0.05 was used as the threshold for statistical significance.

### Human scRNA and immune cell profiling analysis

Differences in transcriptomic expression were investigated by analyzing single-cell RNA sequencing data from four previously published GBM resection specimens^29^. Clusters and cell identities largely mirrored those identified in the original study; however, immune cells were further subdivided to refine cell-type classification. This subclassification of immune cells was achieved using labels suggested by SingleR and marker gene expression identified through literature review. Samples were classified as *IL15* High or *IL15* Low based on standard analysis of *IL15* gene expression levels. Differential gene expression was visualized using volcano plots generated with EnhancedVolcano. Gene ontology analysis was conducted using ToppGene, and cellular communication via ligand-receptor interactions was assessed using CellChat. Samples were collected from the core and edge of tumors. Tumor size was not available for our analyses. We did not differentiate between sample spatial localization for the purpose of our analyses. The alluvial plot in Fig. 1H reports per-sample cell counts normalized to the total number of cells.

### Statistical analysis

Statistical analyses were performed using Prism 10 (GraphPad). Specific study parameters and statistical methods are detailed within the figure legends. Comparisons between two groups were conducted using a two-tailed Student’s t-test. For comparisons among multiple groups, a one-way ANOVA was employed, followed by Fisher’s LSD post-hoc test for pairwise comparisons, assuming a Gaussian distribution and equal standard deviations. If non-equal standard deviations, but Gaussian distribution, a Welch’s t-test was utilized. For non-parametric comparisons, the Mann-Whitney U test or Kruskal-Wallis test with Dunn’s post-hoc test was applied. Kaplan-Meier analysis was used for in vivo survival studies, with differences assessed via the log-rank (Mantel-Cox) test. Outliers were removed based on Grubbs’ test.

### Reporting summary

Further information on research design is available in the Nature Portfolio Reporting Summary linked to this article.

## Supplementary information

Supplementary Information

Reporting Summary

Transparent Peer Review file

## Source data

Source Data

## Data Availability

Single-cell RNA sequencing data generated in this study have been deposited in the GEO repository (GSE278988). We also utilized publicly available data from the TCGA PanCancer Atlas (cBioPortal), CIBERSORTx, the McPAS TCR database, and previously published human GBM scRNA-seq data (GSE84465)^29,76^. Source data are otherwise provided with this paper. GEO repository link for the scRNA sequencing data generated in this study: https://www.ncbi.nlm.nih.gov/geo/query/acc.cgi?acc=GSE278988. Source data are provided with this paper.

## Code availability

No custom code was developed or utilized for this work. All analyses were performed using the software described in Methods.
