# Supplementary material for: Non-lytic viral immunotherapy induces long-term glioblastoma survival and tumor-specific immunity without eliciting an antiviral response
Source: Nat Commun. 2026 May 19;17:6613. doi: 10.1038/s41467-026-72746-5 (PMC13381920; doi:10.1038/s41467-026-72746-5)
Supplement: Supplementary file 1 — Supplementary Information [file 41467_2026_72746_MOESM1_ESM.pdf]

Non-lytic viral immunotherapy induces long-term glioblastoma survival and tumor-specific immunity without eliciting an antiviral response

Alexander F. Haddad,[1] Atul Saha,[1,3] Sara A. Collins,[1,4] Isabella Lovalvo,[1] Sabraj Gill,[1] Megan L. Montoya,[1,5] Poojan Shukla,[1] Jinpyo Hong,[1,6] Elaina Wang,[1,7] Pavlina Chuntova,[1] Meeki Lad,[1] Robert Osorio,[1] Jia-Shu Chen,[1] Melissa Sathavipat,[1] Saket Jain,[1,5] Eric Chalif,[1,8] Noriyuki Kasahara,[1,2] and Manish K. Aghi[1*]

1. Department of Neurological Surgery, University of California, San Francisco, San Francisco, CA, USA
2. Department of Radiation Oncology, University of California, San Francisco, San Francisco, CA, USA
3. Present address: School of Medicine, University of California, San Diego, La Jolla, CA, USA
4. Present address: 4D Molecular Therapeutics, Emeryville, CA, USA
5. Present address: Genentech, South San Francisco, CA, USA
6. Present address: Penn State College of Medicine, Hershey, PA, USA
7. Present address: Department of Neurosurgery, Brown University, Providence, RI, USA
8. Present address: Department of Neurosurgery, Brigham and Women's Hospital, Harvard Medical School, Boston, MA, USA

Supplementary Information

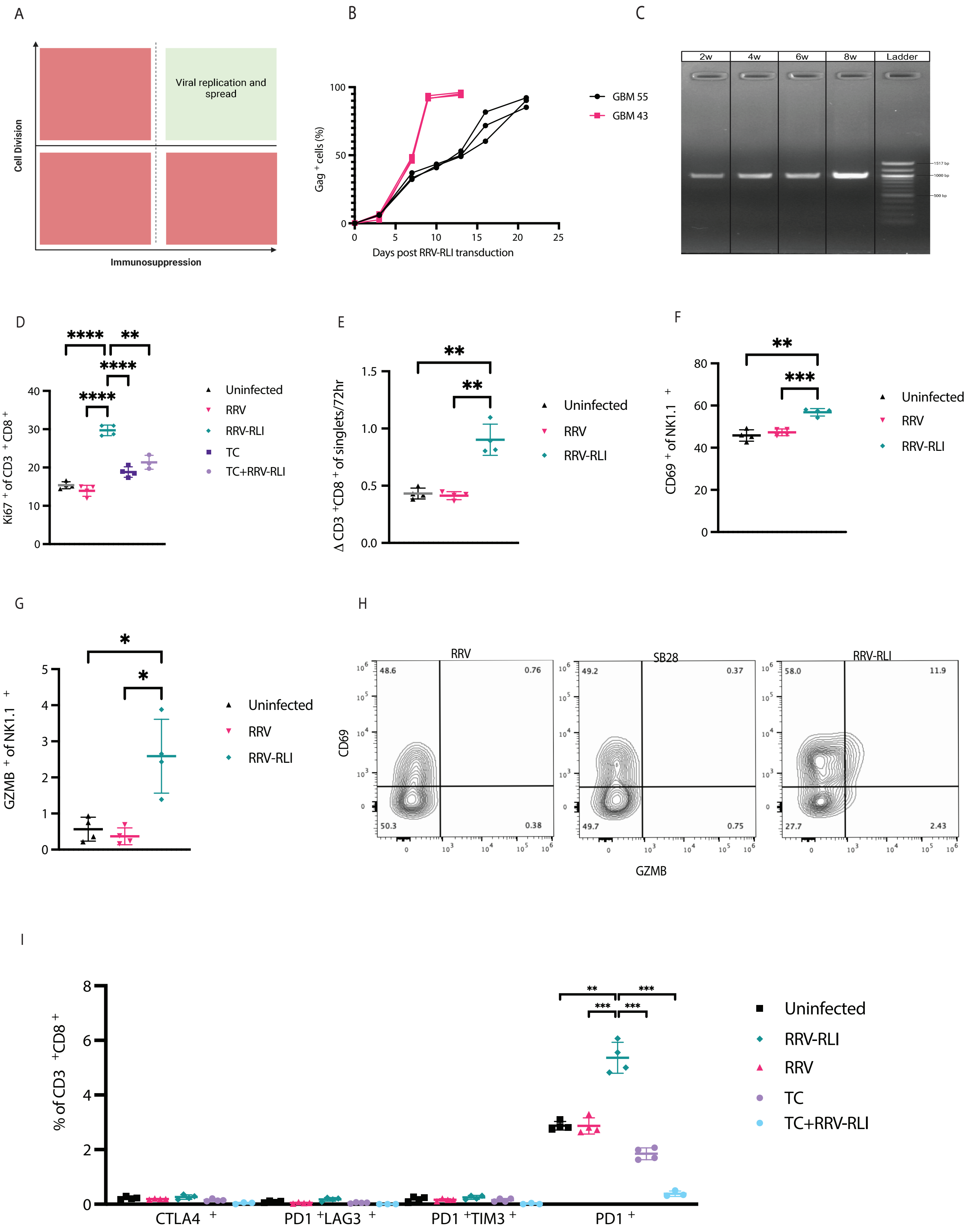

A
Viral replication and spread
Cell Division
Immunosuppression
B
Gag + cells (%)
Days post RRV-RLI transduction
GBM 55
GBM 43
C
2w
4w
6w
8w
Ladder
D
Ki67 + of CD3 + CD8 +
Uninfected
RRV
RRV-RLI
TC
TC+RRV-RLI
E
Δ CD3 +CD8 + of singlets/72hr
Uninfected
RRV
RRV-RLI
F
CD69 + of NK1.1 +
Uninfected
RRV
RRV-RLI
G
GZMB + of NK1.1 +
Uninfected
RRV
RRV-RLI
H
RRV
SB28
RRV-RLI
CD69
GZMB
I
% of CD3 +CD8 +
CTLA4 +
PD1 +LAG3 +
PD1 +TIM3 +
PD1 +
Uninfected
RRV-RLI
RRV
TC
TC+RRV-RLI

Supplementary Fig. 1. RRV-RLI rationale and function *in vitro.* (**A**) RRV-RLI relies on cell division and immunosuppression to form a replication permissive niche. (**B**) RRV-RLI efficiently spreads in human patient derived xenograft cell lines (n=3 for all groups). (**C**) RRV-RLI demonstrates stability and lack of transgene dropout after 8 weeks in culture in the SB28 cell line. (**D**) RRV-RLI infected SB28 tumor cells increase $Ki67^+$ at 48 hours post-addition in co-cultured CD8 T cells relative to RRV and uninfected cells (RRV-RLI vs. Uninfected: p<0.0001; RRV-RLI vs. RRV: p<0.0001; RRV-RLI vs. TC: p<0.0001; RRV-RLI vs. TC+RLI-RRV: p=0.004, Uncorrected two-sided Welch's t-test, n=3 for TC + RRV-RLI, n=4 for all other groups). **(E)** Co-culture of $CD8^+$ T cells with infected SB28 tumor cells demonstrates increased change in $CD8^+$ T cell frequency relative to singlets at 72 hours when compared to RRV infected and uninfected cells (0.90 (RRV-RLI) vs. 0.43 (Uninfected) vs. 0.41 (RRV), RRV-RLI vs. PBS: p=0.004; RRV-RLI vs. RRV: p=0.004, Uncorrected two-sided Welch's t-test, n=4 for all groups). (**F**) RRV-RLI infected SB28 tumor cells increase $CD69^+$ single positive cells at 24 hours post-addition in co-cultured NK cells relative to RRV and uninfected cells (56.8% (RRV-RLI) vs. 45.8% (Uninfected) vs. 47.2% (RRV), p<0.002, RRV-RLI vs. Uninfected: p=0.002; RRV-RLI vs. RRV: p=0.0004, Uncorrected two-sided Welch's t-test, n=4 per group). (**G**) RRV-RLI infected SB28 tumor cells increase $GZMB^+$ single positive cells at 24 hours post-addition in co-cultured NK cells relative to RRV and uninfected cells (2.6% (RRV-RLI) vs. 0.37% (RRV) vs. 0.57% (Uninfected), RRV-RLI vs. Uninfected: p=0.04; RRV-RLI vs. RRV: p=0.04, Uncorrected two-sided Welch's t-test, n=4 per group). (**H**) Example gating schematic for CD69 and GZMB expression in $CD3^-NK1.1^+$ cells in co-culture with infected and uninfected SB28 tumor cells. (**I**) Co-culture with RRV-RLI infected tumor cells leads to a low but significant change in PD1 expression (5.4% (RRV-RLI) vs. 2.9% (RRV) vs. 1.9% (TC) vs. 0.38% (TC+RRV-RLI) vs. 2.9% (Uninfected), RRV-RLI vs. Uninfected: p=0.002; RRV-RLI vs. RRV: p=0.0009; RRV-RLI vs. TC: p=0.0004; RRV-RLI vs. TC+RLI-RRV: p=0.0003, Uncorrected two-sided Welch's t-test, n=3 for TC+RRV-RLI, n=4 for all other groups). Data represent biological replicates. **Supplementary Fig. 1B** shows individual replicates. All other graphs show mean +/- SD. *p<0.05; **p<0.01; ***p<0.001; ****p<0.0001.

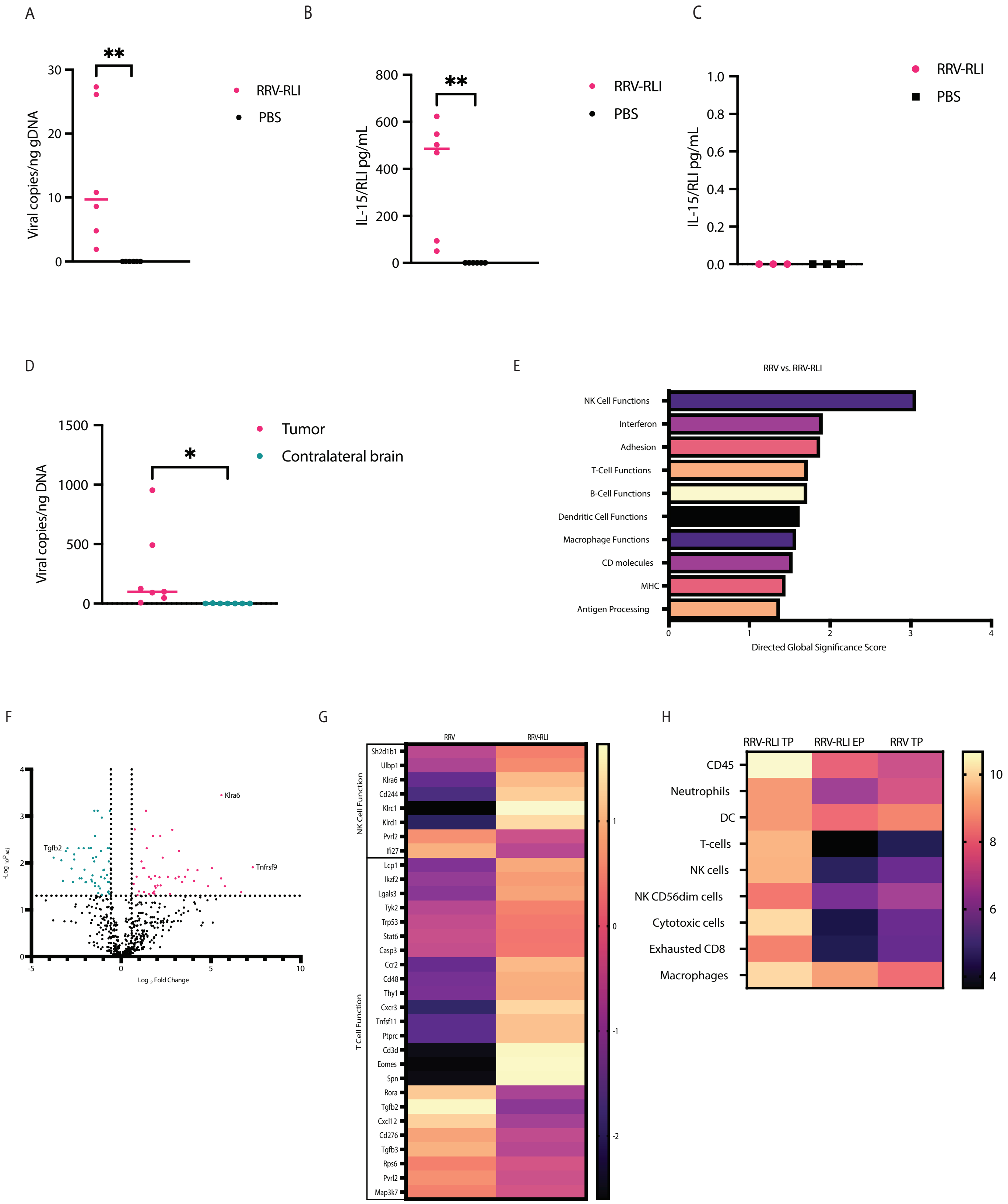
A
**
Viral copies/ng gDNA
30
20
10
0
RRV-RLI
PBS
B
**
IL-15/RLI pg/mL
800
600
400
200
0
RRV-RLI
PBS
C
IL-15/RLI pg/mL
1.0
0.8
0.6
0.4
0.2
0.0
RRV-RLI
PBS
D
*
Viral copies/ng DNA
1500
1000
500
0
Tumor
Contralateral brain
E
RRV vs. RRV-RLI
NK Cell Functions
Interferon
Adhesion
T-Cell Functions
B-Cell Functions
Dendritic Cell Functions
Macrophage Functions
CD molecules
MHC
Antigen Processing
0
1
2
3
4
Directed Global Significance Score
F
Klra6
Tgfb2
Tnfrsf9
-Log 10 P adj
Log 2 Fold Change
-5
0
5
10
G
RRV
RRV-RLI
NK Cell Function
Sh2d1b1
Ulbp1
Klra6
Cd244
Klrc1
Klrd1
Pvrl2
Ifi27
T Cell Function
Lcp1
Ikzf2
Lgals3
Tyk2
Trp53
Stat6
Casp3
Ccr2
Cd48
Thy1
Cxcr3
Tnfsf11
Ptprc
Cd3d
Eomes
Spn
Rora
Tgfb2
Cxcl12
Cd276
Tgfb3
Rps6
Pvrl2
Map3k7
1
0
-1
-2
H
RRV-RLI TP
RRV-RLI EP
RRV TP
CD45
Neutrophils
DC
T-cells
NK cells
NK CD56dim cells
Cytotoxic cells
Exhausted CD8
Macrophages
10
8
6
4

Supplementary Fig. 2. Local RRV-RLI therapy modulates immune response by affecting T cells, NK cells, and antigen presentation. (**A**) Viral copies of RRV-RLI 10 days after virus injection ($p=0.002$, Mann-Whitney U test, n=6 per group). (**B**) Intratumoral IL-15 protein 10 days after virus injection ($p=0.002$, Mann-Whitney U test, n=6 per group). (**C**) No evidence of systemic IL-15/RLI is present in the blood of treated SB28 tumor bearing mice at day 14 after tumor implantation (day 10 after virus injection, n=3 per group). (**D**) Intratumoral viral copies of RRV-RLI at tumor endpoint relative to contralateral brain (mean copies 259 vs. 0.57, $p=0.0005$, Mann-Whitney U test, n=7 per group). (**E**) Directed global significance scores of the top 10 upregulated gene pathways in RRV-RLI treatment vs. RRV treatment. (**F**) Volcano plot showing differentially expressed genes between RRV-RLI and RRV treatment groups at day 14 post tumor implantation timepoint (DESeq2, Benjamini-Hochberg adjusted $p < 0.05$ and log2FC > 0.5). (**G**) Heat map detailing differential gene expression for genes related to T cell function and NK cell function between RRV-RLI and RRV treatment groups. (**H**) Heat map demonstrating calculated immune infiltration scores between RRV-RLI at day 14 post tumor implantation timepoint (RRV-RLI TP), RRV-RLI at endpoint (RRV-RLI EP), and RRV at day 14 post tumor implantation timepoint (RRV TP). For nanostring analysis RRV-RLI n=3, RRV n=2, RRV-RLI EP n=2. **Supplementary Fig. 2A-D** demonstrate a line at the median and individual values. Data represent biological replicates. *$p<0.05$; **$p<0.01$; ***$p<0.001$; ****$p<0.0001$.

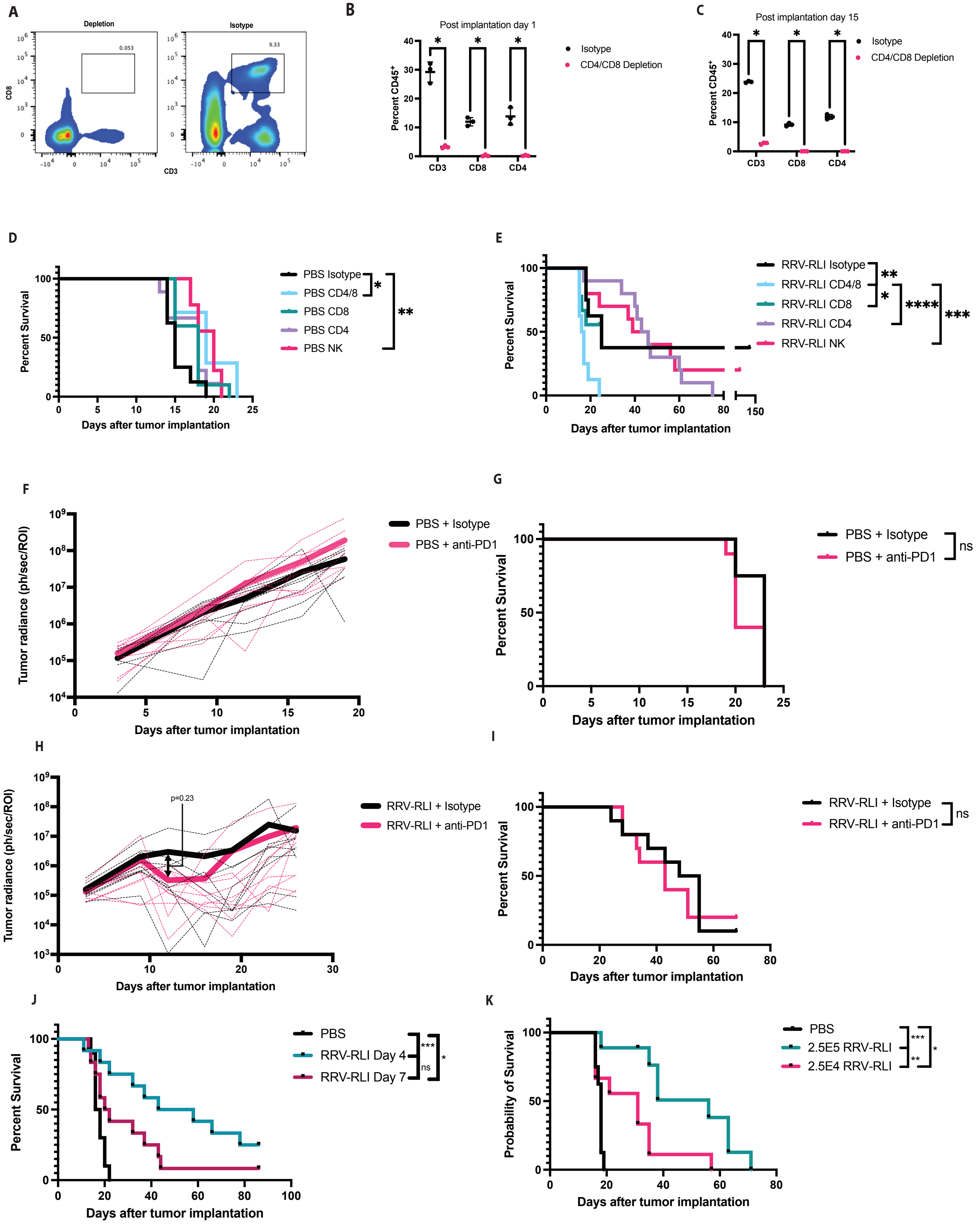

A
Depletion
Isotype
0.053
9.33
CD8
CD3
B
Post implantation day 1
Percent CD45+
CD3
CD8
CD4
Isotype
CD4/CD8 Depletion
C
Post implantation day 15
D
Percent Survival
Days after tumor implantation
PBS Isotype
PBS CD4/8
PBS CD8
PBS CD4
PBS NK
E
RRV-RLI Isotype
RRV-RLI CD4/8
RRV-RLI CD8
RRV-RLI CD4
RRV-RLI NK
F
Tumor radiance (ph/sec/ROI)
PBS + Isotype
PBS + anti-PD1
G
ns
H
p=0.23
RRV-RLI + Isotype
RRV-RLI + anti-PD1
I
J
PBS
RRV-RLI Day 4
RRV-RLI Day 7
K
Probability of Survival
2.5E5 RRV-RLI
2.5E4 RRV-RLI

Supplementary Fig. 3. Validation of immune cell depletion and demonstration that RRV-RLI combination with anti-PD1 treatment does not alter therapeutic efficacy.
(**A**) Example flow cytometry gating for the validation of CD8 depletion studies.(**B**) Flow cytometric analysis of anti-CD4 and anti-CD8 antibody-treated SB28 tumor bearing mice reveals successful depletion of $CD3^+$ (3.2% vs. 29.2%, p=0.005, Uncorrected two-sided Welch's t-test), $CD8^+$ (0.2% vs. 12.0%, p=0.004, Uncorrected two-sided Welch's t-test), and $CD4^+$ T cells (0.23% vs. 13.8%, p=0.02, Uncorrected two-sided Welch's t-test, n=3 per group), relative to isotype control at day 1 post tumor implantation. (**C**) Similar results were seen for $CD3^+$ (2.7% vs. 23.8%, p<0.0001, Uncorrected two-sided Welch's t-test), $CD8^+$ (0.04% vs. 9.1%, p=0.001, Uncorrected two-sided Welch's t-test), and $CD4^+$ T cells (0.03% vs. 11.8%, p=0.002, Uncorrected two-sided Welch's t-test, n=3 per group), relative to isotype control at day 15 post tumor implantation. (**D**) Comparison of all PBS treatment groups across depletion types demonstrates small differences in survival between Isotype vs. CD4/8 (p=0.01, Isotype: n=8, CD4/8: n=7) and Isotype vs. NK depletion (p=0.001 Isotype: n=8, NK: n=9) (Log-Rank Mantel-Cox test). (**E**) Comparison of all RRV-RLI treatment groups across depletion types demonstrates differences in survival between all groups and CD4/8 depletion (Isotype (n=8) vs. CD4/8 (n=8): p= 0.002; CD4/8 vs. CD8 (n=9): p=0.03; CD4/8 vs. CD4 (n=10): p<0.0001; CD4/8 vs. NK (n=10): p=0.0002, Log-Rank Mantel-Cox test). There were no significant differences in survival between other depletion groups. (**F**) In PBS-treated mice bearing SB28 tumors, anti-PD1 therapy did not reduce tumor growth on bioluminescent imaging compared to isotype-treated controls. (**G**) In PBS-treated mice bearing SB28 tumors, anti-PD1 therapy did not extend survival compared to isotype-treated controls (median survival 20 days vs. 23 days; p =0.13, Log-Rank Mantel-Cox test, Isotype n=8, anti-PD1 n=10). (**H**) In RRV-RLI-treated mice bearing SB28 tumors, anti-PD1 therapy did not reduce tumor growth on bioluminescent imaging compared to isotype-treated controls (p=0.23, day 12 post tumor implantation, Uncorrected two-sided Welch's t-test) (**I**) In RRV-RLI-treated mice bearing SB28 tumors, anti-PD1 therapy did not extend survival compared to isotype-treated controls (median survival 43 days vs. 51 days; p =0.70, Log-Rank Mantel-Cox test, n=10 for both groups). (**J**) Timing of RRV-RLI treatment in SB28 tumor bearing mice affects survival (median survival 51 days vs. 21 days vs. 17 days; Day 4 (n=12) vs. Day 7 (n=12): p= 0.06; PBS (n=10) vs. Day 4: p=0.0002; PBS vs. Day 7 p=0.04, Log-Rank Mantel-Cox test). (**K**) RRV-RLI treatment dose in SB28 tumor bearing mice significantly affects survival (median survival 56 days vs. 31 days vs. 18 days, 2E5 (n=9) vs. 2E4 (n=9): p= 0.008; PBS (n=8) vs. 2E5: p=0.0002; PBS vs. 2E4 p=0.02, Log-Rank Mantel-Cox test). Data represent biological replicates. *p<0.05; **p<0.01; ***p<0.001; ****p<0.0001.

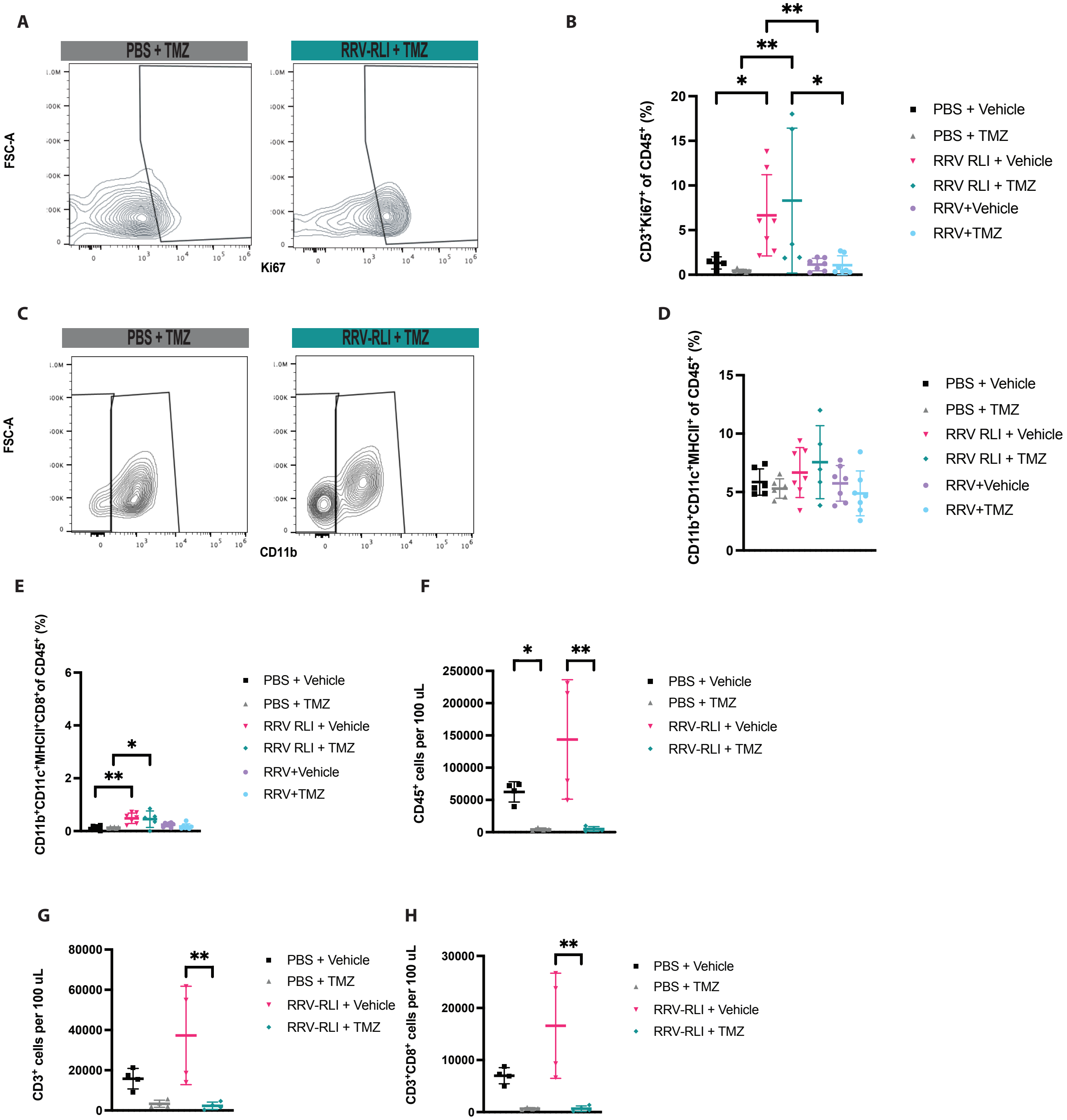

A
PBS + TMZ
RRV-RLI + TMZ
FSC-A
Ki67
B
CD3+Ki67+ of CD45+ (%)
PBS + Vehicle
PBS + TMZ
RRV RLI + Vehicle
RRV RLI + TMZ
RRV+Vehicle
RRV+TMZ
C
PBS + TMZ
RRV-RLI + TMZ
FSC-A
CD11b
D
CD11b+CD11c+MHCII+ of CD45+ (%)
PBS + Vehicle
PBS + TMZ
RRV RLI + Vehicle
RRV RLI + TMZ
RRV+Vehicle
RRV+TMZ
E
CD11b+CD11c+MHCII+CD8+of CD45+ (%)
PBS + Vehicle
PBS + TMZ
RRV RLI + Vehicle
RRV RLI + TMZ
RRV+Vehicle
RRV+TMZ
F
CD45+ cells per 100 uL
PBS + Vehicle
PBS + TMZ
RRV-RLI + Vehicle
RRV-RLI + TMZ
G
CD3+ cells per 100 uL
PBS + Vehicle
PBS + TMZ
RRV-RLI + Vehicle
RRV-RLI + TMZ
H
CD3+CD8+ cells per 100 uL
PBS + Vehicle
PBS + TMZ
RRV-RLI + Vehicle
RRV-RLI + TMZ

Supplementary Fig. 4. Additional immune phenotyping reveals systemic TMZ associated myelosuppression. (**A**) Example gating strategy for $CD3^+Ki67^+$ cells (already gated on live $CD45^+CD11b^-$ $CD3^+$). (**B**) Percent tumor infiltrating $CD3^+Ki67^+$ cells with significant increases in RRV-RLI vs. associated PBS and RRV treatment groups (RRV-RLI + Vehicle vs. PBS + Vehicle: p=0.02; RRV-RLI + TMZ vs. PBS + TMZ: p=0.002; RRV-RLI + Vehicle vs. RRV + Vehicle: p=0.004; RRV-RLI + TMZ vs. RRV + TMZ: p=0.01, Uncorrected Dunn's test). (**C**) Example gating strategy for $CD11b^+$ cells (already gated on live $CD45^+$). (**D**) Percent tumor infiltrating $CD11b^+CD11c^+MHCII^+$ cells without a significant difference between treatment groups (p>0.05 for all comparisons, Uncorrected Dunn's test). (**E**) Percent tumor infiltrating $CD11b^+CD11c^+MHCII^+CD8^+$ cells in the various treatment groups with a slight increase in RRV-RLI vs. PBS treatment groups (RRV-RLI + Vehicle vs. PBS + Vehicle: p=0.001; RRV-RLI + TMZ vs. PBS + TMZ: p=0.02, Uncorrected Dunn's). No significant difference between PBS and RRV treatment groups (p>0.05 all comparisons, Uncorrected Dunn's). (**F**) $CD45^+$ counts per 100 uL in SB28 tumor bearing mice blood samples with reductions in total cells per 100 uL in PBS + TMZ vs. PBS + Vehicle (p=0.045, Uncorrected Dunn's test) and RRV-RLI + TMZ vs. RRV-RLI + Vehicle (p=0.006, Uncorrected Dunn's test) (**G**) $CD3^+$ counts per 100 uL in SB28 tumor bearing mice blood samples with a reduction in total cells per 100 uL in RRV-RLI + TMZ vs. RRV-RLI + Vehicle (p=0.004, Uncorrected Dunn's test) (**H**) $CD3^+CD8^+$ counts per 100 uL in SB28 tumor bearing mice blood samples with a reduction in total cells per 100 uL in RRV-RLI + TMZ vs. RRV-RLI + Vehicle (p=0.005, Uncorrected Dunn's test). *p<0.05; **p<0.01; ***p<0.001; ****p<0.0001. For brain flow cytometry data: n=6 for PBS + Vehicle and PBS + TMZ groups, n=7 for RRV-RLI + Vehicle and RRV + Vehicle and RRV + TMZ, n=5 for RRV-RLI + TMZ. N=4 per group for blood flow cytometry data. Graphs show mean +/- SD.

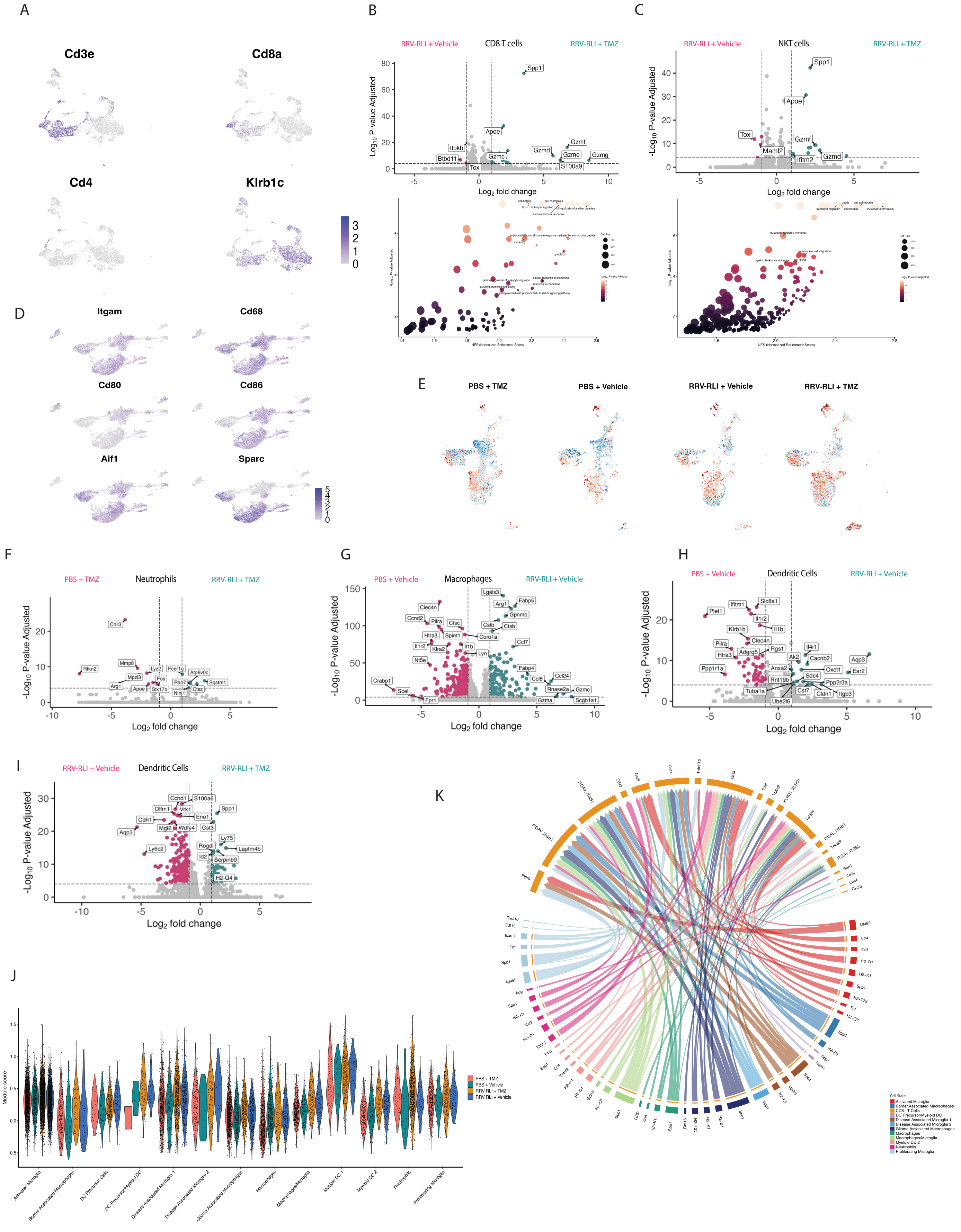

A
Cd3e
Cd8a
Cd4
Klrb1c
B
RRV-RLI + Vehicle
CD8 T cells
RRV-RLI + TMZ
-Log10 P-value Adjusted
Log2 fold change
Spp1
Apoe
Itpkb
Btbd11
Tox
Gzmc
Gzmd
Gzmf
Gzme
Gzmg
S100a9
NES (Normalized Enrichment Score)
C
RRV-RLI + Vehicle
NKT cells
RRV-RLI + TMZ
Spp1
Apoe
Tox
Maml2
Gzmf
Ifitm2
Gzmd
D
Itgam
Cd68
Cd80
Cd86
Aif1
Sparc
E
PBS + TMZ
PBS + Vehicle
RRV-RLI + Vehicle
RRV-RLI + TMZ
F
PBS + TMZ
Neutrophils
RRV-RLI + TMZ
Chil3
Rtkn2
Mmp8
Mpzl3
Arg1
Apoe
Lyz2
Fos
Stk17b
Fcer1g
Rab7
Ninj1
Atp6v0c
Ctsz
Sqstm1
G
PBS + Vehicle
Macrophages
RRV-RLI + Vehicle
Clec4n
Ccnd2
Pilra
Htra3
Klra2
Il1r2
Nt5e
Crabp1
Scel
Fpr1
Ctsc
Spint1
Il1b
Lyn
Lgals3
Fabp5
Arg1
Gpnmb
Cstb
Ctsb
Coro1a
Ccl7
Fabp4
Ccl24
Ccl8
Rnase2a
Gzmc
Gzma
Scgb1a1
H
PBS + Vehicle
Dendritic Cells
RRV-RLI + Vehicle
Plet1
Ifitm1
Slc8a1
Il1r2
Il1b
Klrb1b
Clec4n
Pilra
Htra3
Adgrg5
Rgs1
Ppp1r1a
Il4i1
Ak2
Cacnb2
Aqp3
Ear2
Anxa2
Oxct1
Rnf19b
Sdc4
Ppp2r3a
Tuba1a
Cst7
Cldn1
Itgb3
Ube2i6
I
RRV-RLI + Vehicle
Dendritic Cells
RRV-RLI + TMZ
Ccnd1
S100a6
Olfm1
Vrk1
Spp1
Cdh1
Eno1
Aqp3
Mgl2
Wdfy4
Cst3
Ly75
Ly6c2
Rogdi
Laptm4b
Id2
Serpinb9
H2-Q4
J
Module score
PBS + TMZ
PBS + Vehicle
RRV RLI + TMZ
RRV RLI + Vehicle
Activated Microglia
Border Associated Macrophages
DC Precursor Cells
DC Precursor/Myeloid DC
Disease Associated Microglia 1
Disease Associated Microglia 2
Glioma Associated Macrophages
Macrophages
Macrophages/Microglia
Myeloid DC 1
Myeloid DC 2
Neutrophils
Proliferating Microglia
K
Cell State
Activated Microglia
Border Associated Macrophages
CD8+ T Cells
DC Precursor/Myeloid DC
Disease Associated Microglia 1
Disease Associated Microglia 2
Glioma Associated Macrophages
Macrophages
Macrophages/Microglia
Myeloid DC 2
Neutrophils
Proliferating Microglia

Supplementary Fig. 5. Single-cell RNA sequencing identifies enhanced effector cell activation and antigen presentation driving efficacy in combined RRV-RLI and TMZ therapy. (**A**) UMAP of all sequenced T and NK cells demonstrating key marker gene expression. (**B**) Volcano plot demonstrating CD8 T cell differential gene expression in RRV-RLI + TMZ vs. RRV-RLI + Vehicle with additional GSEA. (**C**) Volcano plot demonstrating NKT cell differential gene expression in RRV-RLI + TMZ vs. RRV-RLI + Vehicle with additional GSEA. (**D**) UMAP of all sequenced myeloid cells demonstrating key marker gene expression. (**E**) UMAP of all sequenced myeloid cells demonstrating changes in MHC class I gene expression score between samples. (**F**) Volcano plot demonstrating neutrophil differential gene expression in PBS + TMZ vs. RRV-RLI + TMZ. (**G**) Volcano plot demonstrating macrophage differential gene expression in PBS + Vehicle vs. RRV-RLI + Vehicle. (**H**) Volcano plot demonstrating dendritic cell differential gene expression in PBS + Vehicle vs. RRV-RLI + Vehicle. (**I**) Volcano plot demonstrating dendritic cell differential gene expression in RRV-RLI + Vehicle vs. RRV-RLI + TMZ (**J**) Violin plot of MHC class I gene expression score in myeloid subpopulations between samples. (**K**) Cell chat analysis of incoming signaling in RRV-RLI + TMZ vs. RRV-RLI + Vehicle. *p<0.05; **p<0.01; ***p<0.001; ****p<0.0001. Data represent FACS-sorted $CD45^+$ cells from a mouse per condition. Volcano plots made with DESeq2, Benjamini-Hochberg adjusted $p < 1x10^4$ and log2FC > 0.95. See data availability for GEO information.

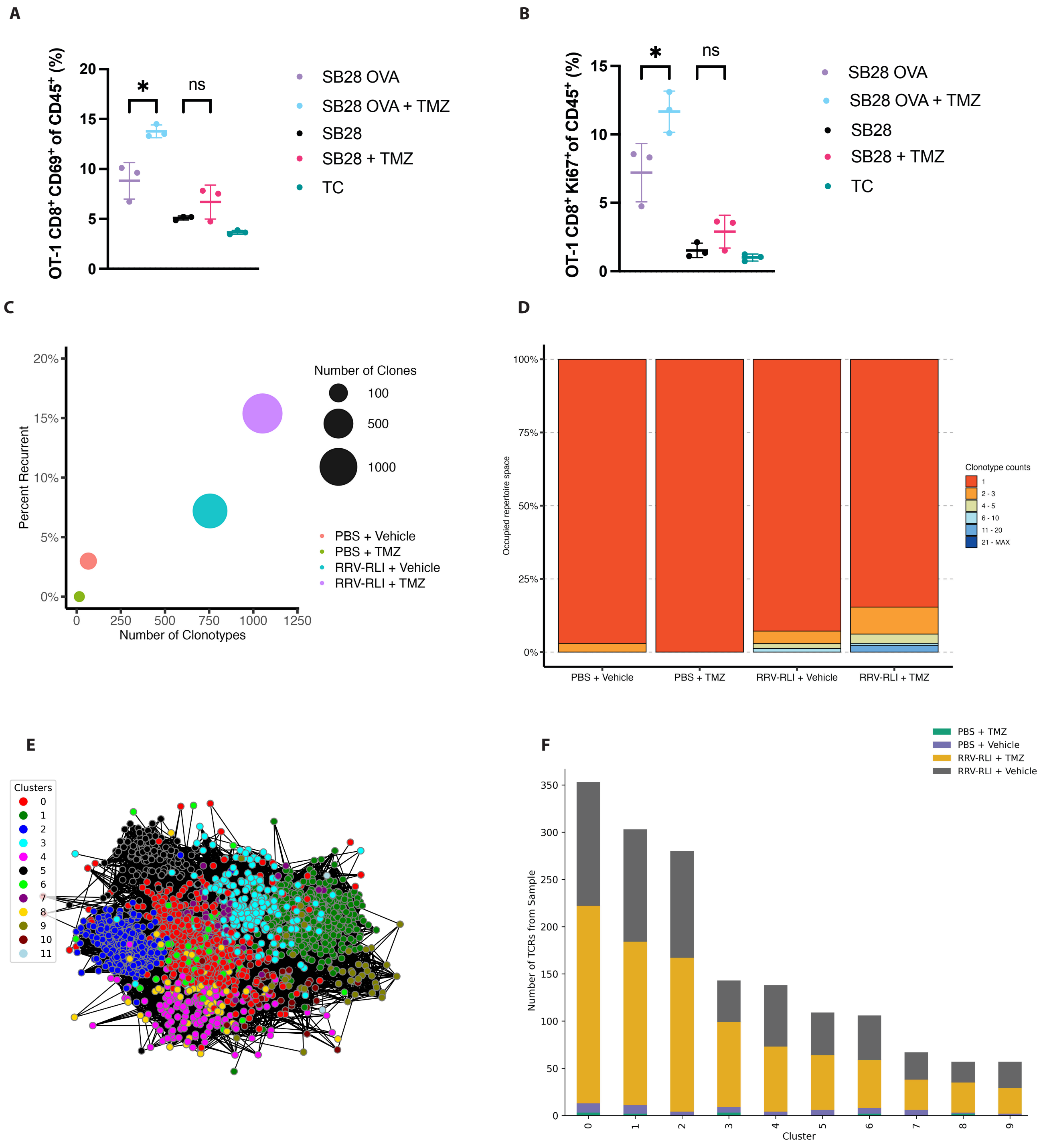

A
OT-1 CD8+ CD69+ of CD45+ (%)
*
ns
SB28 OVA
SB28 OVA + TMZ
SB28
SB28 + TMZ
TC
B
OT-1 CD8+ Ki67+ of CD45+ (%)
*
ns
SB28 OVA
SB28 OVA + TMZ
SB28
SB28 + TMZ
TC
C
Percent Recurrent
Number of Clonotypes
Number of Clones
100
500
1000
PBS + Vehicle
PBS + TMZ
RRV-RLI + Vehicle
RRV-RLI + TMZ
D
Occupied repertoire space
Clonotype counts
1
2 - 3
4 - 5
6 - 10
11 - 20
21 - MAX
PBS + Vehicle
PBS + TMZ
RRV-RLI + Vehicle
RRV-RLI + TMZ
E
Clusters
0
1
2
3
4
5
6
7
8
9
10
11
F
PBS + TMZ
PBS + Vehicle
RRV-RLI + TMZ
RRV-RLI + Vehicle
Number of TCRs from Sample
Cluster

Supplementary Fig. 6. Single-cell TCR sequencing suggests tumor-specific T cell clonal expansion induced by RRV-RLI and temozolomide therapy. (**A**) Flow cytometric analysis of in vitro OT-1 T cell priming experiment with increased $CD69^+$ CD8 T cell populations in OVA SB28 cell line treated with TMZ (13.8% vs. 8.8%, p=0.03, Uncorrected two-sided Welch's t-test) no significant difference observed between wildtype SB28 vs. SB28 + TMZ (5.1% vs. 6.7%, p=0.24, Uncorrected two-sided Welch's t-test). (**B**) Flow cytometric analysis of in vitro OT-1 T cell priming experiment with increased $Ki67^+$ CD8 T cell populations in OVA SB28 cell line treated with TMZ (11.7% vs. 7.2%, p=0.047, Uncorrected two-sided Welch's t-test) no significant difference observed between wildtype SB28 vs. SB28 + TMZ (1.5% vs. 2.9%, p=0.17, Uncorrected two-sided Welch's t-test). (**C**) X-Y graph showing increased recurrent T cell clones in RRV-RLI + TMZ vs. RRV-RLI + Vehicle. (**D**) Visualization of clone frequency occupancy by clone rank (**E**) Beta chain clustering of TCRs from all samples within 80 distance units with specific clusters labeled. (**F**) Stacked bar graph demonstrating sample identification across the top 9 beta chain clusters. For flow cytometry data graphs show mean +/- SD and n=3 per group. *p<0.05; **p<0.01; ***p<0.001; ****p<0.0001. Single cell RNA sequencing and TCR data represent FACS-sorted $CD45^+$ cells from a mouse per condition. See data availability for GEO information.

Reagent	Source	Identifier
Anti-mouse FOXP3 BV421 (Clone MF-14)	Biolegend	126419
Zombie Aqua	Biolegend	423101
Anti-mouse Ki67 BV605 (Clone 16A8)	Biolegend	652413
Anti-mouse CD8 BV650 (Clone 53-6.7)	Biolegend	100741
Anti-mouse CD11c BV711 (Clone N418)	Biolegend	117349
Anti-mouse NK 1.1 BV785 (Clone PK136)	Biolegend	108749
Anti-mouse CD11b AF488 (Clone M1/70)	Biolegend	101219
Anti-mouse CD45 PerCP Cy5.5 (Clone 30-F11)	Biolegend	103131
Anti-mouse CD25 PE (Clone PC61)	Biolegend	102007
Anti-mouse CTLA4 PE/Dazzle594 (Clone UC10-4B9)	Biolegend	106317
Anti-mouse F4/80 PE-Cy7 (Clone BM8)	Biolegend	123113
Anti-mouse CD3 AF647 (Clone 17A2)	Biolegend	100209
Anti-mouse MHC II AF700 (Clone M5/114.15.2)	Biolegend	107621
Anti-mouse CD4 APC/Fire750 (Clone GK1.5)	Biolegend	100459
Anti-mouse CD45 BV510 (Clone 30-F11)	Biolegend	103137
Anti-mouse PD-1 BV605 (Clone 29F.1A12)	Biolegend	135219
Anti-mouse TIM-3 BV711 (Clone B8.2C12)	Biolegend	134021
Anti-mouse LAG-3 BV711 (Clone C9B7W)	Biolegend	125219

Anti-mouse CD4 AF488 (Clone GK1.5)	Biolegend	100425
Anti-mouse CD3 PE (Clone 17A2)	Biolegend	100205
Zombie Red	Biolegend	423109
Anti-mouse CTLA4 PE Cy7 (Clone UC10-4B9)	Biolegend	106313
Anti-mouse CD8 APC Cy7 (Clone 53-6.7)	Biolegend	100713
Anti-mouse IFN-gamma (Clone XMG1.2)	Biolegend	505829
Anti-mouse PD-1 BV650 (Clone 29F.1A12)	Biolegend	135243
Anti-mouse CD45 AF488 Cy5.5 (Clone 30-F11)	Biolegend	103121
Anti-mouse CD69 PE Cy7 (Clone H1.2F3)	Biolegend	104511
Anti-mouse GZMB AF647 (Clone GB11)	Biolegend	515405
Anti-mouse TNF-a (Clone MP6-XT22)	Biolegend	506338

Supplementary Table 1. Antibodies used for flow cytometry. List of antibodies used in this study including target, fluorophore, clone, identifier, and manufacturer.
