# Supplementary material for: Non-lytic viral immunotherapy induces long-term glioblastoma survival and tumor-specific immunity without eliciting an antiviral response
Source: Nat Commun. 2026 May 19;17:6613. doi: 10.1038/s41467-026-72746-5 (PMC13381920; doi:10.1038/s41467-026-72746-5)
Supplement: Supplementary file 2 — Reporting Summary [file 41467_2026_72746_MOESM2_ESM.pdf]

Corresponding author(s): Manish Aghi, MD PhD

Last updated by author(s): Dec 17, 2025

Reporting Summary

Nature Portfolio wishes to improve the reproducibility of the work that we publish. This form provides structure for consistency and transparency in reporting. For further information on Nature Portfolio policies, see our Editorial Policies and the Editorial Policy Checklist.

Statistics

For all statistical analyses, confirm that the following items are present in the figure legend, table legend, main text, or Methods section.

n/a	Confirmed	
☐	☒	The exact sample size (*n*) for each experimental group/condition, given as a discrete number and unit of measurement
☐	☒	A statement on whether measurements were taken from distinct samples or whether the same sample was measured repeatedly
☐	☒	The statistical test(s) used AND whether they are one- or two-sided<br>*Only common tests should be described solely by name; describe more complex techniques in the Methods section.*
☐	☒	A description of all covariates tested
☐	☒	A description of any assumptions or corrections, such as tests of normality and adjustment for multiple comparisons
☐	☒	A full description of the statistical parameters including central tendency (e.g. means) or other basic estimates (e.g. regression coefficient) AND variation (e.g. standard deviation) or associated estimates of uncertainty (e.g. confidence intervals)
☐	☒	For null hypothesis testing, the test statistic (e.g. *F*, *t*, *r*) with confidence intervals, effect sizes, degrees of freedom and *P* value noted<br>*Give P values as exact values whenever suitable.*
☐	☒	For Bayesian analysis, information on the choice of priors and Markov chain Monte Carlo settings
☐	☒	For hierarchical and complex designs, identification of the appropriate level for tests and full reporting of outcomes
☒	☐	Estimates of effect sizes (e.g. Cohen's *d*, Pearson's *r*), indicating how they were calculated

Our web collection on statistics for biologists contains articles on many of the points above.

Software and code

Policy information about availability of computer code

Data collection	Flow cytometry: Attune NxT software<br><br>BLI: Living Image software<br><br>scRNA-seq: 10x Genomics Chromium software and Illumina control software<br><br>NanoString: nCounter SPRINT Profiler software
Data analysis	Mouse Nanostring- Raw files were analyzed on the Rosalind online platform (OnRamp Bio) to calculate normalized gene expression counts, determine significance of differentially expressed genes, and derive cell type scores. Differential gene expression analysis was performed using the DESeq2 package in R.<br><br>Mouse scRNA- Volcano plots were generated using EnhancedVolcano (version 1.2.0). Gene Set Enrichment Analysis (GSEA) was performed using ClusterProfiler (version 4.10.1). Ligand-receptor interaction analysis was conducted with CellChat (version 2.1.2). Alluvial bar graphs showing sample composition by cell type were created using ggalluvial (version 0.12.5). T cell V(D)J sequencing data was analyzed using immunarch (version 1.0.0).<br><br>Human scRNA analysis- SingleR and marker gene expression identified through literature review. Samples were classified as IL15 High or IL15 Low based on standard analysis of IL15 gene expression levels. Differential gene expression was visualized using volcano plots generated with EnhancedVolcano. Gene ontology analysis was conducted using ToppGene, and cellular communication via ligand-receptor interactions was assessed using CellChat.

Human bulk RNA sequencing analysis- To investigate differences according to magnitude of IL15 expression in glioblastoma, the TCGA cohort of primary glioblastoma samples was stratified into high and low IL15 expression at the 80th percentile after extracting HTSeq counts via TCGAbiolinks and converting into counts per million (CPM) via edgeR.84 Differentially expressed genes were evaluated between high and low IL15 glioblastoma patients via DESeq2 and defined as genes with a Benjamini–Hochberg adjusted p-value < 0.05 and a log2Fold Change > 2. Gene ontology enrichment analysis was performed on overexpressed genes in high IL15 glioblastoma patients to identify the overrepresented biological processes, cellular components, and molecular functions in those tumors. To quantify and compare the immune cell infiltration between high and low IL15 glioblastoma patients, RSEM scaled estimates were converted into transcripts per million (TPM) and processed through the CIBERSORT web application (https://cibersortx.stanford.edu/), a deconvolution algorithm that infers the proportion of 22 types of tumor-infiltrating immune cells from bulk RNA-sequencing data.85 Degree of immune cell infiltration was compared using the Mann-Whitney U test. A two-tailed p-value < 0.05 was used as the threshold for statistical significance

For manuscripts utilizing custom algorithms or software that are central to the research but not yet described in published literature, software must be made available to editors and reviewers. We strongly encourage code deposition in a community repository (e.g. GitHub). See the Nature Portfolio guidelines for submitting code & software for further information.

Data

Policy information about availability of data

All manuscripts must include a data availability statement. This statement should provide the following information, where applicable:

- Accession codes, unique identifiers, or web links for publicly available datasets
- A description of any restrictions on data availability
- For clinical datasets or third party data, please ensure that the statement adheres to our policy

All mouse single cell sequencing data analyzed in this study can be accessed from the Gene Expression Omnibus repository, accession code GSE278988.

Research involving human participants, their data, or biological material

Policy information about studies with human participants or human data. See also policy information about sex, gender (identity/presentation), and sexual orientation and race, ethnicity and racism.

Reporting on sex and gender	Human data utilized in this study was from publicly available sources or datasets. No new human data was collected.
Reporting on race, ethnicity, or other socially relevant groupings	Human data utilized in this study was from publicly available sources or datasets. No new human data was collected.
Population characteristics	Human data utilized in this study was from publicly available sources or datasets. No new human data was collected.
Recruitment	Human data utilized in this study was from publicly available sources or datasets. No new human data was collected.
Ethics oversight	Human data utilized in this study was from publicly available sources or datasets. No new human data was collected.

Note that full information on the approval of the study protocol must also be provided in the manuscript.

Field-specific reporting

Please select the one below that is the best fit for your research. If you are not sure, read the appropriate sections before making your selection.

☒ Life sciences ☐ Behavioural & social sciences ☐ Ecological, evolutionary & environmental sciences

For a reference copy of the document with all sections, see nature.com/documents/nr-reporting-summary-flat.pdf

Life sciences study design

All studies must disclose on these points even when the disclosure is negative.

Sample size	For in vivo experiments, 8-12 week-old mice were utilized, with 6 to 11 mice per group, ensuring adequate statistical power based on prior studies in intracranial glioblastoma models, our prior experiences, and effect sizes observed in preliminary experiments. For in vitro experiments, a minimum of 3 biological replicates per condition was utilized.
Data exclusions	For immunophenotyping experiments, outliers were removed based on Grubbs' test. For survival experiments, mice were censored at time of death if they reached a non-tumor related endpoint. Censored mice are shown on the KM plots as indicated by hashmarks.
Replication	Key in vivo findings were reproduced in at least two independent experiments. In particular, the survival benefit generated by in vivo administration of RRV-RLI has been reproduced in experiments spanning multiple years and virus preps.
Randomization	Mice were randomized to treatment arms following baseline bioluminescence imaging to ensure comparable starting tumor burden across groups.

Blinding

Blinding was not feasible due to the nature of the experimental procedures. However, primary outcome measures were objective and quantitative.

Reporting for specific materials, systems and methods

We require information from authors about some types of materials, experimental systems and methods used in many studies. Here, indicate whether each material, system or method listed is relevant to your study. If you are not sure if a list item applies to your research, read the appropriate section before selecting a response.

Materials & experimental systems

n/a	Involved in the study	
☐	☒	Antibodies
☐	☒	Eukaryotic cell lines
☒	☐	Palaeontology and archaeology
☐	☒	Animals and other organisms
☐	☒	Clinical data
☒	☐	Dual use research of concern
☒	☐	Plants

Methods

n/a	Involved in the study	
☒	☐	ChIP-seq
☐	☒	Flow cytometry
☒	☐	MRI-based neuroimaging

Antibodies

Antibodies used

Anti-mouse FOXP3 BV421 (Clone
MF-14) Biolegend 126419
Zombie Aqua Biolegend 423101
Anti-mouse Ki67 BV605 (Clone
16A8) Biolegend 652413
Anti-mouse CD8 BV650 (Clone
53-6.7) Biolegend 100741
Anti-mouse CD11c BV711 (Clone
N418) Biolegend 117349
Anti-mouse NK 1.1 BV785 (Clone
PK136) Biolegend 108749
Anti-mouse CD11b AF488 (Clone
M1/70) Biolegend 101219
Anti-mouse CD45 PerCP Cy5.5 (Clone
30-F11) Biolegend 103131
Anti-mouse CD25 PE (Clone
PC61) Biolegend 102007
Anti-mouse CTLA4 PE/Dazzle594 (Clone
UC10-4B9) Biolegend 106317
Anti-mouse F4/80 PE-Cy7 (Clone BM8) Biolegend 123113
Anti-mouse CD3 AF647 (Clone
17A2) Biolegend 100209
Anti-mouse MHC II AF700 (Clone
M5/114.15.2) Biolegend 107621
Anti-mouse CD4 APC/Fire750 (Clone
GK1.5) Biolegend 100459
Anti-mouse CD45 BV510 (Clone
30-F11) Biolegend 103137
Anti-mouse PD-1 BV605 (Clone
29F.1A12) Biolegend 135219
Anti-mouse TIM-3 BV711 (Clone
B8.2C12) Biolegend 134021
Anti-mouse LAG-3 BV711 (Clone
C9B7W) Biolegend 125219
Anti-mouse CD4 AF488 (Clone
GK1.5) Biolegend 100425
Anti-mouse CD3 PE (Clone
17A2) Biolegend 100205
Zombie Red Biolegend 423109
Anti-mouse CTLA4 PE Cy7 (Clone
UC10-4B9) Biolegend 106313
Anti-mouse CD8 APC Cy7 (Clone
53-6.7) Biolegend 100713
Anti-mouse IFN-gamma (Clone
XMG1.2) Biolegend 505829
Anti-mouse PD-1 BV650 (Clone

29F.1A12) Biolegend 135243
Anti-mouse CD45 AF488 Cy5.5 (Clone
30-F11) Biolegend 103121
Anti-mouse CD69 PE Cy7 (Clone
H1.2F3) Biolegend 104511
Anti-mouse GZMB AF647 (Clone
GB11) Biolegend 515405
Anti-mouse TNF-a (Clone MP6-XT22) Biolegend 506338

Validation	All antibodies are commercially available and validation information can be found on the manufacture's website.

Eukaryotic cell lines

Policy information about cell lines and Sex and Gender in Research

Cell line source(s)	Human embryonic kidney 293T (Lenti-X cells, purchased from Takara, Inc.), murine glioblastoma Tu2449 (generously provided by Dr. Noriyuki Kasahara, University of California, San Francisco), and human glioblastoma U87 (generously provided by Dr. Noriyuki Kasahara, University of California, San Francisco). Murine glioblastoma SB28 (generously provided by Dr. Hideho Okada, University of California, San Francisco). Human GBM43 and G55 (generously provided by the Mayo Clinic).
Authentication	Human patient derived GBM cell lines were authenticated by short tandem repeat (STR) profiling, all cell lines were routinely tested for mycoplasma contamination, and used at low passage.
Mycoplasma contamination	Human patient derived GBM cell lines were authenticated by short tandem repeat (STR) profiling, all cell lines were routinely tested for mycoplasma contamination, and used at low passage.
Commonly misidentified lines (See ICLAC register)	NA

Animals and other research organisms

Policy information about studies involving animals; ARRIVE guidelines recommended for reporting animal research, and Sex and Gender in Research

Laboratory animals	C57BL/6 and B6C3F1 mice (8–12 weeks old) were obtained from Jackson Laboratories and housed at the University of California, San Francisco. Experiments utilizing Tu2449 and SB28 murine glioblastoma cell lines were conducted under comparable conditions.
Wild animals	NA
Reporting on sex	All in vivo experiments were performed in female mice. Mouse sex was not expected to influence therapeutic efficacy
Field-collected samples	NA
Ethics oversight	Animal experiments were approved by UCSF IACUC (approval #AN105170-02)

Note that full information on the approval of the study protocol must also be provided in the manuscript.

Clinical data

Policy information about clinical studies
All manuscripts should comply with the ICMJE guidelines for publication of clinical research and a completed CONSORT checklist must be included with all submissions.

Clinical trial registration	Human data utilized in this study was from publicly available sources or datasets. No new human data was collected.
Study protocol	Human data utilized in this study was from publicly available sources or datasets. No new human data was collected.
Data collection	Human data utilized in this study was from publicly available sources or datasets. No new human data was collected.
Outcomes	Human data utilized in this study was from publicly available sources or datasets. No new human data was collected.

Plants

Seed stocks	NA
Novel plant genotypes	nA
Authentication	NA

Flow Cytometry

Plots

Confirm that:

☒ The axis labels state the marker and fluorochrome used (e.g. CD4-FITC).

☒ The axis scales are clearly visible. Include numbers along axes only for bottom left plot of group (a 'group' is an analysis of identical markers).

☒ All plots are contour plots with outliers or pseudocolor plots.

☒ A numerical value for number of cells or percentage (with statistics) is provided.

Methodology

Sample preparation	Brain tumors were minced and digested in collagenase type IV (Thermo Fisher Scientific, #17104019) and Deoxyribonuclease I (Worthington Biochemical Corporation) solutions while agitated at 37°C. Tumor suspensions were subsequently filtered through 70 µm filters, and red blood cells were lysed using Ammonium-Chloride-Potassium (ACK) lysing buffer (Lonza).
Instrument	Data acquisition was conducted using an Attune NxT Flow Cytometer (Thermo Fisher Scientific)
Software	Flow cytometry data were analyzed using FlowJo software.
Cell population abundance	Cell population abundances were quantified as percentages of live $CD45^+$ cells unless otherwise indicated. The parent population is noted on the Y axis for each flow cytometry population quantified.
Gating strategy	Flow cytometry data were analyzed using a standard gating strategy. There was sequential gating on singlets, live cells, and $CD45^+$ leukocytes then specific cell populations of interest. Representative gating strategies are shown in the Supplementary Figures.

☒ Tick this box to confirm that a figure exemplifying the gating strategy is provided in the Supplementary Information.
