# Supplementary material for: Non-lytic viral immunotherapy induces long-term glioblastoma survival and tumor-specific immunity without eliciting an antiviral response
Source: Nat Commun. 2026 May 19;17:6613. doi: 10.1038/s41467-026-72746-5 (PMC13381920; doi:10.1038/s41467-026-72746-5)
Supplement: Supplementary file 3 — Transparent Peer Review file [file 41467_2026_72746_MOESM3_ESM.pdf]

nature portfolio

Peer Review File

Non-lytic viral immunotherapy induces long-term glioblastoma survival and tumor-specific immunity without eliciting an antiviral response

Corresponding Author: Professor Manish Aghi

This file contains all reviewer reports in order by version, followed by all author rebuttals in order by version.

Version 0:

Reviewer comments:

Reviewer #1

(Remarks to the Author)
Haddad et al. provides benefits of using replicating non-lytic retrovirus armed with IL-15 receptor-ligand fusion protein complex (RLI) against immunologically cold GBM tumors. The authors successfully show the persistence of the virus until the endpoint with effective enhancement of the activated CD8 T cells and NK cells in the tumor microenvironment (TME). The RRV-RLI significantly improved the survival of two mouse GBM models in comparison to the empty RRV control and PBS control. The authors were also able to observe induction of immunological response within TME using RRV-RLI as shown with Nanostring-based array. The authors also emphasize on the importance of CD8 T cells for the survival benefit observed with RRV-RLI in SB28 tumors and afterwards shows advantages of using TMZ in combination with RRV-RLI to further enhance CD8 TCR repertoire & clonotypes against SB28 tumor using Single Cell RNAseq & TCR sequencing. While the findings of this paper are potentially significant to the field, the following concerns should be addressed prior to publication of the manuscript.
Certain concerns: Issues that must be addressed to ensure the validity of conclusions made by the manuscript. (includes both major & minor concerns)
Concern 1. In Figure 1A, please specify which patient dataset has been used for this study.
Concern 2. In Figure 1D-F, please specify if there is any evidence for the mechanism behind role of IL-15 in reducing M0 Macrophages in GBM context?
Concern 3. In line 98, does the single cell dataset also highlights increase in the expression pattern of immune checkpoints on tumor-infiltrating lymphoid and myeloid populations?
Concern 4. Typo! Change "an" to "a" in line 104.
Concern 5. In Figure 2G-H, did the authors observe a direct impact of RRV-RLI on the expression of immune checkpoint markers on T cells through the co-culture assays? Scale of exhaustion of T cells!!
Concern 6. In Figure 3, authors should also show the secretion of RLI with tumor after 10 days post virus injection.
Concern 7. In chapter "RRV-RLI induces significant anti-tumor modulation of the GBM immune microenvironment", authors should elaborate on the expression of checkpoint markers, state of dendritic cells, macrophages & neutrophiles.
Concern 8. In chapter "RRV-RLI induces significant anti-tumor modulation of the GBM immune microenvironment", the authors also observe increase in the expression of markers relative to neutrophiles. Can the authors explain whether the neutrophiles phenotype is tumor-supportive or pro-inflammatory?
Concern 9. In chapter "RRV-RLI induces significant anti-tumor modulation of the GBM immune microenvironment", as the authors observe a significant increase in exhausted CD8 T cells. Does it go by the hypothesis of enhanced CD8 T cells potency against tumor with RRV-RLI, or the survival benefit of RRV-RLI is happening due to a completely different immune cell subset?
Concern 10. In Line 185, typo for the figure number; 'Fig 3B to "Fig 4B".
Concern 11. In Figure 2F, the comparison has been made between PBS and RRV-RLI to analyze changes in the immune cell populations. The authors should show a similar analysis done between RRV & RRV-RLI to support the effect of RLI in the experiment.
Concern 12. In Figure 2D legend, the author should correctly specify that heatmap belongs to RRV Vs RRV-RLI.
Concern 13. In Figure 5, why was the survival comparison was done on PBS Vs RRV-RLI in-spite of RRV Vs RRV-RLI? A survival comparison between RRV Vs RRV-RLI is important to specifically determine the role of CD4/CD8 T cells via RRV-RLI therapy against SB28 tumor in vivo.

Concern 14. In Figure 5, using CD8 depleting antibody could also reduce CD8+ dendritic cell population which derives the activation of naive CD8 T cells by presenting tumor-specific antigen in the tDLNs. Can the author show that the use of CD8 depleting antibody does not affect the dendritic cell subsets in tDLNs or TME?
Concern 15. In Figure 5G, it seems that CD8 depletion still show benefit of survival in with RRV-RLI, directing towards the importance of potential CD4 T cell mediated RRV-RLI efficacy in SB28 mGBM model. The authors should also show survival data by depleting CD4 cells alone in RRV Vs RRV-RLI treatment groups.
Concern 16. In Figure Supplementary 3E-F, has the anti-PD1 treatment compared between RRV Vs RRV-RLI?
Concern 17. In the chapter "Antitumoral efficacy of RRV-RLI against murine GBM is potentiated by systemic chemotherapy", how does the author justify the use of a replicating non-lytic RRV which could also lead the release of tumor-specific antigens, to that of the use of TMZ in combination of RRV-RLI to enhance the same?
Concern 18. In Figure 6A-B, the authors have shown comparisons of combining TMZ with PBS or RRV-RLI but have not included RRV control with TMZ to justify the benefit of RLI!!
Concern 19. In Supplementary Fig. 4E, we can observe a significant decline in cDC2 conventional DCs with TMZ+RRV-RLI. Does the Single cell RNAseq data reveal more about the effect of this combination on DCs subsets, their functionality & antigen-presentation potential?
Concern 20. In line 319, the authors should figure they are referring the flow cytometry data of!!
Concern 21. In Figure 7E, the dendritic cells population is seemingly reducing with RRV-RLI only treatment in comparison to PBS which is different from the Figure 4F. The authors should reason this difference.

Reviewer #2

(Remarks to the Author)
In the manuscript by Haddad et al., the authors generated a non-lytic replicating retrovirus (RRV) expressing the receptor-linked IL-15 (RLI) and evaluated its anti-tumor efficacy in murine GBM models. The combination treatment efficacy of RRV-RL1 with TMZ is also evaluated. RRV-RL1 treatment prolongs mice survival which is even improved when combined with TMZ. The immune responses, specifically T-cell responses, have been analyzed via the antibody-based depletion strategy and single cell RNA/TCR analysis. Developing the new virus-based therapeutic strategy to treat GBM is clinically relevant. However, the mechanistic action of RRV-RL1 treatment as defined in the current study is largely descriptive. It is not clear if the RRV-RL1 treatment effects result from the secreted receptor-linked–IL-15, or just soluble IL-15. The contribution of NK cells to the therapeutic efficacy of RRV-RL1 is not experimentally evaluated. Some results are not sufficiently support the conclusions and lack clarity (see below). Overall, this manuscript in the current format is not suitable for publication in Nature Communications.

1) Fig. 1G, H: The proportion of cell types in the Alluvial plot (Fig. 1H) does not clearly match those presented in the UMAP plots (Fig. 1G), and what is the cellular identity of APC?
2) Fig. 2, Supplementary Fig.1, Supplementary Fig. 2A and throughout the manuscript: It is not clear if the secreted RLI is a true receptor-linked–IL-15, or just soluble IL-15. Furthermore, it is not clear if the RRV-RL1 treatment effects result from the secreted receptor-linked–IL-15, or just soluble IL-15.
3) Fig. 2F.G: It is not clear about what is the “untreated” group. Are these mice age-matched to the re-challenged mice?
4) Fig. 4: The analysis based on the comparison of RRV-RL1 group to the PBS group cannot clearly distinguish the immune changes as a direct result of anti-RRV or RLI or anti-tumor effect. Fig. 4F: Why exhausted CD8 score is increased in the RRV-RLI group vs PBS? Again, Supplementary Fig. 2E: why exhausted CD8 is higher in RRV-RLI TP vs RRV-RLI EP or RRV TP?
5) Fig. 5: The tumor volume and mouse survival in the RRV-RL1 + isotype control group need to be compared to those in the RRV-RL1 + anti-CD4/CD8 group and those in the RRV-RL1 + anti-CD8 group. Additionally, the tumor volume and mouse survival in the PBS + isotype control group need to be compared to those in the PBS + anti-CD4/CD8 group and those in the PBS + anti-CD8 group.
6) Fig. 4 and Supplementary Fig. 2 indicate that NK cell response is increased in the RRV-RL1 group. Expansion of NK cells is pronounced in RRV-RL1 +/-TMZ group (vs PBS +/-TMZ group) and NK cells are the most abundant in these 2 groups, as shown in Fig. 7C. The contribution of NK cells to tumor progression and mice survival upon RRV-RL1 treatment +/-TMZ needs to be experimentally evaluated.
7) Supplementary Fig. 5B,C: Use of Tox as the only gene indicating CD8 or NKT exhaustion is not sufficient.
8) The results showing that increased osteopontin (Spp1) from myeloid/macrophages may enhance immune activation in RRV-RL1 +/-TMZ groups (Fig. 7G and Supplementary Fig. 5G) are contradictory to the report that myeloid-derived Spp1 drives CD8+ T-cell exhaustion (PMID: 36638785).
9) Increased cross-presentation to CD8+ T-cells by myeloid cells in RRV-RL1 +/-TMZ groups is inferred from the CellChat analysis. A direct experiment proving this is missing.
10) The tumor-reactivity of T-cells and the lack of immunogenicity of the RRV backbone are inferred by TCR analysis. A direct experimental strategy proving the tumor-reactivity without the RRV reactivity of expanded T-cells in RRV-RL1 +/-TMZ groups is required.
11) Supplementary 1F: It is not clear if NK cells or CD8+ T-cells are analyzed here based on the legend.

Reviewer #3

(Remarks to the Author)
The authors have a strong track record in clinical trials of replicating retroviral vectors (RRV) for glioblastoma and have continued to develop these vectors for more effective patient treatment. Despite promising results, Phase III trials revealed that the suicide gene (CD) was insufficient to kill glioma cells and stimulate long-term anti-tumor immunity. In this

manuscript, the authors evaluate a new RRV armed with cDNA encoding a superagonist form of IL-15 (RLI), a cytokine that stimulates NK and CD8+ T cells. Using murine glioma cell lines SB28 and Tu2449, they provide evidence of the efficacy of this new virus, RRV-RLI, including profiling immune cell populations and TCR repertoires using the 10x platform.
There are some concerns in experimental setting; the lack of a control virus in TIL analysis, particularly in Figures 4 and 7, limits the ability to determine the IL-15 specific effects. Additionally, in vivo treatment starting at day 4 post-tumor may coincide with scar healing from needle injection, raising questions about whether RRV-RLI infects parenchymal and innate cells for persistent IL-15 production rather than targeting the tumor itself. This stage mainly recruits NK cells, making RRV-RLI more effective at stimulating NK cells while adaptive immune cells typically start infiltrating at day 6-7 in this model. This timing may eliminate seed tumors before generating a tumor microenvironment. Discussing these points would be appreciated, considering RRV's naturally non-lytic feature.
Major concerns
1) For Figure 1 analysis, the manuscript is missing details on patient samples, data, analysis methods, and the IRB statement.
1. Was tumor size considered when comparing IL-15 high vs. IL-15 low GBM tumors, especially since IL-15 high tumors have fewer tumor cells?
2. Were the resected tumor samples taken from the tumor core or edges? The sampling location can affect the analysis of tumor-infiltrating cells.

• The authors demonstrated that co-culturing T- and NK cells with RRV-RLI promotes their expansion and leads to their activation, as indicated by increased CD69 and Granzyme B expression. Given that RRV is known to infect immune cells and use them as carriers, culturing T cells with the virus in the absence of tumor cells could help determine whether the observed effects are due to direct viral influence on T cells rather than IL-15 secreted by infected tumor cells (i.e, direct viral infection to T cells in co-culture and its impact on T cell function and proliferation)

• Considering that tumor-infiltrating myeloid cells or microglia may infiltrate in response to the virus and potentially limit viral replication, this could reduce IL-15 secretion and contribute to T cell exhaustion near the study endpoint. How long does IL-15 agonist secretion from viruses persist in vivo? It is suggested to analyze native IL-15 gene and RLI transcripts obtained from RNA-seq data (e.g., Fig 4) or through IHC and RT-PCR at analysis points.

• In Figure 4D, the heatmap shows increased expression of immune checkpoint inhibitors like CTLA-4, PD-1, and TIGIT in the treatment group, and Figure 4F shows an increased immune score for exhausted CD8 T cells. Results and discussion lack this data interpretation. Could the T cells be exhausted due to an immunosuppressive environment promoted by the virus or tumor factors? Did the authors assess if immunosuppressive cells like TAMs or MDSCs increased with virus infection? Were other cytokines like IFN-γ or IL-10 assessed in the tumor microenvironment or in vitro, as they might contribute to T cell exhaustion? Probably, it would be appreciated to add more discussion.

RRV-RLI monotherapy significantly improved survival in the Tu2449 GBM model, with 90% of mice showing long-term survival and developing immunologic memory. However, this effect was not observed in the SB28 model. What factors or aspects of the tumor microenvironment contribute to the different outcomes in SB28 and Tu2449? Could this difference be due to variations in infectivity or replication of the virus in these models?

The TCR analysis concludes that the identified TCRs represent a tumor-specific response enhanced by RRV-RLI and RRV-RLI+TMZ rather than an antiviral response to RRV-RLI. However, this statement needs supportive evidence and might be better placed in the discussion section. Using scRNA with TCR profiling to identify matching alpha-beta chains at the single-cell level would support this claim by addressing specific antigen-responsive T cells with reconstituted TCR analysis.
Minor concerns
In general, where scRNA-seq data is presented, the font size of text and axis labels appears too small, making it difficult to read. Below are specific instances where this issue needs attention:
• Figure 1A-C, 2B, 6D-G, 6H, 7: The font size of the axis labels, annotation and text is too small, making it difficult to read.
• Figure 4: The axis labels and text throughout the figure—except for Figure 4F—are not legible and should be adjusted.
Figure 2B: The axes of the flow cytometry data are not labeled. It would be beneficial to include axis titles instead of relying solely on color coding.
Line 185: 'Figure 3B' should be corrected to 'Figure 4B.'
These revisions will enhance readability and clarity across the figures.

Reviewer #4

(Remarks to the Author)
I co-reviewed this manuscript with one of the reviewers who provided the listed reports. This is part of the Nature Communications initiative to facilitate training in peer review and to provide appropriate recognition for Early Career Researchers who co-review manuscripts.

Version 1:

Reviewer comments:

Reviewer #1

(Remarks to the Author)
The authors have answered all my questions.

Reviewer #2

(Remarks to the Author)
The revised manuscript has largely addressed the prior concerns. However, a few minor issues still need attention:

1. The line numbers cited in the response/rebuttal do not match those in the revised manuscript.

2. Some figures appear to be cited incorrectly in the response/rebuttal (e.g., The cross-presentation assay was shown in supplementary Fig. 6A,B, not in Supplementary Fig. 5H,I).

3. The references in the response/rebuttal appears out of order. For example, ref 10 (PMID: 25972484) is cited for "this label represents antigen-presenting cells as defined by singleR unsupervised cell labeling and CIITA, CD74, and CD86 expression"; ref 11 (PMID: 19889936) is cited for "…validated in the literature as producing receptor-linked-IL-15 that does not degrade into soluble IL-15"; ref 4 (PMID: 11702064) is cited for "prior work" related to "RRV infection alone does not significantly impact tumor growth…"
In addition, while 25 references are cited, only 21 are listed. Please ensure all cited references are included and correctly ordered.

Overall, the reviewer would appreciate a thorough cross-check of both the revised manuscript and the response/rebuttal to ensure internal consistency throughout.

Fig. 1 G,H legend: the prior comment was not requesting a definition of APC; rather, it asked what cell type represents APC (e.g., macrophage or DC). Please clarify this explicitly in the legend/text.

Mediating comments to R#3 responses:

I have reviewed the response to Reviewer #3 and believe the authors have addressed the requests adequately. The remaining issues are minor and primarily relate to inconsistencies between the line numbers cited in the response and those in the revised manuscript, which made cross-referencing difficult. Although all points could be verified through a full review of the manuscript, this process was time-consuming.

Reviewer #4

(Remarks to the Author)
I co-reviewed this manuscript with one of the reviewers who provided the listed reports. This is part of the Nature Communications initiative to facilitate training in peer review and to provide appropriate recognition for Early Career Researchers who co-review manuscripts.

Open Access This Peer Review File is licensed under a Creative Commons Attribution 4.0 International License, which permits use, sharing, adaptation, distribution and reproduction in any medium or format, as long as you give appropriate credit to the original author(s) and the source, provide a link to the Creative Commons license, and indicate if changes were made.
In cases where reviewers are anonymous, credit should be given to 'Anonymous Referee' and the source.
The images or other third party material in this Peer Review File are included in the article's Creative Commons license, unless indicated otherwise in a credit line to the material. If material is not included in the article's Creative Commons license and your intended use is not permitted by statutory regulation or exceeds the permitted use, you will need to obtain permission directly from the copyright holder.
To view a copy of this license, visit https://creativecommons.org/licenses/by/4.0/

March 18, 2026 [updated to correct line numbers and figure numbering based on new Reviewer 2 comments]

Dear *Nature Communications* Editor and Reviewers,

We are pleased to submit the revised version of our manuscript, "**Non-lytic viral immunotherapy induces long-term glioblastoma survival and tumor-specific immunity without eliciting an antiviral response"** for consideration for publication in *Nature Communications*.

We appreciate the reviewers' insightful comments on the manuscript. In response, we performed multiple novel experiments that expand the mechanistic findings, scope, and translational impact of our work. During the revision process, we completed eight new *in vivo* studies and three new *in vitro* studies, resulting in numerous new main and supplementary figure panels, the highlights of which include:

- To better understand the mechanisms around RRV-RLI treatment efficacy and synergy with temozolomide, we performed new T cell co-culture assays and dendritic cell cross-presentation assays.
- We completed CD4 T-cell and NK cell depletion experiments and expanded our previous CD8 depletion to a larger sample size to gain more clarity into the role of these cells in RRV-RLI efficacy.
- A common reviewer concern involved distinguishing the effects of the RRV backbone from the RLI transgene, and our choice to use intratumoral PBS treatment rather than RRV empty vector as a control arm in depletion experiments. After a review of the data with the editor, the editor told us that, based on the lack of survival benefit with RRV alone demonstrated in the original manuscript, she supported our choice not to include RRV empty vector as a control in depletion experiments but asked that we comprehensively compare intratumoral immune cells after intratumoral RRV-RLI, RRV, and PBS treatments via a large *in vivo* study. These data have been added to the revised manuscript and demonstrate no immune response to RRV alone, which, in combination with the lack of survival benefit from RRV treatment, implicates the RLI payload in the observed anti-tumor immune response occurring with RRV-RLI treatment.
- To strengthen the translational impact of our work, we added *in vivo* studies demonstrating the efficacy of RRV-RLI at a later time point and in a reduced dose.
- We validated RRV-RLI gene delivery and RLI protein expression ten days after administration, investigated viral biodistribution in the contralateral brain, and showed viral spread in multiple human patient-derived cell lines.

Below, we respond to the reviewer comments in a point-by-point fashion:

<u>Reviewer #1's Comments:</u>

Haddad et al. provides benefits of using replicating non-lytic retrovirus armed with IL-15 receptor-ligand fusion protein complex (RLI) against immunologically cold GBM tumors. The authors successfully show the persistence of the virus until the endpoint with effective enhancement of the activated CD8 T cells and NK cells in the tumor microenvironment (TME). The RRV-RLI significantly improved the survival of two mouse GBM models in comparison to the empty RRV control and PBS control. The authors were also able to observe induction of immunological response within TME using RRV-RLI as shown with Nanostring-based array. The authors also emphasize on the importance of CD8 T cells for the survival benefit observed with RRV-RLI in SB28 tumors and afterwards shows advantages of using TMZ in combination with RRV-RLI to

further enhance CD8 TCR repertoire & clonotypes against SB28 tumor using Single Cell RNAseq & TCR sequencing. While the findings of this paper are potentially significant to the field, the following concerns should be addressed prior to publication of the manuscript. Certain concerns: Issues that must be addressed to ensure the validity of conclusions made by the manuscript. (includes both major & minor concerns)

Comment #1: In Figure 1A, please specify which patient dataset has been used for this study.

Response #1: We thank the reviewer for their comment. For **Figures 1A-C**, we used publicly available data from the TCGA Pan Cancer Atlas. In the revised manuscript, we have mentioned that this data derived from the TCGA Pan Cancer Atlas in the revised results section (Page 4, Lines 88-89), provided additional details on the bulk RNA sequencing data in the Methods section in the subsection titled "Human bulk RNA-sequencing analysis," and added a figure reference to the end of this section to more clearly tie these methods to **Figures 1A-C** (Page 17, Line 781).

Comment #2: In Figure 1D-F, please specify if there is any evidence for the mechanism behind role of IL-15 in reducing M0 Macrophages in GBM context?

Response #2: We thank the reviewer for their comment. Figures 1D-F present immune-cell frequencies deconvolved from bulk RNA-seq of IL-15 high versus IL-15 low GBM samples, demonstrating a rise in $CD8^+$ T cells and monocytes and a relative reduction in non-polarized M0 macrophages. Two non-exclusive mechanisms likely contribute to the reduction in M0 macrophages: (1) Direct engagement of IL-15 signaling pathways on tumor associated macrophages which has been shown to induce an inflammatory (M1-like) state and thus reduce the M0 compartment;[1] and (2) Preclinical studies, including ours, in GBM have also shown an overall relative (not absolute) decrease in the entire myeloid compartment in response to IL-15 in the GBM TME,[2] which we hypothesize to be due to increased IL-15 mediated tumor infiltration of $CD8^+$ T-cells altering cellular proportions within the tumor and lowering the *relative* abundance of cell types other than $CD8^+$ T-cells, such as M0 Macrophages. We have added a sentence to our discussion highlighting these points (Page 11, Lines 437-440).

Comment #3: In line 98, does the single cell dataset also highlight increase in the expression pattern of immune checkpoints on tumor-infiltrating lymphoid and myeloid populations?

Response #3: We assessed the public GBM single-cell dataset we used to compare IL-15 high and low tumors for expression of classic checkpoint molecules (PDCD1/PD-1, CTLA4, LAG3, TIGIT and HAVCR2/TIM-3) in the tumor-infiltrating T cell and myeloid compartments. All molecules except HAVCR2 were detected in ≤5 % of lymphoid cells and were virtually absent from myeloid clusters, precluding a robust differential-expression analysis. There was no difference in the expression of HAVCR2, the only checkpoint molecules expressed by tumor-infiltrating T-cells and myeloid cells at sufficient levels to analyze, between the groups. This is a recognized limitation of single-cell RNA-seq, particularly for immune transcripts in brain tumors with minimal lymphocyte content. As a result, we cannot draw significant conclusions around differential checkpoint expression in these IL-15 high and low GBM tumors. To clarify this limitation, we have added a sentence to the results discussion (Page 4, Line 101-104). However, to further investigate the relationship between IL-15 signaling and immune checkpoint expression, we have performed additional co-culture experiments, which we discuss in **Response #5** below.

Comment #4: Typo! Change "an" to "a" in line 104.

Response #4: Thank you, we have corrected the an to a (Page 4, Line 107).

Comment #5: In Figure 2G-H, did the authors observe a direct impact of RRV-RLI on the expression of immune checkpoint markers on T cells through the co-culture assays? Scale of exhaustion of T cells!!

Response #5: We thank the reviewer for this comment. We have completed additional experiments where we repeated our T cell co-culture study and simultaneously assessed both T cell exhaustion/checkpoint marker expression and activation in response to RLI signaling. In this experiment, we found a small increase in PD-1 expression, likely consistent with activation rather than exhaustion, given the concordant increase in CD69 expression and lack of other concurrently expressed exhaustion markers (no change in expression of immune checkpoint markers TIM3, LAG3, and CTLA4, and no increase in double positive PD1/TIM3 and PD1/LAG3 populations was noted in response to RLI signaling), since PD-1 expression in the absence of expression of other exhaustion markers in the setting of CD69 co-expression is a sign of T-cell activation rather than exhaustion.[3] These findings suggest that there is not a shift towards checkpoint-high or exhausted T cell states in the presence of RLI. We have added this new data to the results section (**Supplementary Fig. 1I**, Page 5, Lines 156-161).

Comment #6: In Figure 3, authors should also show the secretion of RLI with tumor after 10 days post virus injection.

Response #6: Thank you for this comment. We agree and have performed additional *in vivo* experiments to address this concern. We now provide quantitative evidence of retroviral genome DNA (qPCR) and RLI protein (ELISA) intratumorally at 10 days post virus injection. These findings support the efficient delivery of RLI via viral tumor transduction. We have added these data to the results section of the manuscript (**Supplementary Fig. 2A-B**, Page 6, Lines 180-183).

Comment #7: In chapter "RRV-RLI induces significant anti-tumor modulation of the GBM immune microenvironment", authors should elaborate on the expression of checkpoint markers, state of dendritic cells, macrophages & neutrophiles.

Response #7: We thank the reviewer for their suggestion. While **Figure 4** is representative of bulk transcriptomic data generated via NanoString multiplex technology and limits the identification of cell specific states, we utilized our single cell RNA seq data set (presented in **Figure 7**) to address these points and examine the cell types that the reviewer mentioned. In our scRNA sequencing data, we find several changes in the immune cell types that the reviewer has asked about in response to RLI-RRV therapy:

- With regards to dendritic cells (including mCD1 and mCD2 populations), we see a reduction in markers of immature dendritic cells (Ifitm1 Log2FC -2.14, p_val_adj =3.29E-23, Klrb1b Log2FC -2.21, p_val_adj=4.28E-16, Adgrg5 Log2FC -2.59, p_val_adj=1.54E-13, Il1b Log2FC -1.35, p_val_adj=1.99E-19) and an increase in markers of dendritic cell activation (Il4i1 Log2FC 1.99, p_val_adj = 4.12E-12), antigen processing/presentation (Psme2 Log2FC 0.83, p_val_adj=1.25E-9, B2m Log2FC 0.53, p_val_adj=7.06E-9, Anxa2 Log2FC 0.97, p_val_adj=1.71E-8), and migration (Itgb3 Log2FC 3.14, p_val_adj =7.53E-5). These changes suggest an overall shift in dendritic cell populations from immature to mature and activated following RRV-RLI treatment *in vivo*. We have amended the results to reflect these results (**Supplementary Figure 5H** Page 9, Lines 358-361).
- Macrophage populations also experience a shift from a tolerogenic state to a reactive, phagocytic, and activated state in response to RRV-RLI treatment. In the presence of RRV-RLI, we observe a reduction in genes associated with immaturity (Il1r2 (LOG2FC -4.11, p_val_adj=6.37E-77, Pilra (LOG2FC -3.43, p_val_adj=5.27E-99) and tolerogenicity

(Clec4n (LOG2FC -3.38, p_val_adj=3.29E-133). Concurrently, we observe an increase in genes related to reactivity (Lgals3 LOG2FC 2.09, p_val_adj=3.81E-141), lysosomal activity (Ctsb LOG2FC 1.15, p_val_adj=1.32E-93), and complement/cytokine signaling (C3 LOG2FC 1.70, p_val_adj=1.89E-40, Ccl2 Log2FC 2.16, p_val_adj=9.78E-52, Ccl7 Log2FC 2.95, p_val_adj=1.11E-72) We have amended the results to reflect these results (**Supplementary Figure 5G,** Page 9, Lines 358-361).

- As mentioned above, we don't see checkpoint markers as significantly differentially expressed genes between treatment groups in myeloid populations. Comparisons in checkpoint marker expression between the different treatment groups in lymphocytes is limited given the very low number of infiltrating T cells in PBS treatment mice. When assessing our scRNA sequencing data, we observe that less than 1% of infiltrating lymphocytes are labeled as "exhausted $CD8^+$ T cells" in all treatment groups (**Figure 7E**). Furthermore, as discussed in **Response 5** we do not see a significant change in checkpoint marker expression in T cells co-cultured with RRV-RLI infected tumor cells in vitro (**Supplementary Figure 1I**).
- We discuss neutrophil populations specifically in **Response #8** below.

Comment #8: In chapter "RRV-RLI induces significant anti-tumor modulation of the GBM immune microenvironment", the authors also observe increase in the expression of markers relative to neutrophils. Can the authors explain whether the neutrophil phenotype is tumor-supportive or pro-inflammatory?

Response #8: We thank the reviewer for their insightful comment. Figure 4 is representative of bulk transcriptomic data (Nanostring) that makes it difficult to infer the state of specific cell populations. As such, we have turned to our single cell RNA sequencing data set (**Figure 7**) to further assess infiltrating neutrophil populations and gain additional insight into the infiltrating neutrophil phenotypes. Our scRNA data reveals that infiltrating neutrophils are a relatively rare population in the tumor infiltrating immune milieu (e.g., only 23 neutrophils in the PBS + Vehicle group), limiting the conclusions from our insights. However, when we compare infiltrating neutrophils between RRV-RLI + TMZ and PBS + TMZ, we observe an overall decrease in markers of a tumor-supportive myeloid-derived suppressor cell (MDSC) phenotype in mice treated with RRV-RLI. This is supported by reduced expression of Arg1 (LOG2LOG2FC -3.57, p_adj = 4.29E-07), Chil3 (LOG2FC -3.84, p_adj = 5.58E-24), Mmp8 (LOG2FC -2.8, p_adj = 2.44E-10), Lyz2 (LOG2FC -2.01, p_adj = 6.05E-9), Apoe (LOG2FC -1.87, p_adj=6.81E-6) in neutrophils infiltrating RRV-RLI treated mice relative to control. This is consistent with reduced tumor supportive/immune suppressive programs in RRV-RLI treatment. We have added these findings to the results section of our manuscript (Page 9, Lines 358-361) and to **Supplementary Figure 5F.**

Comment #9: In chapter "RRV-RLI induces significant anti-tumor modulation of the GBM immune microenvironment", as the authors observe a significant increase in exhausted CD8 T cells. Does it go by the hypothesis of enhanced CD8 T cells potency against tumor with RRV-RLI, or is the survival benefit of RRV-RLI is happening due to a completely different immune cell subset?

Response #9: We thank the reviewer for this comment. While **Figure 4F** demonstrates an increase in the immune infiltration score associated with exhausted $CD8^+$ T cells in the RRV-RLI treatment group when compared to control, we believe that this is reflective of the absolute increase in the number of $CD8^+$ T cells rather than an increase in the relative ratio of exhausted and non-exhausted $CD8^+$ T cell populations. Indeed, when we assess the ratio of exhausted to non-exhausted $CD8^+$ T cells, the ratio in the RRV-RLI treatment group is more favorable than that observed in the control group (1.2 vs. 0.84, p=0.04, Student's t-test). To make this clearer, we have added a sentence to the results section highlighting the favorable exhausted to non-

exhausted ratio in RRV-RLI treatment (Page 6, Lines 217-220). In addition, we have performed additional T cell co-culture experiments demonstrating no significant increase in exhausted T cell populations after exposure to RRV-RLI infected tumor cells, as discussed in **Response 5.** Our scRNA dataset also supports that there is not proportionally higher T cell exhaustion in RRV-RLI treated mice, as discussed in **Response 7.**

Regarding the reviewer's question of whether the survival benefit of RRV-RLI could be happening due to a completely different immune cell subset, we do believe that T cells are the primary effector cell type of RRV-RLI treatment response based on the depletion studies in **Figure 5**, where dual CD4/CD8 depletion abrogates the treatment benefit of RRV-RLI treatment, but there is no significant effect on RRV-RLI benefit with NK cell depletion.

Comment #10: In Line 185, typo for the figure number; 'Fig 3B to "Fig 4B".

Response #10: Thank you, we have corrected the Figure number to 4B (Page 6, Line 206).

Comment #11: In Figure 2F, the comparison has been made between PBS and RRV-RLI to analyze changes in the immune cell populations. The authors should show a similar analysis done between RRV & RRV-RLI to support the effect of RLI in the experiment.

Response #11: We thank the reviewer for this comment. We have completed the requested experiment to assess the impact of RRV on the immune microenvironment by performing additional *in vivo* experiments where we directly compare immune infiltration in RRV vs. RRV-RLI-treated tumors at a matched timepoint via flow cytometry. This flow cytometric analysis demonstrates significantly higher infiltration of $CD3^+$ T cells, $CD8^+$ T cells, NKT cells, and NK cells in RRV-RLI treatment groups relative to both PBS and RRV treatment groups, with no significant difference between PBS and RRV treatments (**Figure 6I-M**). Similarly, RRV-RLI results in a proportional decrease in infiltrating $CD11b^+$ cells relative to RRV and PBS treatments with no difference between RRV and PBS groups (**Figure 6N**). We have amended the results section (Page 8, Line 298-311) and **Figure 6** to reflect these findings.

In summary, transcriptomic (**Supplementary Figure 2E-H**), co-culture experiments (**Figure 2G-I, Supplementary Figure 1D-H**), flow cytometric (**Figure 6I-N**), and survival data (**Figure 3B-E**) demonstrate no significant difference between PBS and RRV treatment groups, whereas RRV-RLI demonstrated striking differences from both. These data support that the immune modulation and therapeutic benefit of RRV-RLI is due to the RLI payload rather than the RRV vector backbone. We have added additional reasoning around this topic to the discussion section (Page 12, Line 476-480).

Comment #12: In Figure 2D legend, the author should correctly specify that heatmap belongs to RRV Vs RRV-RLI.

Response #12: Thank you very much for your comment. We have amended **Supplementary Figure 2** legend to reflect that **Panel D** (now **Panel G** after revisions) compares RRV-RLI to RRV.

Comment #13: In Figure 5, why was the survival comparison was done on PBS Vs RRV-RLI in-spite of RRV Vs RRV-RLI? A survival comparison between RRV Vs RRV-RLI is important to specifically determine the role of CD4/CD8 T cells via RRV-RLI therapy against SB28 tumor in vivo.

Response #13: We thank the reviewer for their insightful comment. We discussed the use of PBS as a control group with the editor, who informed us that we did not need to use RRV-treated tumors as a survival control group in the depletion study, but that we should instead perform further immune profiling of RRV-treated tumors to demonstrate similarity to PBS-treated tumors (as we did in new **Figures 6I-N**). We concur with the editor's rationale for this decision based on our co-culture experiments (**Figure 2G-I, Supplementary Figure 1D-H**), *in vivo* transcriptomic (**Supplementary Figure 2E-H**), *in vivo* flow cytometric (**Figure 6I-N**), and survival data (**Figure 3B-E**) revealing no significant difference between PBS and RRV treatment groups, whereas RRV-RLI demonstrated differences from both in each of these critical parameters. Indeed, in addition to our own data, prior work [4] has demonstrated that RRV infection alone does not significantly impact tumor growth, survival, or immune infiltration compared to PBS controls. Given the lack of survival difference between RRV and PBS alone, we wouldn't expect to see a difference in CD4/CD8 depletion survival outcome between RRV and PBS-treated mice. Our mechanistic goal was to assess if the survival benefit of the clinically intended therapy (RRV-RLI) depends on various effector cell populations (CD4/CD8/NK) and we believe that using PBS as the baseline isolates the contribution of these populations in the most translationally relevant fashion. For additional clarity, we have clarified this rationale in the discussion section of the manuscript (Page 12, Line 476-480).

Comment #14: In Figure 5, using CD8 depleting antibody could also reduce CD8+ dendritic cell population which derives the activation of naive CD8 T cells by presenting tumor-specific antigen in the tDLNs. Can the author show that the use of CD8 depleting antibody does not affect the dendritic cell subsets in tDLNs or TME?

Response #14: We thank the reviewer for this helpful comment. In our study, both flow cytometry and single-cell RNA sequencing revealed that CD8$^+$ dendritic cells were so sparse in our murine GBM tumor microenvironment (<0.75% of infiltrating immune cells, **Supplementary Figure 4E**) that the impact of CD8 depletion on these cells could not be reliably assessed. We have added a panel to **Supplementary Figure 4E** demonstrating the lack of this population on flow cytometry and have clarified this in the results section (Page 8, Lines 307-311). This finding is consistent with prior studies assessing the role of CD8$^+$ T cells in immunotherapy models using CD8-depleting antibodies, which have not required separate evaluation of CD8+ dendritic cell populations to ensure that their findings are due to effects of CD8 depletion on T-cells.[5,6] Furthermore, we found that CD8 depletion alone did not impact RRV-RLI's therapeutic benefit, with only the combination of CD8 and CD4 depletion impacting RRV-RLI's therapeutic benefit, suggesting that RRV-RLI requires the two T-cell subsets together for its effect rather than any CD8$^+$ cells alone. In summary, we believe our data makes it unlikely that off-target depletion of CD8 dendritic cells accounts for the abrogation of RRV-RLI therapeutic efficacy observed in the CD4/CD8 depletion experiment. We have amended the discussion to highlight the need for CD4 and CD8 T cells for RRV-RLI efficacy (Page 12, Lines 487-496). and the methods to reflect the decision not to evaluate dendritic cell populations (Page 16, Lines 680-682).

Comment #15: In Figure 5G, it seems that CD8 depletion still show benefit of survival in with RRV-RLI, directing towards the importance of potential CD4 T cell mediated RRV-RLI efficacy in SB28 mGBM model. The authors should also show survival data by depleting CD4 cells alone in RRV Vs RRV-RLI treatment groups.

Response #15: We thank the reviewer for their comment. In accordance with the reviewer's suggestion, we did perform a CD4$^+$ T cell depletion experiment that continues to show a significant survival benefit in mice treated with RRV-RLI relative to PBS control (18 vs. 44 days, $p=0.0001$, Log-Rank, **Figure 5H**). We have amended the results (Page 7, Lines 250-252) and **Figure 5** to

reflect these findings. We used PBS control as the control arm given the lack of survival benefit or immunologic change seen with RRV alone treatment, as discussed in **Response 13**. Furthermore, to increase the reliability with which our CD8 depletion results (which revealed a lack of therapeutic benefit for RRV-RLI with CD8 depletion that was barely statistically meaningful) could be interpreted alongside our other depletion results, we repeated the CD8 depletion experiment concurrently with our other depletion studies with a larger number of mice. Upon repeat of the CD8 depletion, we do now observe a significant survival difference between RRV-RLI and PBS groups in the setting of CD8 depletion (43.0 days vs. 18.0 days, p=0.03, Log-Rank Mantel-Cox, **Figure 5F**), suggesting a compensatory response from CD4 T cells and NK T cells in this setting. These findings suggest that $CD8^+$ and $CD4^+$ T cells coordinate to serve as the primary co-drivers of RRV-RLI therapeutic efficacy, with both together needed for this survival benefit in a manner that the therapeutic benefit can continue as long as one of these T-cell subtypes is present, but is lost once both are gone, a versatility that has implications for the translatability of this approach where patients could derive a benefit unless they had a dramatic reduction in levels of both T-cell subtypes. We have amended the results (Page 7, Lines 246-252) and **Figure 5** to reflect these findings.

Comment #16: In Figure Supplementary 3E-F, has the anti-PD1 treatment compared between RRV Vs RRV-RLI?

Response #16: We thank the reviewer for this comment. In **Supplementary Figure 3E–F** (now **Supplementary Figure 3F-G**), anti-PD1 treatment was compared to isotype control in the context of PBS-treated tumors, not RRV-treated tumors. This was done because the goal was to establish whether anti-PD1 alone could improve survival or tumor control in the highly immunosuppressive SB28 model. Our findings were consistent with previous reports demonstrating that SB28 is not responsive to checkpoint blockade.[7] In **Supplementary Figure 3H-I**, we then tested anti-PD1 in combination with RRV-RLI, showing that even with enhanced immune infiltration, PD-1 blockade did not significantly improve outcomes relative to RRV-RLI alone. We did not include an RRV + anti-PD1 arm, as RRV monotherapy has minimal survival or immunologic effects in this model (see **Responses 11 and 13**), a decision supported by the editor in our discussion with her. The PD-1 experiment also clarified that PD-1 signaling alone was not a primary driver of tumor progression after successful initial treatment with RRV-RLI.

Comment #17: In the chapter "Antitumoral efficacy of RRV-RLI against murine GBM is potentiated by systemic chemotherapy", how does the author justify the use of a replicating non-lytic RRV which could also lead the release of tumor-specific antigens, to that of the use of TMZ in combination of RRV-RLI to enhance the same?

Response #17: We thank the reviewer for this comment. Replicating retroviral vectors do not lyse cells as part of their lifecycle, making them unique relative to most vectors used in cancer treatment which have been traditional oncolytic vectors such as Herpes or Adenovirus. As such, replicating retroviral infection alone does not lead to significant tumor cell death and the therapeutic benefit is primarily generated from the payload that is delivered. This is reflected in the lack of survival benefit or immunologic change seen in RRV alone treatment when compared to RRV-RLI. We posit that combination of RRV-RLI with TMZ is synergistic because TMZ leads to some amount of tumor cell death, which provides additional antigens for the immune system to generate an anti-tumor immune response. We believe this is supported by our single cell RNA and T-cell receptor sequencing data which shows that mice who receive RRV-RLI in combination with TMZ have increased T cell receptor clones relative to RRV-RLI mice alone. Our data also demonstrates that RLI expression in the TME increases antigen presentation pathways, further supporting this proposed mechanism.

To further clarify the role of TMZ releasing tumor antigens in its synergy with RRV-RLI, in the revised manuscript, we have performed a functional cross-presentation assay. In this experiment, we utilize SB28 tumor cells expressing ovalbumin (OVA) and assess the ability of dendritic cells pulsed with their supernatant to activate OT-1 CD8 T cells (which recognize OVA SIINFEKL peptide presented by the MHC class I). We compare OT-1 activation between SB28 OVA cells treated with TMZ to those without TMZ treatment. In SB28 OVA cells treated with TMZ, we see an increase in downstream OT-1 T cell activation (as evidenced by $CD69^+$, 13.8% vs. 8.8%, $p<0.05$, Welch's t-test, **Supplementary Figure 6A**) and cell proliferation (as evidenced by $Ki67^+$, 11.7% vs. 7.2%, $p<0.05$, Welch's t-test, **Supplementary Figure 6B**). These data support a model where TMZ cell death increases antigen supply, which then synergizes with RRV-RLI enhancement of T cell, NK cell, and antigen presentation responses. We have amended the results section (Page 10, Lines 375-380), **Supplementary Figure 6A-B,** and the discussion (Page 13, Lines 527-531) to reflect these findings.

Comment #18: In Figure 6A-B, the authors have shown comparisons of combining TMZ with PBS or RRV-RLI but have not included RRV control with TMZ to justify the benefit of RLI!!

Response #18: We thank the reviewer for this comment. We discussed this with the editor who felt that we did not need to include a RRV+TMZ treatment arm because prior data in our model demonstrated that RRV alone (without a therapeutic payload) has minimal effects on tumor growth, survival, or immune modulation (as discussed in **Responses 11 and 13**). Specifically, RRV alone showed no significant benefit or difference compared to PBS in survival or immune infiltration. Given this lack of effect, and to minimize additional animal use, we focused on comparing PBS ± TMZ and RRV-RLI ± TMZ to directly evaluate the efficacy of TMZ treatment in combination with RRV-RLI. To further address the reviewer's astute comment, and at the editor's suggestion, we completed an additional *in vivo* study and flow cytometry of SB28 tumors from mice treated with RRV±TMZ. We added this data to **Figure 6I-N**. We demonstrate no difference in immune cell infiltration between mice treated with RRV ± TMZ and PBS ± TMZ. In addition, we show that there is a significant increase in $CD3^+$ T cells, $CD8^+$ T cells, NKT cells, and NK cells in mice treated with RRV-RLI ± TMZ when compared to RRV ± TMZ. We have added this new data to the results section (Page 8, Line 291-303) and further explained the rationale behind using PBS as the primary control group for these experiments and others in the discussion section (Page 12, Line 476-480).

Comment #19: In Supplementary Fig. 4E, we can observe a significant decline in cDC2 conventional DCs with TMZ+RRV-RLI. Does the Single cell RNAseq data reveal more about the effect of this combination on DCs subsets, their functionality & antigen-presentation potential?

Response #19: We thank the reviewer for this thoughtful comment. As part of these revisions, we repeated flow cytometric analysis of *in vivo* mouse SB28 tumors treated with RLI-RRV±TMZ, RRV±TMZ, and PBS±TMZ to avoid batch effects. When analyzing these new samples, we no longer see the small but significant difference noted in cDC populations between RRV-RLI + Vehicle and RRV-RLI + TMZ that you refer to. In fact, we do not see a significant difference in infiltration of $CD11b^+CD11c^+MHCII^+$ immune cells between any treatment group (**Supplementary Figure 4D**). We do see a small but significant increase in RRV-RLI treatment groups (no difference between Vehicle and Control) in infiltrating $CD11b^+CD11c^+MHCII^+$ $CD8^+$ immune cells (**Supplementary Figure 4E**), but these populations are so rare in the tumors (<0.75%) that it makes gleaning true biological insights difficult. To further investigate the effect of RRV-RLI + TMZ on dendritic cells, we have utilized our single-cell RNA sequencing data. When evaluating overall myeloid populations, we see an increase in antigen processing/presentation machinery that is

highest in RRV-RLI+TMZ followed by RRV-RLI+Vehicle, suggesting synergy between the increased antigen presence afforded by TMZ treatment and the RLI payload (**Figure 7F**). We see a similar trend in dendritic cell populations, specifically between RRV-RLI + TMZ and RRV-RLI + Vehicle. In the setting of RRV-RLI+TMZ treatment, tumor-infiltrating DCs express increased markers of antigen presentation machinery (Ly75 Log2FC 1.73, p_val_adj=1.06E-16, H2-Q4 Log2FC 0.97, p_val_adj=7.4E-05) as well as markers of activation (IL12b Log2FC 1.82, p_val_adj=9.05E-10). We have added these findings into the results section (Page 9, Line 361-363).

Comment #20: In line 319, the authors should figure they are referring the flow cytometry data of!!

Response #20: We thank the reviewer for their comment. We have amended the sentence to reflect that we are referencing the flow cytometry data in **Figure 6N** which demonstrates a reduction in tumor-infiltrating CD11b myeloid cells (Page 8, Line 304-307).

Comment #21: In Figure 7E, the dendritic cell population is seemingly reducing with RRV-RLI only treatment in comparison to PBS which is different from the Figure 4F. The authors should reason this difference.

Response #21: We thank the reviewer for this insightful comment. We assess tumor-infiltrating dendritic cells using three different modalities in the manuscript- bulk transcriptomic analysis (**Figure 4F**), flow cytometric analysis (**Supplemental Figure 4D-E**), and 5' single cell RNA sequencing (**Figure 7E**). The reviewer points out that in **Figure 4F** the dendritic cell transcriptomic tumor infiltrating score appears increased in the RRV-RLI treatment group, while it appears qualitatively slightly decreased in the single cell RNA sequencing in **Figure 7E**. In our flow cytometric analysis (**Supplemental Figure 4D**), we do not see a significant difference in overall $CD11b^{+}CD11c^{+}MHCII^{+}$ immune cell infiltration between RRV-RLI and PBS treatment groups. In addition, in both our single-cell RNA sequencing data and bulk transcriptomic data we see an increase in antigen presentation pathways and expression of MHC-related genes. As such, our conclusion is that intratumoral RLI expression does not lead to grossly increased dendritic cell infiltration but rather increases antigen presentation pathways on existing antigen-presenting cells in the tumor microenvironment. This aligns with the IL-15 literature, which demonstrates that IL-15 supports antigen presentation and increases MHC, CD40, and CD86 expression on dendritic cells.[8,9] In terms of integrating our results across the three platforms as the reviewer is asking for in this comment: as increased antigen presentation pathways identified by single-cell RNA sequencing (**Figure 7F** and **Supplementary Figure 5H)** may slightly inflate calculated immune infiltration scores, which is based on transcriptomic gene expression (**Figure 4F**), without leading to an increase in gross infiltration on flow cytometric analysis (**Supplementary Figure 4D**). We have added an additional point to our discussion, clarifying this difference and appreciate the reviewer's point (Page 11, Lines 470-472).

<u>Reviewer #2's Comments:</u>

In the manuscript by Haddad et al., the authors generated a non-lytic replicating retrovirus (RRV) expressing the receptor-linked IL-15 (RLI) and evaluated its anti-tumor efficacy in murine GBM models. The combination treatment efficacy of RRV-RL1 with TMZ is also evaluated. RRV-RL1 treatment prolongs mice survival which is even improved when combined with TMZ. The immune responses, specifically T-cell responses, have been analyzed via the antibody-based depletion strategy and single cell RNA/TCR analysis. Developing the new virus-based therapeutic strategy

to treat GBM is clinically relevant. However, the mechanistic action of RRV-RLI treatment as defined in the current study is largely descriptive.

Comment #1: Fig. 1G, H: The proportion of cell types in the Alluvial plot (Fig. 1H) does not clearly match those presented in the UMAP plots (Fig. 1G), and what is the cellular identity of APC?

Response #1: We thank the reviewer for their comment. Regarding differences in proportions of cell types between **Figure 1G and H**, the UMAP in **Figure 1G** is a pooled 2-D visualization which can be distorted by point overlap and local crowding. As such, **Figure 1G** might not perfectly match the proportions in **Figure 1H**, which instead reports per-sample cell counts normalized to the total number of cells and is more quantitative in nature. To further clarify this, we have added a note to the methods that proportions in the alluvial plot were computed from per-sample counts (Page 18, Lines 795-796).

Regarding the cellular identify of APC, this label represents antigen-presenting cells as defined by singleR unsupervised cell labeling and CIITA, CD74, and CD86 expression.[10] We have clarified the definition of APCs in the **Figure 1** legend and specify in the methods section that "subclassification of immune cells was achieved using labels suggested by SingleR and marker gene expression identified through literature review" (Page 18, Line 788-790).

Comment #2: Fig. 2, Supplementary Fig.1, Supplementary Fig. 2A and throughout the manuscript: It is not clear if the secreted RLI is a true receptor-linked–IL-15, or just soluble IL-15. Furthermore, it is not clear if the RRV-RLI treatment effects result from the secreted receptor-linked–IL-15, or just soluble IL-15.

Response #2: We thank the reviewer for raising this clarifying point. The RRV-RLI vector only encodes receptor-linked-IL-15 not soluble IL-15. This is human IL-15 that is fused to an IL-15 receptor alpha sushi domain using a flexible linker sequence that has been previously validated in the literature as producing receptor-linked-IL-15 that does not degrade into soluble IL-15.[11] Our vector therefore does not express IL-15 alone and therefore only leads to the secretion of receptor-linked-IL-15, which does not ultimately give rise to soluble IL-15. We have confirmed our vector sequence using Sanger sequencing. We have also demonstrated stability of our vector transgene at the expected size for the RLI coding sequence on PCR (**Supplemental Figure 1C**) after over 8 weeks of continuous vector replication and spread in vitro. The PCR primers we utilize span the entire RLI construct, including the IL-15 and IL-15 receptor linker region, confirming a fusion-specific amplicon. We demonstrated the functionality of our RLI construct in **Figure 2F-I**. To further confirm that our vector is specifically delivering and leading to the production of RLI rather than soluble IL-15, we have also confirmed sequence-specific integration into tumor cells *in vivo* 10 days after infection using a virus-specific qPCR that is only specific to our viral genome sequence (**Supplementary Figure 2A**, Page 6, Lines 179-183). We believe that altogether, our findings support that the treatment effects seen with RRV-RLI can be attributed to the RLI fusion protein, not soluble IL-15. We have added these findings to the results section for additional clarity, and appreciate the reviewer bringing up this point.

Of note and somewhat beyond the question of which form of IL-15 was delivered, coordinately performed ELISA also confirmed that the RLI produced after *in vivo* expression was detectable by an anti-IL-15 antibody capable of detecting the IL-15 moiety within RLI (**Supplementary Figure 2B**, Page 6, Lines 179-183) that correlated with RRV-RLI viral infection with no evidence of IL-15 or RLI protein in PBS-treated mice, underscoring how low levels of IL-15 in murine gliomas made them well suited for RRV-RLI treatment, similar to the findings from the TCGA data in **Figure 1** across multiple cancers.

Comment #3: Fig. 2F.G: It is not clear about what is the "untreated" group. Are these mice age-matched to the re-challenged mice?

Response #3: We thank the reviewer for their comment. Yes, the mice in the untreated group are tumor-bearing mice that are age-matched to the rechallenged mice and were not treated. We have added additional text in the results and methods to clarify this point (Page 6, Lines 177-178 and Page 15, Lines 656-657).

Comment #4: Fig. 4: The analysis based on the comparison of RRV-RLI group to the PBS group cannot clearly distinguish the immune changes as a direct result of anti-RRV or RLI or anti-tumor effect.

Response #4: We thank the reviewer for their insight. Prior work[4] and our own data demonstrate that RRV infection alone does not significantly impact tumor growth, survival, or immune infiltration compared to PBS controls, and both differ significantly from RRV-RLI. We do not observe any survival benefit associated with RRV treatment (**Figure 3B-E**). We addressed this comment when, transcriptomically, we showed RRV vs. RRV-RLI transcriptomic immune-infiltration comparison as a heat map in **Supplementary Figure 2H,** a heat map demonstrating differences in gene expression between RRV and RRV-RLI in **Supplementary Figure 2G,** a volcano plot demonstrating differential gene expression between RRV-RLI and RRV in **Supplementary Figure 2F,** and a figure demonstrating differences in gene pathways between RRV and RRV-RLI in **Supplementary Figure 2E**. **Supplementary Figure 2H** also includes mice at endpoint who progressed after initial RRV-RLI response, revealing that progression after initial response was associated with reversion of the immune microenvironment to an immunologically "cold" state with low T and NK cell infiltration, similar to results seen with RRV. In addition, co-culture experiments with T and NK cells show no increase in activation or proliferation with RRV or untreated tumor cells (**Figure 2G-I, Supplementary Figure 1D-H**), but show a large increase in activation and proliferation with RRV-RLI treated cells, implicating RLI, not RRV, in the biological changes seen after RRV-RLI treatment.

To further address the reviewer's excellent point, after discussion with the editor, and to further assess the impact of RRV on the immune microenvironment, we completed additional *in vivo* experiments where we directly compared immune infiltration in mice treated with RRV-RLI vs. RRV at a matched timepoint via flow cytometry. This flow cytometric analysis demonstrates significantly higher infiltration of $CD3^+$ T cells, $CD8^+$ T cells, NKT cells, and NK cells in RRV-RLI treatment groups relative to both PBS and RRV treatment groups, with no significant difference between PBS and RRV treatments (**Figure 6I-M**). Similarly, RRV-RLI results in a proportional decrease in infiltrating $CD11b^+$ cells relative to RRV and PBS treatments with no difference between RRV and PBS groups (**Figure 6N**). We have amended the results section (Page 8, Lines 293-311) and **Figure 6** to reflect these findings. In summary, transcriptomic (**Supplementary Figure 2E-H**), flow cytometric (**Figure 6I-N**), and survival data (**Figure 3B-E**) demonstrate no significant difference between PBS and RRV treatment groups, whereas RRV-RLI demonstrated striking differences from both. In addition, in our single cell TCR sequencing data we do not see TCRs that are targeted against the MLV envelope and only 2.3% of TCRs were within 18 distance units of a pathogen-specific TCR (**Figure 7H** and Page 10, lines 403-405). These data support that the immune modulation and therapeutic benefit of RRV-RLI is due to the RLI payload rather than the RRV vector backbone. We have added additional reasoning around this topic to the discussion section (Page 11-13, Lines 475-534).

.

Comment #5: Fig. 4F: Why exhausted CD8 score is increased in the RRV-RLI group vs PBS? Again, Supplementary Fig. 2E: why exhausted CD8 is higher in RRV-RLI TP vs RRV-RLI EP or RRV TP?

Response #5: Regarding the higher "exhausted" CD8 score in the RRV-RLI treated group as compared to the PBS treatment groups, we believe this due to multiple factors. First, there is extremely low T cell infiltration in PBS and RRV treated SB28 mice, as can be seen in our flow cytometric analysis (**Figure 6**, <5% infiltration for $CD3^+$, $CD4^+$, and $CD8^+$ T cell populations in PBS and RRV treated SB28 tumors), which is consistent with the literature for SB28[7,12] and part of the reason that SB28 is seen as a very immunologically "cold" model that recapitulates human GBM. As such, the increase in "exhausted" $CD8^+$ score on transcriptomic analysis is likely reflective of an absolute change in the number of $CD8^+$ T cells with RRV-RLI treatment rather than an increase in the relative ratio of exhausted and non-exhausted CD8 T cell populations. Indeed, when we assess the ratio of exhausted to non-exhausted $CD8^+$ T cells, the ratio in the RRV-RLI treatment is more favorable than that observed in the control group (1.2 vs. 0.84, p=0.04, Student's t-test). Second, a higher exhausted $CD8^+$ score in the RRV-RLI treatment group is, in many ways, expected with antigen-driven activation. In antigen-driven activation, activated $CD8^+$ T cells can upregulate markers such as PD1, which are markers of both activation and exhaustion.[3] The upregulation of these markers may contribute to a higher "exhausted" $CD8^+$ T cell score, but, in fact, is likely representative of activation. This is supported by the 5' single-cell RNA sequencing data (**Figure 7C**), which examines PBS and RRV-RLI-treated mice. In the scRNA sequencing data, we observe a small population of "exhausted" $CD8^+$ T cells, but much larger populations of $CD8^+$ effector cells, early activated T cells, and NKT cells.

We also performed additional *in vitro* experiments to directly evaluate the impact of RLI exposure on T cell exhaustion markers. In these experiments, we observe an increase in expression of activation marker CD69 with concordant expression of PD-1 likely more reflective of it as an activation marker rather than its role as an exhaustion marker because we observed no significant expression of any other exhaustion marker (TIM3, LAG3, CTLA4) and also found no concordant expression of these other exhaustion markers alongside PD-1.[3] We have amended the results to reflect these findings. (**Supplementary Fig. 1I**, Page 5, Lines 156-162).

We believe a similar rationale applies to **Supplementary Fig. 2E**, primarily that an increased "exhausted" CD8 T cell score is primarily indicative of increased overall T cell infiltration and antigen driven activation in RLI-RRV treated mice at timepoint, during high levels of immune activation, as compared to empty RRV at timepoint (TP) or RRV-RLI treated mice that have escaped treatment at endpoint (EP). We have added additional clarification around this topic to the discussion section (Page 11-12, Lines 466-470).

In summary, through leveraging transcriptomic ratios, our scRNA sequencing data, and additional *in vitro* experiments, we believe that the increased "exhaustion score" is in fact representative of increased infiltration and activation rather than true exhaustion

Comment #6: Fig. 5: The tumor volume and mouse survival in the RRV-RLI + isotype control group need to be compared to those in the RRV-RLI + anti-CD4/CD8 group and those in the RRV-RLI + anti-CD8 group. Additionally, the tumor volume and mouse survival in the PBS + isotype control group need to be compared to those in the PBS + anti-CD4/CD8 group and those in the PBS + anti-CD8 group.

Response #6: We thank the reviewer for their comment. We have added additional depletion experiments, including both CD4 and NK cell depletion alone, as well as a repeat of the CD8 depletion with a larger sample size. As the reviewer asked, we have compared all the RRV-RLI treatment groups across experiments. In this analysis, only the combined CD4 and CD8 depletion (where RRV-RLI efficacy was completely abrogated) significantly differed from the RRV-RLI

treatment groups (**Supplementary Figure 3E**, Page 7, Lines 251-253). When comparing the PBS treatment groups, we observed minor differences between isotype and combined CD4 and CD8 depletion as well as isotype and NK depletion. Median survival in the PBS treatment groups across depletion experiments ranged from 15 to 20 days. Given the small difference in survival between the PBS groups we do not consider them biologically significant but have added the data to the results section (Page 7, Lines 251-253) and **Supplementary Figure 3D**. In summary, our data reveal that only combined CD4 and CD8 depletion completely abrogates the RRV-RLI treatment response.

Comment #7: Fig. 4 and Supplementary Fig. 2 indicate that NK cell response is increased in the RRV-RL1 group. Expansion of NK cells is pronounced in RRV-RL1 +/-TMZ group (vs PBS +/-TMZ group) and NK cells are the most abundant in these 2 groups, as shown in Fig. 7C. The contribution of NK cells to tumor progression and mice survival upon RRV-RL1 treatment +/-TMZ needs to be experimentally evaluated.

Response #7: We thank the reviewer for their comment and agree with their rationale. In response to this point, we did complete an *in vivo* NK cell depletion experiment to examine the impact of NK cells on RRV-RLI therapeutic efficacy. These data demonstrated that NK depletion did not abolish the survival benefit seen with RRV-RLI treatment (median survival 42.5 days with RRV-RLI vs. 20.0 days with intratumoral PBS treatment in NK-depleted mice, $p<0.001$, Log-Rank Mantel-Cox, **Figure 5I and J**) and was not statistically significantly different than RLI-RRV + Isotype control ($p>0.05$, Log-Rank Mantel-Cox, **Supplementary Figure 3E**). However, combined CD4 and CD8 depletion abrogated RLI-RRV survival and CD8 depletion alone did not **Figure 5B-E**. This suggests that, while NK cells may contribute to the survival benefit seen with RRV-RLI treatment, NK cells are not necessary for this survival benefit. Instead, $CD8^+$ and $CD4^+$ T cells coordinate to serve as the primary co-drivers of RRV-RLI therapeutic efficacy, with both together needed for this survival benefit in a manner that the therapeutic benefit can continue as long as one of these T-cell subtypes is present, but is lost once both are gone, a versatility that has implications for the translatability of this approach where patients could derive a benefit unless they had a dramatic reduction in levels of both T-cell subtypes. We have added the NK cell depletion to the results section (Page 7, Lines 249-253) and have added additional points around this topic to the discussion (Page 12, Lines 483-487).

Comment #8: Supplementary Fig. 5B,C: Use of Tox as the only gene indicating CD8 or NKT exhaustion is not sufficient.

Response #8: We thank the reviewer for this comment. In **Supplementary Figure 5B and C**, our intent was not to comment on levels of T cell exhaustion between lymphocytes from RRV-RLI+TMZ vs. RRV-RLI groups, but to highlight that, while there weren't many gene expression changes in the lymphocytes from these two groups, among the most significant changes were that adding TMZ to RRV-RLI led to increased lymphocyte granzyme expression and decreased lymphocyte *Tox* expression, both of which would be consistent with a more activated T-cel phenotype as granzymes are associated with increased activation of NK cells and $CD8^+$ T-cells, while *Tox* is an HMG-box transcription factor that serves as a central regulator that can transcriptionally and epigenetically program CD8+ T-cell exhaustion such that *Tox* downregulation as seen with addition of TMZ to RRV-RLI is associated with increased T-cell activation.[13,14] We have amended the discussion to clarify that we are describing overall transcriptional tendencies rather than classifying cell states (Page 12, Lines 510-512).

Comment #9: The results showing that increased osteopontin (Spp1) from myeloid/macrophages may enhance immune activation in RRV-RL1 +/-TMZ groups (Fig. 7G and Supplementary Fig.

5G) are contradictory to the report that myeloid-derived Spp1 drives CD8+ T-cell exhaustion (PMID: 36638785).

Response #9: We thank the reviewer for this important point on the mixed literature around SPP1 (osteopontin). We agree that SPP1 has been shown to be involved in some immunosuppressive mechanisms, especially in binding CD44. At the same time, loss of SPP1 has been shown to be pro-tumorigenic and SPP1 is a well-described marker of GBM-associated macrophages in general.[15] In our dataset, we also see inferred binding of SPP1 to T cell integrins (ITGA4_ITGB1 and ITGAV_ITGB1), which may also reflect an association with tissue remodeling and immune migration.[16] The context-dependent nature of SPP1 expression is also supported by the co-occurrence of increased antigen presentation pathways in some of the cell types with SPP1 expression. We appreciate the reviewer in bringing up this point and have highlighted the nuance around SPP1 by amending the text to mention that SPP1 is a glioma-associated macrophage marker that may be representative of counter-regulatory immunosuppressive or immune migratory pathways, and citing the corresponding literature (Page 10, Lines 373-375).

Comment #10: Increased cross-presentation to CD8+ T-cells by myeloid cells in RRV-RL1 +/- TMZ groups is inferred from the CellChat analysis. A direct experiment proving this is missing.

Response #10: We thank the reviewer for this comment. Our conclusion of increased cross-presentation in the RRV-RLI +/- TMZ groups is based on the CellChat interactions, the broad increase in MHC Class I protein expression in myeloid populations (**Figure 7F**), and the increased number of recurrent T cell receptors in the RRV-RLI + TMZ group as compared to RRV-RLI + Vehicle. Interestingly, this pattern is consistent with prior TMZ literature, which demonstrates that TMZ can improve cross-presentation as well as augment tumor-specific T-cell responses in patients and preclinical models.[17–19] In addition, IL-15 is well known to support antigen presentation pathways and antigen presenting cell function.[20–22]

We agree with the reviewer that a direct experiment was needed to support the increase in antigen presence after treatment with TMZ. To further clarify the role of TMZ in releasing tumor antigens that lead to enhanced antigen presentation, we have performed a functional cross-presentation assay. In this experiment, we utilize SB28 tumor cells expressing ovalbumin (OVA) and assess the ability of dendritic cells pulsed with their supernatant to activate OT-1 CD8 T cells (which recognize OVA SIINFEKL peptide presented by the MHC class I). We compare OT-1 activation between SB28-OVA cells that were treated with TMZ and those without TMZ treatment. In SB28- OVA cells treated with TMZ, we see an increase in downstream OT-1 T cell activation (as evidenced by $CD69^{+}$, 13.8% vs. 8.8%, $p<0.05$, Welch's t-test, **Supplementary Figure 6A**) and cell proliferation (as evidenced by $Ki67^{+}$, 11.7% vs. 7.2%, $p<0.05$, Welch's t-test, **Supplementary Figure 6B**). These data further support a model where TMZ cell death increases antigen supply, which then synergizes with RRV-RLI enhancement of T cell, NK cell, and antigen presentation responses. We have amended the results section (Page 10, Line 376-378), **Supplementary Figure 6A-B,** and the discussion (Page 13, Line 527-529) to reflect these findings.

Comment #11: The tumor-reactivity of T-cells and the lack of immunogenicity of the RRV backbone are inferred by TCR analysis. A direct experimental strategy proving the tumor-reactivity without the RRV reactivity of expanded T-cells in RRV-RL1 +/-TMZ groups is required.

Response #11: We thank the reviewer for their astute comment. Given the extremely low T cell infiltration in our SB28 model (<5% in PBS or RRV-treated mice) and the technical challenge of tumor-infiltrating lymphocyte isolation, we cannot reliably recover sufficient T cells for *ex vivo* antigen-specific assays. As such, we infer the tumor-specificity rather than RRV-specificity of the

immune response after RRV-RLI treatment through multiple convergent datasets. First, as detailed in **Response 4**, using co-culture experiments (**Figure 2G-I, Supplementary Figure 1D-H**), *in vivo* transcriptomic (**Supplementary Figure 2E-H**), *in vivo* flow cytometric (**Figure 6I-N**), and survival data (**Figure 3B-E**) we do not observe a significant difference between PBS and RRV treatment groups, whereas RRV-RLI demonstrated differences from both. This data from our work and previous work[4] suggest that the RRV backbone is relatively immunologically inert and isolating the biological changes seen to the RLI payload. Second, the cross-presentation experiment detailed in **Response 9** demonstrates an increase in OT-1 T cell activation and proliferation after TMZ treatment that is specific to a tumor-antigen (in this case OVA), supporting the ability for TMZ treatment to amplify a tumor antigen-specific response. This is further supported by our single-cell RNA sequencing data, which demonstrates a broad increase in antigen presentation machinery and recurrent TCRs in the RRV-RLI + TMZ condition. Third, our single-cell beta chain TCR analysis not only demonstrates no matches to the envelope protein of MLV, but none of the TCRs reactive to the envelope protein of MLV were within 100 distance units for paired chains or 18 distance units for beta chains in our samples (Page 10, Lines 399-405). Only 2.3% (42/1813) of beta chain TCRs were within 18 distance units of a pathogen-specific TCRs from McPAS-TCR. In the revised manuscript, we also performed an additional paired alpha-beta chain analysis. In this analysis, we have identified 1/1364 exact alpha-beta matches to a pathogen in McPAS (influenza) and 35/1364 alpha-beta pairs within a 100-unit distance of McPAS pathogen antigens. We have added these new findings to the results section (Page 10, Lines 411-412). Finally, our Tu2449 rechallenge data, where immunologic memory was observed against untreated tumors in the Tu2449 model, also supports a tumor specific response.

Comment #12: Supplementary 1F: It is not clear if NK cells or CD8+ T-cells are analyzed here based on the legend.

Response #12: We thank the reviewer for pointing this out. There was a typographical error in the original figure legend that has now been corrected. **Supplementary Figure 1F** (now **Supplementary Figure 1E** after revisions) is in reference to $CD8^+$ T cells, and we have addended the figure legend to reflect this.

<u>Reviewer #2's 2nd Set of Comments:</u>

The revised manuscript has largely addressed the prior concerns. However, a few minor issues still need attention:

Comment #1: The line numbers cited in the response/rebuttal do not match those in the revised manuscript.

Response #1: We thank the reviewer for their comment. We have made sure that the line numbers in the rebuttal match those in the manuscript.

Comment #2: Some figures appear to be cited incorrectly in the response/rebuttal (e.g., The cross-presentation assay was shown in supplementary Fig. 6A,B, not in Supplementary Fig. 5H,I).

Response #2: We thank the reviewer for their comment. We have made sure that the figures cited in the rebuttal text above match those in the manuscript.

Comment #3: The references in the response/rebuttal appears out of order. For example, ref 10 (PMID: 25972484) is cited for “this label represents antigen-presenting cells as defined by singleR unsupervised cell labeling and CIITA, CD74, and CD86 expression”; ref 11 (PMID: 19889936) is cited for “…validated in the literature as producing receptor-linked-IL-15 that does not degrade into soluble IL-15”; ref 4 (PMID: 11702064) is cited for “prior work” related to “RRV infection alone does not significantly impact tumor growth…”
In addition, while 25 references are cited, only 21 are listed. Please ensure all cited references are included and correctly ordered.

Overall, the reviewer would appreciate a thorough cross-check of both the revised manuscript and the response/rebuttal to ensure internal consistency throughout.

Response #3: We thank the reviewer for their comment. We have made sure that the references in the response letter are updated.

Comment #4: Fig. 1 G,H legend: the prior comment was not requesting a definition of APC; rather, it asked what cell type represents APC (e.g., macrophage or DC). Please clarify this explicitly in the legend/text.

Response #4: We thank the reviewer for their comment. We defined the APC population singleR unsupervised cell labeling and CIITA, CD74, and CD86 expression. As such, it represents a mixed population of myeloid cells, including macrophage/dendritic cell populations. We have updated the **Figure 1** legend to reflect this.

<u>Reviewer #3’s Comments:</u>

The authors have a strong track record in clinical trials of replicating retroviral vectors (RRV) for glioblastoma and have continued to develop these vectors for more effective patient treatment. Despite promising results, Phase III trials revealed that the suicide gene (CD) was insufficient to kill glioma cells and stimulate long-term anti-tumor immunity. In this manuscript, the authors evaluate a new RRV armed with cDNA encoding a superagonist form of IL-15 (RLI), a cytokine that stimulates NK and $CD8^+$ T cells. Using murine glioma cell lines SB28 and Tu2449, they provide evidence of the efficacy of this new virus, RRV-RLI, including profiling immune cell populations and TCR repertoires using the 10x platform.

Comment #1: There are some concerns in experimental setting; the lack of a control virus in TIL analysis, particularly in Figures 4 and 7, limits the ability to determine the IL-15 specific effects.

Response #1: We thank the reviewer for this insightful comment. Regarding the lack of control RRV virus for TIL analysis, we have completed additional *in vivo* experiments where we directly compare immune infiltration in mice treated with RRV vs. RRV-RLI at a matched timepoint via flow cytometry. This flow cytometric analysis demonstrates significantly higher infiltration of $CD3^+$ T cells, $CD8^+$ T cells, NKT cells, and NK cells in RRV-RLI treatment groups relative to both PBS and RRV treatment groups, with no significant difference between PBS and RRV treatments (**Figure 6I-M**). Similarly, RRV-RLI results in a proportional decrease in infiltrating $CD11b^+$ cells

relative to RRV and PBS treatments with no difference between RRV and PBS groups (**Figure 6N**). We have amended the results section (Page 8, Line 293-311) and **Figure 6** to reflect these findings.

Regarding empty RRV as a control in addition to intratumoral PBS in other experiments (e.g. depletion studies, combination with TMZ, combination with anti-PD1 treatment, use of lower virus dose, and delayed viral injection) that came up as a comment from multiple reviewers, we discussed the use of PBS as a control group for these studies with the editor who, noting the lack of survival benefit with RRV treatment, suggested that further immune profiling of RRV tumors, which was the focus of this particular comment, would be more informative than use of RRV as a control group in these other studies. Prior work[4] and our own data demonstrate that RRV infection alone does not significantly impact tumor growth, survival, or immune infiltration compared to PBS controls, and both differ significantly from RRV-RLI. We do not observe any survival benefit associated with RRV treatment (**Figure 3B-E**). Transcriptomically, we show: (1) a figure demonstrating differences in immunologic gene pathways after RRV-RLI vs. RRV treatment at timepoint (14 days after tumor implantation, 10 days after viral implantation) (**Supplementary Figure 2E)**; (2) a volcano plot demonstrating differential gene expression at timepoint after RRV-RLI vs. RRV treatment (**Supplementary Figure 2F)**: (3) a heat map demonstrating differences in gene expression after RRV-RLI vs. RRV treatment at timepoint (**Supplementary Figure 2G)**; and (4) transcriptomic immune-infiltration comparison as a heat map (**Supplementary Figure 2H)** comparing tumors receiving RRV at a timepoint to tumors receiving RRV-RLI at timepoint and at endpoint when the tumor has progressed to become fatal after initial response, which revealed reversion of the immune microenvironment to an immunologically "cold" state with low T and NK cell infiltration in the setting of progression after initially successful RRV-RLI treatment, similar to the response to RRV. In addition, co-culture experiments with both T and NK cells show no increase in activation or proliferation with RRV or untreated tumor cells (**Figure 2G-I, Supplementary Figure 1D-H**), but show a large increase in activation and proliferation with RRV-RLI treated cells, isolating the biological changes seen to the RLI payload. Furthermore, as eventual treatment in patients would be RRV-RLI rather than RLI alone, we highlighted the comparison between PBS and RRV-RLI as we believe this to be the most translationally relevant comparison. In addition, in our single-cell TCR sequencing data we do not see TCRs that are targeted against the MLV envelope and only 2.3% of TCRs were within 18 distance units of a pathogen-specific TCR (**Figure 7H** and Page 10, Lines 399-412). These data support that the immune modulation and therapeutic benefit of RRV-RLI is due to the RLI payload rather than the RRV vector backbone. We have added additional reasoning around this topic of RRV as a control to the discussion section (Page 12, Lines 475-480).

Comment #2: Additionally, in vivo treatment starting at day 4 post-tumor may coincide with scar healing from needle injection, raising questions about whether RRV-RLI infects parenchymal and innate cells for persistent IL-15 production rather than targeting the tumor itself. This stage mainly recruits NK cells, making RRV-RLI more effective at stimulating NK cells while adaptive immune cells typically start infiltrating at day 6-7 in this model. This timing may eliminate seed tumors before generating a tumor microenvironment. Discussing these points would be appreciated, considering RRV's naturally non-lytic feature.

Response #2: We thank the reviewer for their comment. To address this concern, we have performed additional mouse studies. First, just as the reviewer suggested, we completed another survival experiment with RRV-RLI, but included a group that was dosed at day 7 post-tumor implantation. We found that administration of RRV-RLI at day 7 still prolonged survival compared to PBS control (median survival 17 days vs. 21 days, $p<0.05$). While there was a trend towards reduced survival in the RRV-RLI day 7 injection as compared to RRV-RLI day 4, the difference did not quite reach significance (median survival 21 days vs. 50.5 days, $p=0.064$, **Supplementary**

Figure 3J). This appeared consistent with a kinetic constraint such that delaying therapy provided less time for vector spread and RLI accumulation in the fast-growing SB28 model (control average median survival ~17 days).

In addition, we completed a slightly different experiment along this same theme of creating a more biomimetic scenario of a less favorable ratio of viral particles to tumor cells at the time of implantation, when we reduced the viral dose 10-fold ($2.5x10^4$ TU, **Supplementary Figure 3K**), leading to maintenance of therapeutic efficacy relative to PBS control (median survival 18 days vs. 31 days, $p<0.01$), albeit to a lesser degree than the efficacy seen at the 10-fold higher dose (median survival 18 days vs. 56 days, $p<0.001$), again suggesting a dose/kinetic impact on therapeutic efficacy. Finally, we don't believe we are eliminating seed tumors that haven't fully established as, on bioluminescent imaging, RRV-RLI treated tumors frequently grow similarly to control until approximately 10-14 days after implantation (**Figure 3B-E**). We have added these findings to the results section (Page 8, Lines 273-279) as well as additional information around this topic in our discussion (Page 12, Line 496-498)

We also appreciate the reviewer's points regarding off-target infectivity and early NK cell activation as a primary driver of therapeutic efficacy. We believe that several lines of evidence make this less likely. First, RRV is a γ-retrovirus that requires actively dividing cells and mitosis in order to infect, stably integrate its payload, and spread due to the lack of nuclear localization sequence in the RRV genome. This makes normal brain parenchyma or scar tissue after tumor implantaton a less favorable target given its largely post-mitotic nature.[23] To further address this topic and confirm that RRV-RLI specifically replicated in dividing cells which are limited to the tumor in the brain, we have assessed brain parenchyma contralateral to RRV-RLI injected tumors at tumor endpoint. Even with high viral copies/ng of DNA in the tumor, we do not see significant evidence of viral infectivity in contralateral brain tissue, suggesting that the virus is unable to replicate and spread effectively in normal brain parenchyma (**Supplementary Figure 2D**). Second, we have performed an NK cell depletion experiment which did not abrogate the therapeutic response to RRV-RLI. This finding, combined with the complete abrogation in combined CD4 and CD8 depletion, suggests that the primary driver of the therapeutic benefit of RRV-RLI is the adaptive immune system (**Figure 5**).

Comment #3 (three points related to Figure 1):

- For Figure 1 analysis, the manuscript is missing details on patient samples, data, analysis methods, and the IRB statement.
- Was tumor size considered when comparing IL-15 high vs. IL-15 low GBM tumors, especially since IL-15 high tumors have fewer tumor cells?
- Were the resected tumor samples taken from the tumor core or edges? The sampling location can affect the analysis of tumor-infiltrating cells.

Response #3: **Figure 1** analyses were performed on external, publicly available tumor datasets.[24,25] No new human samples were collected for our study, eliminating the need for IRB approval for our work. Information regarding specifics around data analysis methods can also be found in the methods section under the headers: *Human bulk RNA-sequencing analysis* and *Human scRNA and immune cell profiling analysis*. We do not have access to the spatial distribution of collected samples for bulk RNA-sequencing. For the scRNA sequencing data, cells were taken from the core and edge. There was a comparable percent of core cells in the IL-15 high and low groups (63.6% vs. 66.6%, p=0.66, Page 4, Lines 100-106), and for the purposes of our analyses, we did not distinguish between core vs. edge. There is no available information on tumor size for these data sets. We have added additional clarification around these points to the methods section (Page 17-18, Line 766, 793-795).

Comment #4: The authors demonstrated that co-culturing T- and NK cells with RRV-RLI promotes their expansion and leads to their activation, as indicated by increased CD69 and Granzyme B expression. Given that RRV is known to infect immune cells and use them as carriers, culturing T cells with the virus in the absence of tumor cells could help determine whether the observed effects are due to direct viral influence on T cells rather than IL-15 secreted by infected tumor cells (i.e, direct viral infection to T cells in co-culture and its impact on T cell function and proliferation)

Response #4: We thank the reviewer for this helpful comment. To directly address the reviewer's point about direct viral infection of T cells in co-culture, we have added RRV-RLI directly to isolated T cells without tumor cells. In this treatment group (**Figure 2G, Supplementary Figure 1D**), we do not see a significant change in activation or proliferation marker expression between T cells incubated with RRV-RLI, T cells alone, T cells exposed to RRV-infected tumor cells, or T cells exposed to uninfected tumor cells. In contrast, T cells exposed to RRV-RLI infected tumors demonstrated significantly higher activation and proliferation, isolating T cell changes to the RLI payload. We believe this indicates that the activation and proliferation of immune cells seen in the co-culture experiments is primarily related to RLI secretion by infected tumor cells. We have added these findings to the results section (Page 5, Lines 161-162). Also, of relevance to this point, we should also mention that, in experiments assessing the activation of T cells and NK cells in response to RRV-RLI, the immune cells were co-cultured with tumor cells that were either uninfected, infected by RRV (empty virus), or infected by RRV-RLI. Given the replicating nature of RRVs, tumor cells infected with RRV or RRV-RLI are continuously producing their respective virus (in addition to RLI, in the case of RRV-RLI infected tumors). As such, T. cells or NK cells in the RRV co-culture setting are exposed to circulating RRV viral particles in the cell media. The lack of increased immune cell activation or proliferation in the RRV alone group relative to PBS suggests that possible RRV infectivity of T-cells or NK-cells is unlikely to have contributed to changes in immune cell function and proliferation seen during co-culture of these immune cells with RRV-RLI-infected tumor. Cells.

Comment #5: Considering that tumor-infiltrating myeloid cells or microglia may infiltrate in response to the virus and potentially limit viral replication, this could reduce IL-15 secretion and contribute to T cell exhaustion near the study endpoint. How long does IL-15 agonist secretion from viruses persist in vivo? It is suggested to analyze native IL-15 gene and RLI transcripts obtained from RNA-seq data (e.g., Fig 4) or through IHC and RT-PCR at analysis points.

Response #5: We thank the reviewer for this helpful comment. As suggested, we have completed an additional *in vivo* mouse study where we assessed viral genome presence, RLI transgene presence, and RLI protein expression in a timepoint experiment (14 days after tumor implantation, 10 days after viral delivery), which is consistent with our timepoints used for tumor microenvironment analysis. At this timepoint, RRV-RLI-infected tumors exhibited robust viral genome presence (mean viral genome/ng gDNA 13.25 RRV-RLI vs. 0 in PBS control, **Supplementary Figure 2A**) and RLI protein expression (mean RLI/IL-15 pg/mL 381 in RRV-RLI vs. 0 in PBS control **Supplementary Figure 2B**). We have added these findings to the results section (Page 6, Lines 179-183).

Comment #6: In Figure 4D, the heatmap shows increased expression of immune checkpoint inhibitors like CTLA-4, PD-1, and TIGIT in the treatment group, and Figure 4F shows an increased immune score for exhausted CD8 T cells. Results and discussion lack this data interpretation. Could the T cells be exhausted due to an immunosuppressive environment promoted by the virus or tumor factors? Did the authors assess if immunosuppressive cells like TAMs or MDSCs

increased with virus infection? Were other cytokines like IFN-γ or IL-10 assessed in the tumor microenvironment or in vitro, as they might contribute to T cell exhaustion? Probably, it would be appreciated to add more discussion.

Response #6: We thank the reviewer for their comment. Regarding the higher “exhausted” CD8 score in the RRV-RLI treated group as compared to the PBS treatment groups, we believe this due to multiple factors. First, there is extremely low T cell infiltration in PBS and RRV treated SB28 mice, as can be seen in our flow cytometric analysis (**Figure 6**, <5% infiltration for $CD3^+$, $CD4^+$, and $CD8^+$ T cell populations in PBS and RRV treated SB28 tumors). This is consistent with the literature for SB28[7,12] and part of the reason that SB28 is seen as a very immunologically “cold” model that recapitulates human GBM. As such, the increase in “exhausted” CD8 score on transcriptomic analysis is likely reflective of an absolute change in the number of CD8 T cells rather than an increase in the relative ratio of exhausted and non-exhausted CD8 T cell populations. Indeed, when we assess the ratio of exhausted to non-exhausted CD8 T cells, the ratio in the RRV-RLI treatment is more favorable than that observed in the control group (1.2 vs. 0.84, p=0.04, Student’s t-test, Page 7, Lines 217-219). Second, a higher exhausted CD8 score in the RRV-RLI treatment group is in many ways expected with antigen-drive activation. In antigen driven activation, activated $CD8^+$ T cells can upregulate markers such as PD1, which are markers of both activation and exhaustion.[3] The upregulation of markers like PD1 may contribute to a higher “exhausted” $CD8^+$ T cell score, but in fact be representative of activation. This is supported by the 5’ single cell RNA sequencing data (**Figure 7C**), which examines PBS and RRV-RLI treated mice. In the scRNA sequencing data we observe a small population of “exhausted” $CD8^+$ T cells, but much larger populations of $CD8^+$ effector cells, early activated T cells, and NKT cells. We also observed a decrease in MDSC-like neutrophil populations (**Supplementary Fig. 5F,** Page 9, Lines 358-361). To investigate T-cell exhaustion further, we performed additional culture experiments directly evaluating the impact of RLI exposure on T cell exhaustion markers. In these experiments, we observe an increase in the activation marker CD69 alongside expression of PD-1, which in this context is likely an activation marker rather than an exhaustion marker as it occurred without significant concordant single or multi-expression of other exhaustion markers (TIM3, LAG3, CTLA4). We have amended the results to reflect these findings. (**Supplementary Fig. 1I**, Page 5, Lines 156-162).

Comment #7: RRV-RLI monotherapy significantly improved survival in the Tu2449 GBM model, with 90% of mice showing long-term survival and developing immunologic memory. However, this effect was not observed in the SB28 model. What factors or aspects of the tumor microenvironment contribute to the different outcomes in SB28 and Tu2449? Could this difference be due to variations in infectivity or replication of the virus in these models?

Response #7: We thank the reviewer for their comment and astute points. We believe the primary driver of differences in tumor response and survival are related to the underlying immunogenicity of each model. While Tu2449 is also poorly immunogenic relative to most murine GBM models and has been used frequently in the literature, it does exhibit higher T-cell infiltration compared to the SB28 model, indicating increased relative immunogenicity. SB28 is one of the least immunogenic models available and is characterized by low T cell infiltration and a low tumor mutational burden, similar to human GBM.[7,12] While differences in viral replication and spread are certainly possible, we think this is less likely, as viral replication was similar between the two models during head-to-head comparisons in culture (**Figure 2C and D**). We have clarify this topic in our discussion section (Page 11, Lines 457-462)

Comment #8: The TCR analysis concludes that the identified TCRs represent a tumor-specific response enhanced by RRV-RLI and RRV-RLI+TMZ rather than an antiviral response to RRV-

RLI. However, this statement needs supportive evidence and might be better placed in the discussion section. Using scRNA with TCR profiling to identify matching alpha-beta chains at the single-cell level would support this claim by addressing specific antigen-responsive T cells with reconstituted TCR analysis.

Response #8: We thank the reviewer for this comment. In the results section of the original manuscript, we focused on the beta-chain TCR repertoire from our 5' single cell RNA sequencing experiment. This showed minimal overlap with pathogen-reactive TCRs in McPAS. In response to the reviewer's astute comment, we have also performed a paired alpha-beta analysis for the revised manuscript. In this analysis, we have identified 1/1364 exact alpha-beta matches to a pathogen in McPAS (influenza) and 35/1364 alpha-beta pairs within a 100-unit distance of McPAS pathogen antigens. We have added this to the text (Page 10, Lines 411-412). In addition, as discussed in **Responses 1 and 4,** using multiple orthogonal modalities, we do not observe a significant immune reaction to RRV alone. In addition, the immunologic memory observed to untreated tumors cells in the Tu2449 rechallenge experiment suggests a tumor specific immune response, rather than an anti-viral immune reponse. Altogether, we believe that the greater clonal expansion with RRV-RLI + TMZ as well as low beta-only and alpha-beta chain pathogen overlap is most representative of an anti-tumor immune response that is augmented by TMZ in the setting of RRV-RLI. While the strength of our conclusion that TCR profiling suggested a tumor-specific rather than viral-specific immune response was enhanced by following the reviewer's astute suggestion to add alpha-beta analysis, we also appreciate the reviewer's need to temper some of these conclusions and have therefore presented the new alpha-beta analysis in the results section of the revised manuscript (Page 10, Lines 411-412), while also moving our rationale and statement around the tumor-specific response to the discussion as suggested by the reviewer (Page 12, Lines 514-519).

Comment #9 - Minor Concerns:
In general, where scRNA-seq data is presented, the font size of text and axis labels appears too small, making it difficult to read. Below are specific instances where this issue needs attention:

- Figure 1A-C, 2B, 6D-G, 6H, 7: The font size of the axis labels, annotation and text is too small, making it difficult to read.
- Figure 4: The axis labels and text throughout the figure—except for Figure 4F—are not legible and should be adjusted.
- Figure 2B: The axes of the flow cytometry data are not labeled. It would be beneficial to include axis titles instead of relying solely on color coding.
- Line 185: 'Figure 3B' should be corrected to 'Figure 4B.'

These revisions will enhance readability and clarity across the figures.

Response #9: We thank the reviewer for their comment. We have made all the suggested changes to improve the readability and clarity of the figures. We appreciate the reviewer's insight.

In summary, we believe these revisions significantly solidify the mechanistic foundation and translational applicability of RRV-RLI therapy. As we plan to take this novel treatment to patients, we believe these additional studies provide a more rigorous assessment of the therapy and its potential for clinical translation. We thank the reviewers again for their constructive comments.

Sincerely,

Manish K. Aghi, MD PhD
Professor and Vice Chair of Neurosurgery, UCSF

Neuro-Oncology Program Leader, UCSF Cancer Center
Faculty, UCSF Graduate Division in Biomedical Sciences (BMS)

References for Response to Reviewers:

1. Saikh KU, Khan AS, Kissner T, Ulrich RG. IL-15-induced conversion of monocytes to mature dendritic cells. *Clin Exp Immunol*. 2001;126(3):447. doi:10.1046/J.1365-2249.2001.01672.X
2. Zannikou M, Duffy JT, Levine RN, et al. IL15 modification enables CAR T cells to act as a dual targeting agent against tumor cells and myeloid-derived suppressor cells in GBM. *J Immunother Cancer*. 2023;11(2):6239. doi:10.1136/JITC-2022-006239
3. Simon S, Labarriere N. PD-1 expression on tumor-specific T cells: Friend or foe for immunotherapy? *Oncoimmunology*. 2017;7(1):e1364828. doi:10.1080/2162402X.2017.1364828
4. Hiraoka K, Inagaki A, Kato Y, et al. Retroviral replicating vector-mediated gene therapy achieves long-term control of tumor recurrence and leads to durable anticancer immunity. *Neuro Oncol*. 2017;19(7):918-929. doi:10.1093/neuonc/nox038
5. Gubin MM, Zhang X, Schuster H, et al. Checkpoint Blockade Cancer Immunotherapy Targets Tumour-Specific Mutant Antigens. *Nature*. 2014;515(7528):577. doi:10.1038/NATURE13988
6. Mathios D, Park CK, Marcus WD, et al. Therapeutic administration of IL-15 superagonist complex ALT-803 leads to long-term survival and durable antitumor immune response in a murine glioblastoma model. *Int J Cancer*. 2016;138(1):187-194. doi:10.1002/ijc.29686
7. Genoud V, Marinari E, Nikolaev SI, et al. Responsiveness to anti-PD-1 and anti-CTLA-4 immune checkpoint blockade in SB28 and GL261 mouse glioma models. *Oncoimmunology*. 2018;7(12):e1501137. doi:10.1080/2162402X.2018.1501137
8. Chu TH, Vo MC, Lakshmi TJ, et al. Novel IL-15 dendritic cells have a potent immunomodulatory effect in immunotherapy of multiple myeloma. *Transl Oncol*. 2022;20:101413. doi:10.1016/J.TRANON.2022.101413
9. Ohteki T, Suzue K, Maki C, Ota T, Koyasu S. Critical role of IL-15-IL-15R for antigen-presenting cell functions in the innate immune response. *Nat Immunol*. 2001;2(12):1138-1143. doi:10.1038/NI729
10. Huang Q, Liu Y, Du Y, Garmire LX. Evaluation of Cell Type Annotation R Packages on Single-cell RNA-seq Data. *Genomics Proteomics Bioinformatics*. 2021;19(2):267-281. doi:10.1016/J.GPB.2020.07.004
11. Desbois M, Desbois M, Desbois M, et al. IL-15 superagonist RLI has potent immunostimulatory properties on NK cells: Implications for antimetastatic treatment. *J Immunother Cancer*. 2020;8(1):632. doi:10.1136/jitc-2020-000632
12. Haddad AF, Young JS, Amara D, et al. Mouse models of glioblastoma for the evaluation of novel therapeutic strategies. *Neurooncol Adv*. 2021;3(1):1-16. doi:10.1093/NOAJNL/VDAB100
13. Khan O, Giles JR, McDonald S, et al. TOX transcriptionally and epigenetically programs CD8+ T cell exhaustion. *Nature*. 2019;571(7764):211. doi:10.1038/S41586-019-1325-X
14. Wang X, He Q, Shen H, et al. TOX promotes the exhaustion of antitumor CD8+ T cells by preventing PD1 degradation in hepatocellular carcinoma. *J Hepatol*. 2019;71(4):731-741. doi:10.1016/J.JHEP.2019.05.015
15. Szulzewsky F, Schwendinger N, Güneykaya D, et al. Loss of host-derived osteopontin creates a glioblastoma-promoting microenvironment. *Neuro Oncol*. 2017;20(3):355. doi:10.1093/NEUONC/NOX165
16. Inoue M, Shinohara ML. Role of osteopontin and integrin alpha-v in T cell-mediated anti-inflammatory responses in endotoxemia. *J Immunol*. 2015;194(12):5595. doi:10.4049/JIMMUNOL.1500623

17. Kim TG, Kim CH, Park JS, et al. Immunological factors relating to the antitumor effect of temozolomide chemoimmunotherapy in a murine glioma model. *Clin Vaccine Immunol*. 2010;17(1):143-153. doi:10.1128/CVI.00292-09
18. Sanchez-Perez LA, Choi BD, Archer GE, et al. Myeloablative temozolomide enhances $CD8^+$ T-cell responses to vaccine and is required for efficacy against brain tumors in mice. *PLoS One*. 2013;8(3). doi:10.1371/JOURNAL.PONE.0059082
19. Sampson JH, Aldape KD, Archer GE, et al. Greater chemotherapy-induced lymphopenia enhances tumor-specific immune responses that eliminate EGFRvIII-expressing tumor cells in patients with glioblastoma. *Neuro Oncol*. 2011;13(3):324-333. doi:10.1093/NEUONC/NOQ157
20. Brandt K, Bulfone-Paus S, Foster DC, Rückert R. Interleukin-21 inhibits dendritic cell activation and maturation. *Blood*. 2003;102(12):4090-4098. doi:10.1182/BLOOD-2003-03-0669
21. Anguille S, Smits ELJM, Cools N, Goossens H, Berneman ZN, Van Tendeloo VFI. Short-term cultured, interleukin-15 differentiated dendritic cells have potent immunostimulatory properties. *J Transl Med*. 2009;7. doi:10.1186/1479-5876-7-109
22. Chu TH, Vo MC, Lakshmi TJ, et al. Novel IL-15 dendritic cells have a potent immunomodulatory effect in immunotherapy of multiple myeloma. *Transl Oncol*. 2022;20:101413. doi:10.1016/J.TRANON.2022.101413
23. Collins SA, Shah AH, Ostertag D, Kasahara N, Jolly DJ. Clinical development of retroviral replicating vector Toca 511 for gene therapy of cancer. *Expert Opin Biol Ther*. 2021;21(9):1199-1214. doi:10.1080/14712598.2021.1902982
24. Hoadley KA, Yau C, Hinoue T, et al. Cell-of-Origin Patterns Dominate the Molecular Classification of 10,000 Tumors from 33 Types of Cancer. *Cell*. 2018;173(2):291-304.e6. doi:10.1016/J.CELL.2018.03.022
25. Darmanis S, Sloan SA, Croote D, et al. Single-Cell RNA-Seq Analysis of Infiltrating Neoplastic Cells at the Migrating Front of Human Glioblastoma. *Cell Rep*. 2017;21(5):1399-1410. doi:10.1016/J.CELREP.2017.10.030

April 1, 2026

Dear *Nature Communications* Editor and Reviewers,

We are pleased to have received an acceptance in principle of our manuscript, "**Non-lytic viral immunotherapy induces long-term glioblastoma survival and tumor-specific immunity without eliciting an antiviral response"** for publication in *Nature Communications*.

We have uploaded the manuscript revised to address your editorial requests from the Author Checklist and any remaining comments from reviewers. Below, we address the remaining reviewer comments in a point-by-point fashion, with the editorial requests addressed in the checklist which we have also uploaded.

Reviewer #1 (Remarks to the Author)

The authors have answered all my questions.

Reviewer #2 (Remarks to the Author)

The revised manuscript has largely addressed the prior concerns. However, a few minor issues still need attention:

Comment #1: The line numbers cited in the response/rebuttal do not match those in the revised manuscript.

Response #1: We thank the reviewer for their comment. We have made sure that the line numbers in the previous rebuttal match those in the manuscript and have attached a revised version of that previous rebuttal with corrected line numbers at the end of this response document.

Comment #2: Some figures appear to be cited incorrectly in the response/rebuttal (e.g., The cross-presentation assay was shown in supplementary Fig. 6A,B, not in Supplementary Fig. 5H,I).

Response #2: We thank the reviewer for their comment. We have made sure that the figures cited in the rebuttal match those in the manuscript and have attached a revised version of that previous rebuttal with corrected line numbers at the end of this response document.

Comment #3: The references in the response/rebuttal appears out of order. For example, ref 10 (PMID: 25972484) is cited for "this label represents antigen-presenting cells as defined by singleR unsupervised cell labeling and CIITA, CD74, and CD86 expression"; ref 11 (PMID: 19889936) is cited for "…validated in the literature as producing receptor-linked-IL-15 that does not degrade into soluble IL-15"; ref 4 (PMID: 11702064) is cited for "prior work" related to "RRV infection alone does not significantly impact tumor growth…"
In addition, while 25 references are cited, only 21 are listed. Please ensure all cited references are included and correctly ordered.

Overall, the reviewer would appreciate a thorough cross-check of both the revised manuscript and the response/rebuttal to ensure internal consistency throughout.

Response #3: We thank the reviewer for their comment. We have made sure that the references in the response letter are updated and have attached a revised version of that previous rebuttal with corrected line numbers at the end of this response document.

Comment #4: Fig. 1 G,H legend: the prior comment was not requesting a definition of APC; rather, it asked what cell type represents APC (e.g., macrophage or DC). Please clarify this explicitly in the legend/text.

Response #4: We thank the reviewer for their comment. We defined the APC population using singleR unsupervised cell labeling and CIITA, CD74, and CD86 expression. As such, it represents a mixed population of myeloid cells, including macrophage and dendritic cell populations. We have updated the **Figure 1** legend to reflect this.

Mediating comments to our responses to Reviewer #3:

Comment #1: I have reviewed the response to Reviewer #3 and believe the authors have addressed the requests adequately. The remaining issues are minor and primarily relate to inconsistencies between the line numbers cited in the response and those in the revised manuscript, which made cross-referencing difficult. Although all points could be verified through a full review of the manuscript, this process was time-consuming.

Response #1: We thank the reviewer for their comment. We have made sure that the line numbers in the rebuttal match those in the manuscript and have attached a revised version of that previous rebuttal with corrected line numbers at the end of this response document.

We are thrilled to have received an acceptance in principle for this manuscript and thank you and the reviewers for guiding our work through the revision process.

Sincerely,

Manish K. Aghi, MD PhD
Professor and Vice Chair of Neurosurgery, UCSF
Neuro-Oncology Program Leader, UCSF Cancer Center
Faculty, UCSF Graduate Division in Biomedical Sciences (BMS)

Attachment - revised version of previous point-by-point rebuttal with corrected line numbers

February, 27th 2026 [updated to correct line numbers and figure numbering with new Reviewer 2 comments]

Dear *Nature Communications* Editor and Reviewers,

We are pleased to submit the revised version of our manuscript, "**Non-lytic viral immunotherapy induces long-term glioblastoma survival and tumor-specific immunity without eliciting an antiviral response"** for consideration for publication in *Nature Communications*.

We appreciate the reviewers' insightful comments on the manuscript. In response, we performed multiple novel experiments that expand the mechanistic findings, scope, and translational impact of our work. During the revision process, we completed eight new *in vivo* studies and three new *in vitro* studies, resulting in numerous new main and supplementary figure panels, the highlights of which include:

- To better understand the mechanisms around RRV-RLI treatment efficacy and synergy with temozolomide, we performed new T cell co-culture assays and dendritic cell cross-presentation assays.
- We completed CD4 T-cell and NK cell depletion experiments and expanded our previous CD8 depletion to a larger sample size to gain more clarity into the role of these cells in RRV-RLI efficacy.
- A common reviewer concern involved distinguishing the effects of the RRV backbone from the RLI transgene, and our choice to use intratumoral PBS treatment rather than RRV empty vector as a control arm in depletion experiments. After a review of the data with the editor, the editor told us that, based on the lack of survival benefit with RRV alone demonstrated in the original manuscript, she supported our choice not to include RRV empty vector as a control in depletion experiments but asked that we comprehensively compare intratumoral immune cells after intratumoral RRV-RLI, RRV, and PBS treatments via a large *in vivo* study. These data have been added to the revised manuscript and demonstrate no immune response to RRV alone, which, in combination with the lack of survival benefit from RRV treatment, implicates the RLI payload in the observed anti-tumor immune response occurring with RRV-RLI treatment.
- To strengthen the translational impact of our work, we added *in vivo* studies demonstrating the efficacy of RRV-RLI at a later time point and in a reduced dose.
- We validated RRV-RLI gene delivery and RLI protein expression ten days after administration, investigated viral biodistribution in the contralateral brain, and showed viral spread in multiple human patient-derived cell lines.

Below, we respond to the reviewer comments in a point-by-point fashion:

Reviewer #1's Comments:

Haddad et al. provides benefits of using replicating non-lytic retrovirus armed with IL-15 receptor-ligand fusion protein complex (RLI) against immunologically cold GBM tumors. The authors successfully show the persistence of the virus until the endpoint with effective enhancement of the activated CD8 T cells and NK cells in the tumor microenvironment (TME). The RRV-RLI significantly improved the survival of two mouse GBM models in comparison to the empty RRV control and PBS control. The authors were also able to observe induction of immunological response within TME using RRV-RLI as shown with Nanostring-based array. The authors also emphasize on the importance of CD8 T cells for the survival benefit observed with RRV-RLI in SB28 tumors and afterwards shows advantages of using TMZ in combination with RRV-RLI to

further enhance CD8 TCR repertoire & clonotypes against SB28 tumor using Single Cell RNAseq & TCR sequencing. While the findings of this paper are potentially significant to the field, the following concerns should be addressed prior to publication of the manuscript. Certain concerns: Issues that must be addressed to ensure the validity of conclusions made by the manuscript. (includes both major & minor concerns)

Comment #1: In Figure 1A, please specify which patient dataset has been used for this study.

Response #1: We thank the reviewer for their comment. For **Figures 1A-C**, we used publicly available data from the TCGA Pan Cancer Atlas. In the revised manuscript, we have mentioned that this data derived from the TCGA Pan Cancer Atlas in the revised results section (Page 4, Lines 88-89), provided additional details on the bulk RNA sequencing data in the Methods section in the subsection titled "Human bulk RNA-sequencing analysis," and added a figure reference to the end of this section to more clearly tie these methods to **Figures 1A-C** (Page 17, Line 781).

Comment #2: In Figure 1D-F, please specify if there is any evidence for the mechanism behind role of IL-15 in reducing M0 Macrophages in GBM context?

Response #2: We thank the reviewer for their comment. Figures 1D-F present immune-cell frequencies deconvolved from bulk RNA-seq of IL-15 high versus IL-15 low GBM samples, demonstrating a rise in $CD8^+$ T cells and monocytes and a relative reduction in non-polarized M0 macrophages. Two non-exclusive mechanisms likely contribute to the reduction in M0 macrophages: (1) Direct engagement of IL-15 signaling pathways on tumor associated macrophages which has been shown to induce an inflammatory (M1-like) state and thus reduce the M0 compartment;[1] and (2) Preclinical studies, including ours, in GBM have also shown an overall relative (not absolute) decrease in the entire myeloid compartment in response to IL-15 in the GBM TME,[2] which we hypothesize to be due to increased IL-15 mediated tumor infiltration of $CD8^+$ T-cells altering cellular proportions within the tumor and lowering the *relative* abundance of cell types other than $CD8^+$ T-cells, such as M0 Macrophages. We have added a sentence to our discussion highlighting these points (Page 11, Lines 437-440).

Comment #3: In line 98, does the single cell dataset also highlight increase in the expression pattern of immune checkpoints on tumor-infiltrating lymphoid and myeloid populations?

Response #3: We assessed the public GBM single-cell dataset we used to compare IL-15 high and low tumors for expression of classic checkpoint molecules (PDCD1/PD-1, CTLA4, LAG3, TIGIT and HAVCR2/TIM-3) in the tumor-infiltrating T cell and myeloid compartments. All molecules except HAVCR2 were detected in ≤5 % of lymphoid cells and were virtually absent from myeloid clusters, precluding a robust differential-expression analysis. There was no difference in the expression of HAVCR2, the only checkpoint molecules expressed by tumor-infiltrating T-cells and myeloid cells at sufficient levels to analyze, between the groups. This is a recognized limitation of single-cell RNA-seq, particularly for immune transcripts in brain tumors with minimal lymphocyte content. As a result, we cannot draw significant conclusions around differential checkpoint expression in these IL-15 high and low GBM tumors. To clarify this limitation, we have added a sentence to the results discussion (Page 4, Line 101-104). However, to further investigate the relationship between IL-15 signaling and immune checkpoint expression, we have performed additional co-culture experiments, which we discuss in **Response #5** below.

Comment #4: Typo! Change "an" to "a" in line 104.

Response #4: Thank you, we have corrected the an to a (Page 4, Line 107).

Comment #5: In Figure 2G-H, did the authors observe a direct impact of RRV-RLI on the expression of immune checkpoint markers on T cells through the co-culture assays? Scale of exhaustion of T cells!!

Response #5: We thank the reviewer for this comment. We have completed additional experiments where we repeated our T cell co-culture study and simultaneously assessed both T cell exhaustion/checkpoint marker expression and activation in response to RLI signaling. In this experiment, we found a small increase in PD-1 expression, likely consistent with activation rather than exhaustion, given the concordant increase in CD69 expression and lack of other concurrently expressed exhaustion markers (no change in expression of immune checkpoint markers TIM3, LAG3, and CTLA4, and no increase in double positive PD1/TIM3 and PD1/LAG3 populations was noted in response to RLI signaling), since PD-1 expression in the absence of expression of other exhaustion markers in the setting of CD69 co-expression is a sign of T-cell activation rather than exhaustion.[3] These findings suggest that there is not a shift towards checkpoint-high or exhausted T cell states in the presence of RLI. We have added this new data to the results section (**Supplementary Fig. 1I**, Page 5, Lines 156-161).

Comment #6: In Figure 3, authors should also show the secretion of RLI with tumor after 10 days post virus injection.

Response #6: Thank you for this comment. We agree and have performed additional *in vivo* experiments to address this concern. We now provide quantitative evidence of retroviral genome DNA (qPCR) and RLI protein (ELISA) intratumorally at 10 days post virus injection. These findings support the efficient delivery of RLI via viral tumor transduction. We have added these data to the results section of the manuscript (**Supplementary Fig. 2A-B**, Page 6, Lines 180-183).

Comment #7: In chapter "RRV-RLI induces significant anti-tumor modulation of the GBM immune microenvironment", authors should elaborate on the expression of checkpoint markers, state of dendritic cells, macrophages & neutrophiles.

Response #7: We thank the reviewer for their suggestion. While **Figure 4** is representative of bulk transcriptomic data generated via NanoString multiplex technology and limits the identification of cell specific states, we utilized our single cell RNA seq data set (presented in **Figure 7**) to address these points and examine the cell types that the reviewer mentioned. In our scRNA sequencing data, we find several changes in the immune cell types that the reviewer has asked about in response to RLI-RRV therapy:

- With regards to dendritic cells (including mCD1 and mCD2 populations), we see a reduction in markers of immature dendritic cells (Ifitm1 Log2FC -2.14, p_val_adj =3.29E-23, Klrb1b Log2FC -2.21, p_val_adj=4.28E-16, Adgrg5 Log2FC -2.59, p_val_adj=1.54E-13, Il1b Log2FC -1.35, p_val_adj=1.99E-19) and an increase in markers of dendritic cell activation (Il4i1 Log2FC 1.99, p_val_adj = 4.12E-12), antigen processing/presentation (Psme2 Log2FC 0.83, p_val_adj=1.25E-9, B2m Log2FC 0.53, p_val_adj=7.06E-9, Anxa2 Log2FC 0.97, p_val_adj=1.71E-8), and migration (Itgb3 Log2FC 3.14, p_val_adj =7.53E-5). These changes suggest an overall shift in dendritic cell populations from immature to mature and activated following RRV-RLI treatment *in vivo*. We have amended the results to reflect these results (**Supplementary Figure 5H** Page 9, Lines 358-361).
- Macrophage populations also experience a shift from a tolerogenic state to a reactive, phagocytic, and activated state in response to RRV-RLI treatment. In the presence of RRV-RLI, we observe a reduction in genes associated with immaturity (Il1r2 (LOG2FC -4.11, p_val_adj=6.37E-77, Pilra (LOG2FC -3.43, p_val_adj=5.27E-99) and tolerogenicity

(Clec4n (LOG2FC -3.38, p_val_adj=3.29E-133). Concurrently, we observe an increase in genes related to reactivity (Lgals3 LOG2FC 2.09, p_val_adj=3.81E-141), lysosomal activity (Ctsb LOG2FC 1.15, p_val_adj=1.32E-93), and complement/cytokine signaling (C3 LOG2FC 1.70, p_val_adj=1.89E-40, Ccl2 Log2FC 2.16, p_val_adj=9.78E-52, Ccl7 Log2FC 2.95, p_val_adj=1.11E-72) We have amended the results to reflect these results (**Supplementary Figure 5G,** Page 9, Lines 358-361).

- As mentioned above, we don't see checkpoint markers as significantly differentially expressed genes between treatment groups in myeloid populations. Comparisons in checkpoint marker expression between the different treatment groups in lymphocytes is limited given the very low number of infiltrating T cells in PBS treatment mice. When assessing our scRNA sequencing data, we observe that less than 1% of infiltrating lymphocytes are labeled as "exhausted $CD8^+$ T cells" in all treatment groups (**Figure 7E**). Furthermore, as discussed in **Response 5** we do not see a significant change in checkpoint marker expression in T cells co-cultured with RRV-RLI infected tumor cells in vitro (**Supplementary Figure 1I**).
- We discuss neutrophil populations specifically in **Response #8** below.

Comment #8: In chapter "RRV-RLI induces significant anti-tumor modulation of the GBM immune microenvironment", the authors also observe increase in the expression of markers relative to neutrophils. Can the authors explain whether the neutrophil phenotype is tumor-supportive or pro-inflammatory?

Response #8: We thank the reviewer for their insightful comment. Figure 4 is representative of bulk transcriptomic data (Nanostring) that makes it difficult to infer the state of specific cell populations. As such, we have turned to our single cell RNA sequencing data set (**Figure 7**) to further assess infiltrating neutrophil populations and gain additional insight into the infiltrating neutrophil phenotypes. Our scRNA data reveals that infiltrating neutrophils are a relatively rare population in the tumor infiltrating immune milieu (e.g., only 23 neutrophils in the PBS + Vehicle group), limiting the conclusions from our insights. However, when we compare infiltrating neutrophils between RRV-RLI + TMZ and PBS + TMZ, we observe an overall decrease in markers of a tumor-supportive myeloid-derived suppressor cell (MDSC) phenotype in mice treated with RRV-RLI. This is supported by reduced expression of Arg1 (LOG2LOG2FC -3.57, p_adj = 4.29E-07), Chil3 (LOG2FC -3.84, p_adj = 5.58E-24), Mmp8 (LOG2FC -2.8, p_adj = 2.44E-10), Lyz2 (LOG2FC -2.01, p_adj = 6.05E-9), Apoe (LOG2FC -1.87, p_adj=6.81E-6) in neutrophils infiltrating RRV-RLI treated mice relative to control. This is consistent with reduced tumor supportive/immune suppressive programs in RRV-RLI treatment. We have added these findings to the results section of our manuscript (Page 9, Lines 358-361) and to **Supplementary Figure 5F.**

Comment #9: In chapter "RRV-RLI induces significant anti-tumor modulation of the GBM immune microenvironment", as the authors observe a significant increase in exhausted CD8 T cells. Does it go by the hypothesis of enhanced CD8 T cells potency against tumor with RRV-RLI, or is the survival benefit of RRV-RLI is happening due to a completely different immune cell subset?

Response #9: We thank the reviewer for this comment. While **Figure 4F** demonstrates an increase in the immune infiltration score associated with exhausted $CD8^+$ T cells in the RRV-RLI treatment group when compared to control, we believe that this is reflective of the absolute increase in the number of $CD8^+$ T cells rather than an increase in the relative ratio of exhausted and non-exhausted $CD8^+$ T cell populations. Indeed, when we assess the ratio of exhausted to non-exhausted $CD8^+$ T cells, the ratio in the RRV-RLI treatment group is more favorable than that observed in the control group (1.2 vs. 0.84, p=0.04, Student's t-test). To make this clearer, we have added a sentence to the results section highlighting the favorable exhausted to non-

exhausted ratio in RRV-RLI treatment (Page 6, Lines 217-220). In addition, we have performed additional T cell co-culture experiments demonstrating no significant increase in exhausted T cell populations after exposure to RRV-RLI infected tumor cells, as discussed in **Response 5.** Our scRNA dataset also supports that there is not proportionally higher T cell exhaustion in RRV-RLI treated mice, as discussed in **Response 7.**

Regarding the reviewer's question of whether the survival benefit of RRV-RLI could be happening due to a completely different immune cell subset, we do believe that T cells are the primary effector cell type of RRV-RLI treatment response based on the depletion studies in **Figure 5**, where dual CD4/CD8 depletion abrogates the treatment benefit of RRV-RLI treatment, but there is no significant effect on RRV-RLI benefit with NK cell depletion.

Comment #10: In Line 185, typo for the figure number; 'Fig 3B to "Fig 4B".

Response #10: Thank you, we have corrected the Figure number to 4B (Page 6, Line 206).

Comment #11: In Figure 2F, the comparison has been made between PBS and RRV-RLI to analyze changes in the immune cell populations. The authors should show a similar analysis done between RRV & RRV-RLI to support the effect of RLI in the experiment.

Response #11: We thank the reviewer for this comment. We have completed the requested experiment to assess the impact of RRV on the immune microenvironment by performing additional *in vivo* experiments where we directly compare immune infiltration in RRV vs. RRV-RLI-treated tumors at a matched timepoint via flow cytometry. This flow cytometric analysis demonstrates significantly higher infiltration of $CD3^+$ T cells, $CD8^+$ T cells, NKT cells, and NK cells in RRV-RLI treatment groups relative to both PBS and RRV treatment groups, with no significant difference between PBS and RRV treatments (**Figure 6I-M**). Similarly, RRV-RLI results in a proportional decrease in infiltrating $CD11b^+$ cells relative to RRV and PBS treatments with no difference between RRV and PBS groups (**Figure 6N**). We have amended the results section (Page 8, Line 298-311) and **Figure 6** to reflect these findings.

In summary, transcriptomic (**Supplementary Figure 2E-H**), co-culture experiments (**Figure 2G-I, Supplementary Figure 1D-H**), flow cytometric (**Figure 6I-N**), and survival data (**Figure 3B-E**) demonstrate no significant difference between PBS and RRV treatment groups, whereas RRV-RLI demonstrated striking differences from both. These data support that the immune modulation and therapeutic benefit of RRV-RLI is due to the RLI payload rather than the RRV vector backbone. We have added additional reasoning around this topic to the discussion section (Page 12, Line 476-480).

Comment #12: In Figure 2D legend, the author should correctly specify that heatmap belongs to RRV Vs RRV-RLI.

Response #12: Thank you very much for your comment. We have amended **Supplementary Figure 2** legend to reflect that **Panel D** (now **Panel G** after revisions) compares RRV-RLI to RRV.

Comment #13: In Figure 5, why was the survival comparison was done on PBS Vs RRV-RLI in-spite of RRV Vs RRV-RLI? A survival comparison between RRV Vs RRV-RLI is important to specifically determine the role of CD4/CD8 T cells via RRV-RLI therapy against SB28 tumor in vivo.

Response #13: We thank the reviewer for their insightful comment. We discussed the use of PBS as a control group with the editor, who informed us that we did not need to use RRV-treated tumors as a survival control group in the depletion study, but that we should instead perform further immune profiling of RRV-treated tumors to demonstrate similarity to PBS-treated tumors (as we did in new **Figures 6I-N**). We concur with the editor's rationale for this decision based on our co-culture experiments (**Figure 2G-I, Supplementary Figure 1D-H**), *in vivo* transcriptomic (**Supplementary Figure 2E-H**), *in vivo* flow cytometric (**Figure 6I-N**), and survival data (**Figure 3B-E**) revealing no significant difference between PBS and RRV treatment groups, whereas RRV-RLI demonstrated differences from both in each of these critical parameters. Indeed, in addition to our own data, prior work [4] has demonstrated that RRV infection alone does not significantly impact tumor growth, survival, or immune infiltration compared to PBS controls. Given the lack of survival difference between RRV and PBS alone, we wouldn't expect to see a difference in CD4/CD8 depletion survival outcome between RRV and PBS-treated mice. Our mechanistic goal was to assess if the survival benefit of the clinically intended therapy (RRV-RLI) depends on various effector cell populations (CD4/CD8/NK) and we believe that using PBS as the baseline isolates the contribution of these populations in the most translationally relevant fashion. For additional clarity, we have clarified this rationale in the discussion section of the manuscript (Page 12, Line 476-480).

Comment #14: In Figure 5, using CD8 depleting antibody could also reduce CD8+ dendritic cell population which derives the activation of naive CD8 T cells by presenting tumor-specific antigen in the tDLNs. Can the author show that the use of CD8 depleting antibody does not affect the dendritic cell subsets in tDLNs or TME?

Response #14: We thank the reviewer for this helpful comment. In our study, both flow cytometry and single-cell RNA sequencing revealed that $CD8^+$ dendritic cells were so sparse in our murine GBM tumor microenvironment (<0.75% of infiltrating immune cells, **Supplementary Figure 4E**) that the impact of CD8 depletion on these cells could not be reliably assessed. We have added a panel to **Supplementary Figure 4E** demonstrating the lack of this population on flow cytometry and have clarified this in the results section (Page 8, Lines 307-311). This finding is consistent with prior studies assessing the role of $CD8^+$ T cells in immunotherapy models using CD8-depleting antibodies, which have not required separate evaluation of CD8+ dendritic cell populations to ensure that their findings are due to effects of CD8 depletion on T-cells.[5,6] Furthermore, we found that CD8 depletion alone did not impact RRV-RLI's therapeutic benefit, with only the combination of CD8 and CD4 depletion impacting RRV-RLI's therapeutic benefit, suggesting that RRV-RLI requires the two T-cell subsets together for its effect rather than any $CD8^+$ cells alone. In summary, we believe our data makes it unlikely that off-target depletion of CD8 dendritic cells accounts for the abrogation of RRV-RLI therapeutic efficacy observed in the CD4/CD8 depletion experiment. We have amended the discussion to highlight the need for CD4 and CD8 T cells for RRV-RLI efficacy (Page 12, Lines 487-496). and the methods to reflect the decision not to evaluate dendritic cell populations (Page 16, Lines 680-682).

Comment #15: In Figure 5G, it seems that CD8 depletion still show benefit of survival in with RRV-RLI, directing towards the importance of potential CD4 T cell mediated RRV-RLI efficacy in SB28 mGBM model. The authors should also show survival data by depleting CD4 cells alone in RRV Vs RRV-RLI treatment groups.

Response #15: We thank the reviewer for their comment. In accordance with the reviewer's suggestion, we did perform a $CD4^+$ T cell depletion experiment that continues to show a significant survival benefit in mice treated with RRV-RLI relative to PBS control (18 vs. 44 days, p=0.0001, Log-Rank, **Figure 5H**). We have amended the results (Page 7, Lines 250-252) and **Figure 5** to

reflect these findings. We used PBS control as the control arm given the lack of survival benefit or immunologic change seen with RRV alone treatment, as discussed in **Response 13**. Furthermore, to increase the reliability with which our CD8 depletion results (which revealed a lack of therapeutic benefit for RRV-RLI with CD8 depletion that was barely statistically meaningful) could be interpreted alongside our other depletion results, we repeated the CD8 depletion experiment concurrently with our other depletion studies with a larger number of mice. Upon repeat of the CD8 depletion, we do now observe a significant survival difference between RRV-RLI and PBS groups in the setting of CD8 depletion (43.0 days vs. 18.0 days, p=0.03, Log-Rank Mantel-Cox, **Figure 5F**), suggesting a compensatory response from CD4 T cells and NK T cells in this setting. These findings suggest that $CD8^+$ and $CD4^+$ T cells coordinate to serve as the primary co-drivers of RRV-RLI therapeutic efficacy, with both together needed for this survival benefit in a manner that the therapeutic benefit can continue as long as one of these T-cell subtypes is present, but is lost once both are gone, a versatility that has implications for the translatability of this approach where patients could derive a benefit unless they had a dramatic reduction in levels of both T-cell subtypes. We have amended the results (Page 7, Lines 246-252) and **Figure 5** to reflect these findings.

Comment #16: In Figure Supplementary 3E-F, has the anti-PD1 treatment compared between RRV Vs RRV-RLI?

Response #16: We thank the reviewer for this comment. In **Supplementary Figure 3E–F** (now **Supplementary Figure 3F-G**), anti-PD1 treatment was compared to isotype control in the context of PBS-treated tumors, not RRV-treated tumors. This was done because the goal was to establish whether anti-PD1 alone could improve survival or tumor control in the highly immunosuppressive SB28 model. Our findings were consistent with previous reports demonstrating that SB28 is not responsive to checkpoint blockade.[7] In **Supplementary Figure 3H-I**, we then tested anti-PD1 in combination with RRV-RLI, showing that even with enhanced immune infiltration, PD-1 blockade did not significantly improve outcomes relative to RRV-RLI alone. We did not include an RRV + anti-PD1 arm, as RRV monotherapy has minimal survival or immunologic effects in this model (see **Responses 11 and 13**), a decision supported by the editor in our discussion with her. The PD-1 experiment also clarified that PD-1 signaling alone was not a primary driver of tumor progression after successful initial treatment with RRV-RLI.

Comment #17: In the chapter "Antitumoral efficacy of RRV-RLI against murine GBM is potentiated by systemic chemotherapy", how does the author justify the use of a replicating non-lytic RRV which could also lead the release of tumor-specific antigens, to that of the use of TMZ in combination of RRV-RLI to enhance the same?

Response #17: We thank the reviewer for this comment. Replicating retroviral vectors do not lyse cells as part of their lifecycle, making them unique relative to most vectors used in cancer treatment which have been traditional oncolytic vectors such as Herpes or Adenovirus. As such, replicating retroviral infection alone does not lead to significant tumor cell death and the therapeutic benefit is primarily generated from the payload that is delivered. This is reflected in the lack of survival benefit or immunologic change seen in RRV alone treatment when compared to RRV-RLI. We posit that combination of RRV-RLI with TMZ is synergistic because TMZ leads to some amount of tumor cell death, which provides additional antigens for the immune system to generate an anti-tumor immune response. We believe this is supported by our single cell RNA and T-cell receptor sequencing data which shows that mice who receive RRV-RLI in combination with TMZ have increased T cell receptor clones relative to RRV-RLI mice alone. Our data also demonstrates that RLI expression in the TME increases antigen presentation pathways, further supporting this proposed mechanism.

To further clarify the role of TMZ releasing tumor antigens in its synergy with RRV-RLI, in the revised manuscript, we have performed a functional cross-presentation assay. In this experiment, we utilize SB28 tumor cells expressing ovalbumin (OVA) and assess the ability of dendritic cells pulsed with their supernatant to activate OT-1 CD8 T cells (which recognize OVA SIINFEKL peptide presented by the MHC class I). We compare OT-1 activation between SB28 OVA cells treated with TMZ to those without TMZ treatment. In SB28 OVA cells treated with TMZ, we see an increase in downstream OT-1 T cell activation (as evidenced by $CD69^+$, 13.8% vs. 8.8%, $p<0.05$, Welch's t-test, **Supplementary Figure 6A**) and cell proliferation (as evidenced by $Ki67^+$, 11.7% vs. 7.2%, $p<0.05$, Welch's t-test, **Supplementary Figure 6B**). These data support a model where TMZ cell death increases antigen supply, which then synergizes with RRV-RLI enhancement of T cell, NK cell, and antigen presentation responses. We have amended the results section (Page 10, Lines 375-380), **Supplementary Figure 6A-B,** and the discussion (Page 13, Lines 527-531) to reflect these findings.

Comment #18: In Figure 6A-B, the authors have shown comparisons of combining TMZ with PBS or RRV-RLI but have not included RRV control with TMZ to justify the benefit of RLI!!

Response #18: We thank the reviewer for this comment. We discussed this with the editor who felt that we did not need to include a RRV+TMZ treatment arm because prior data in our model demonstrated that RRV alone (without a therapeutic payload) has minimal effects on tumor growth, survival, or immune modulation (as discussed in **Responses 11 and 13**). Specifically, RRV alone showed no significant benefit or difference compared to PBS in survival or immune infiltration. Given this lack of effect, and to minimize additional animal use, we focused on comparing PBS ± TMZ and RRV-RLI ± TMZ to directly evaluate the efficacy of TMZ treatment in combination with RRV-RLI. To further address the reviewer's astute comment, and at the editor's suggestion, we completed an additional *in vivo* study and flow cytometry of SB28 tumors from mice treated with RRV±TMZ. We added this data to **Figure 6I-N**. We demonstrate no difference in immune cell infiltration between mice treated with RRV ± TMZ and PBS ± TMZ. In addition, we show that there is a significant increase in $CD3^+$ T cells, $CD8^+$ T cells, NKT cells, and NK cells in mice treated with RRV-RLI ± TMZ when compared to RRV ± TMZ. We have added this new data to the results section (Page 8, Line 291-303) and further explained the rationale behind using PBS as the primary control group for these experiments and others in the discussion section (Page 12, Line 476-480).

Comment #19: In Supplementary Fig. 4E, we can observe a significant decline in cDC2 conventional DCs with TMZ+RRV-RLI. Does the Single cell RNAseq data reveal more about the effect of this combination on DCs subsets, their functionality & antigen-presentation potential?

Response #19: We thank the reviewer for this thoughtful comment. As part of these revisions, we repeated flow cytometric analysis of *in vivo* mouse SB28 tumors treated with RLI-RRV±TMZ, RRV±TMZ, and PBS±TMZ to avoid batch effects. When analyzing these new samples, we no longer see the small but significant difference noted in cDC populations between RRV-RLI + Vehicle and RRV-RLI + TMZ that you refer to. In fact, we do not see a significant difference in infiltration of $CD11b^+CD11c^+MHCII^+$ immune cells between any treatment group (**Supplementary Figure 4D**). We do see a small but significant increase in RRV-RLI treatment groups (no difference between Vehicle and Control) in infiltrating $CD11b^+CD11c^+MHCII^+$ $CD8^+$ immune cells (**Supplementary Figure 4E**), but these populations are so rare in the tumors (<0.75%) that it makes gleaning true biological insights difficult. To further investigate the effect of RRV-RLI + TMZ on dendritic cells, we have utilized our single-cell RNA sequencing data. When evaluating overall myeloid populations, we see an increase in antigen processing/presentation machinery that is

highest in RRV-RLI+TMZ followed by RRV-RLI+Vehicle, suggesting synergy between the increased antigen presence afforded by TMZ treatment and the RLI payload (**Figure 7F**). We see a similar trend in dendritic cell populations, specifically between RRV-RLI + TMZ and RRV-RLI + Vehicle. In the setting of RRV-RLI+TMZ treatment, tumor-infiltrating DCs express increased markers of antigen presentation machinery (Ly75 Log2FC 1.73, p_val_adj=1.06E-16, H2-Q4 Log2FC 0.97, p_val_adj=7.4E-05) as well as markers of activation (IL12b Log2FC 1.82, p_val_adj=9.05E-10). We have added these findings into the results section (Page 9, Line 361-363).

Comment #20: In line 319, the authors should figure they are referring the flow cytometry data of!!

Response #20: We thank the reviewer for their comment. We have amended the sentence to reflect that we are referencing the flow cytometry data in **Figure 6N** which demonstrates a reduction in tumor-infiltrating CD11b myeloid cells (Page 8, Line 304-307).

Comment #21: In Figure 7E, the dendritic cell population is seemingly reducing with RRV-RLI only treatment in comparison to PBS which is different from the Figure 4F. The authors should reason this difference.

Response #21: We thank the reviewer for this insightful comment. We assess tumor-infiltrating dendritic cells using three different modalities in the manuscript- bulk transcriptomic analysis (**Figure 4F**), flow cytometric analysis (**Supplemental Figure 4D-E**), and 5' single cell RNA sequencing (**Figure 7E**). The reviewer points out that in **Figure 4F** the dendritic cell transcriptomic tumor infiltrating score appears increased in the RRV-RLI treatment group, while it appears qualitatively slightly decreased in the single cell RNA sequencing in **Figure 7E**. In our flow cytometric analysis (**Supplemental Figure 4D**), we do not see a significant difference in overall $CD11b^{+}CD11c^{+}MHCII^{+}$ immune cell infiltration between RRV-RLI and PBS treatment groups. In addition, in both our single-cell RNA sequencing data and bulk transcriptomic data we see an increase in antigen presentation pathways and expression of MHC-related genes. As such, our conclusion is that intratumoral RLI expression does not lead to grossly increased dendritic cell infiltration but rather increases antigen presentation pathways on existing antigen-presenting cells in the tumor microenvironment. This aligns with the IL-15 literature, which demonstrates that IL-15 supports antigen presentation and increases MHC, CD40, and CD86 expression on dendritic cells.[8,9] In terms of integrating our results across the three platforms as the reviewer is asking for in this comment: as increased antigen presentation pathways identified by single-cell RNA sequencing (**Figure 7F** and **Supplementary Figure 5H)** may slightly inflate calculated immune infiltration scores, which is based on transcriptomic gene expression (**Figure 4F**), without leading to an increase in gross infiltration on flow cytometric analysis (**Supplementary Figure 4D**). We have added an additional point to our discussion, clarifying this difference and appreciate the reviewer's point (Page 11, Lines 470-472).

Reviewer #2's Comments:

In the manuscript by Haddad et al., the authors generated a non-lytic replicating retrovirus (RRV) expressing the receptor-linked IL-15 (RLI) and evaluated its anti-tumor efficacy in murine GBM models. The combination treatment efficacy of RRV-RL1 with TMZ is also evaluated. RRV-RL1 treatment prolongs mice survival which is even improved when combined with TMZ. The immune responses, specifically T-cell responses, have been analyzed via the antibody-based depletion strategy and single cell RNA/TCR analysis. Developing the new virus-based therapeutic strategy

to treat GBM is clinically relevant. However, the mechanistic action of RRV-RLI treatment as defined in the current study is largely descriptive.

Comment #1: Fig. 1G, H: The proportion of cell types in the Alluvial plot (Fig. 1H) does not clearly match those presented in the UMAP plots (Fig. 1G), and what is the cellular identity of APC?

Response #1: We thank the reviewer for their comment. Regarding differences in proportions of cell types between **Figure 1G and H**, the UMAP in **Figure 1G** is a pooled 2-D visualization which can be distorted by point overlap and local crowding. As such, **Figure 1G** might not perfectly match the proportions in **Figure 1H**, which instead reports per-sample cell counts normalized to the total number of cells and is more quantitative in nature. To further clarify this, we have added a note to the methods that proportions in the alluvial plot were computed from per-sample counts (Page 18, Lines 795-796).

Regarding the cellular identify of APC, this label represents antigen-presenting cells as defined by singleR unsupervised cell labeling and CIITA, CD74, and CD86 expression.[10] We have clarified the definition of APCs in the **Figure 1** legend and specify in the methods section that "subclassification of immune cells was achieved using labels suggested by SingleR and marker gene expression identified through literature review" (Page 18, Line 788-790).

Comment #2: Fig. 2, Supplementary Fig.1, Supplementary Fig. 2A and throughout the manuscript: It is not clear if the secreted RLI is a true receptor-linked–IL-15, or just soluble IL-15. Furthermore, it is not clear if the RRV-RLI treatment effects result from the secreted receptor-linked–IL-15, or just soluble IL-15.

Response #2: We thank the reviewer for raising this clarifying point. The RRV-RLI vector only encodes receptor-linked-IL-15 not soluble IL-15. This is human IL-15 that is fused to an IL-15 receptor alpha sushi domain using a flexible linker sequence that has been previously validated in the literature as producing receptor-linked-IL-15 that does not degrade into soluble IL-15.[11] Our vector therefore does not express IL-15 alone and therefore only leads to the secretion of receptor-linked-IL-15, which does not ultimately give rise to soluble IL-15. We have confirmed our vector sequence using Sanger sequencing. We have also demonstrated stability of our vector transgene at the expected size for the RLI coding sequence on PCR (**Supplemental Figure 1C**) after over 8 weeks of continuous vector replication and spread in vitro. The PCR primers we utilize span the entire RLI construct, including the IL-15 and IL-15 receptor linker region, confirming a fusion-specific amplicon. We demonstrated the functionality of our RLI construct in **Figure 2F-I**. To further confirm that our vector is specifically delivering and leading to the production of RLI rather than soluble IL-15, we have also confirmed sequence-specific integration into tumor cells *in vivo* 10 days after infection using a virus-specific qPCR that is only specific to our viral genome sequence (**Supplementary Figure 2A**, Page 6, Lines 179-183). We believe that altogether, our findings support that the treatment effects seen with RRV-RLI can be attributed to the RLI fusion protein, not soluble IL-15. We have added these findings to the results section for additional clarity, and appreciate the reviewer bringing up this point.

Of note and somewhat beyond the question of which form of IL-15 was delivered, coordinately performed ELISA also confirmed that the RLI produced after *in vivo* expression was detectable by an anti-IL-15 antibody capable of detecting the IL-15 moiety within RLI (**Supplementary Figure 2B**, Page 6, Lines 179-183) that correlated with RRV-RLI viral infection with no evidence of IL-15 or RLI protein in PBS-treated mice, underscoring how low levels of IL-15 in murine gliomas made them well suited for RRV-RLI treatment, similar to the findings from the TCGA data in **Figure 1** across multiple cancers.

Attachment - revised version of previous point-by-point rebuttal with corrected line numbers

Comment #3: Fig. 2F.G: It is not clear about what is the "untreated" group. Are these mice age-matched to the re-challenged mice?

Response #3: We thank the reviewer for their comment. Yes, the mice in the untreated group are tumor-bearing mice that are age-matched to the rechallenged mice and were not treated. We have added additional text in the results and methods to clarify this point (Page 6, Lines 177-178 and Page 15, Lines 656-657).

Comment #4: Fig. 4: The analysis based on the comparison of RRV-RLI group to the PBS group cannot clearly distinguish the immune changes as a direct result of anti-RRV or RLI or anti-tumor effect.

Response #4: We thank the reviewer for their insight. Prior work[4] and our own data demonstrate that RRV infection alone does not significantly impact tumor growth, survival, or immune infiltration compared to PBS controls, and both differ significantly from RRV-RLI. We do not observe any survival benefit associated with RRV treatment (**Figure 3B-E**). We addressed this comment when, transcriptomically, we showed RRV vs. RRV-RLI transcriptomic immune-infiltration comparison as a heat map in **Supplementary Figure 2H,** a heat map demonstrating differences in gene expression between RRV and RRV-RLI in **Supplementary Figure 2G,** a volcano plot demonstrating differential gene expression between RRV-RLI and RRV in **Supplementary Figure 2F,** and a figure demonstrating differences in gene pathways between RRV and RRV-RLI in **Supplementary Figure 2E**. **Supplementary Figure 2H** also includes mice at endpoint who progressed after initial RRV-RLI response, revealing that progression after initial response was associated with reversion of the immune microenvironment to an immunologically "cold" state with low T and NK cell infiltration, similar to results seen with RRV. In addition, co-culture experiments with T and NK cells show no increase in activation or proliferation with RRV or untreated tumor cells (**Figure 2G-I, Supplementary Figure 1D-H**), but show a large increase in activation and proliferation with RRV-RLI treated cells, implicating RLI, not RRV, in the biological changes seen after RRV-RLI treatment.

To further address the reviewer's excellent point, after discussion with the editor, and to further assess the impact of RRV on the immune microenvironment, we completed additional *in vivo* experiments where we directly compared immune infiltration in mice treated with RRV-RLI vs. RRV at a matched timepoint via flow cytometry. This flow cytometric analysis demonstrates significantly higher infiltration of $CD3^+$ T cells, $CD8^+$ T cells, NKT cells, and NK cells in RRV-RLI treatment groups relative to both PBS and RRV treatment groups, with no significant difference between PBS and RRV treatments (**Figure 6I-M**). Similarly, RRV-RLI results in a proportional decrease in infiltrating $CD11b^+$ cells relative to RRV and PBS treatments with no difference between RRV and PBS groups (**Figure 6N**). We have amended the results section (Page 8, Lines 293-311) and **Figure 6** to reflect these findings. In summary, transcriptomic (**Supplementary Figure 2E-H**), flow cytometric (**Figure 6I-N**), and survival data (**Figure 3B-E**) demonstrate no significant difference between PBS and RRV treatment groups, whereas RRV-RLI demonstrated striking differences from both. In addition, in our single cell TCR sequencing data we do not see TCRs that are targeted against the MLV envelope and only 2.3% of TCRs were within 18 distance units of a pathogen-specific TCR (**Figure 7H** and Page 10, lines 403-405). These data support that the immune modulation and therapeutic benefit of RRV-RLI is due to the RLI payload rather than the RRV vector backbone. We have added additional reasoning around this topic to the discussion section (Page 11-13, Lines 475-534).

.

Comment #5: Fig. 4F: Why exhausted CD8 score is increased in the RRV-RLI group vs PBS? Again, Supplementary Fig. 2E: why exhausted CD8 is higher in RRV-RLI TP vs RRV-RLI EP or RRV TP?

Response #5: Regarding the higher "exhausted" CD8 score in the RRV-RLI treated group as compared to the PBS treatment groups, we believe this due to multiple factors. First, there is extremely low T cell infiltration in PBS and RRV treated SB28 mice, as can be seen in our flow cytometric analysis (**Figure 6**, <5% infiltration for $CD3^+$, $CD4^+$, and $CD8^+$ T cell populations in PBS and RRV treated SB28 tumors), which is consistent with the literature for SB28[7,12] and part of the reason that SB28 is seen as a very immunologically "cold" model that recapitulates human GBM. As such, the increase in "exhausted" $CD8^+$ score on transcriptomic analysis is likely reflective of an absolute change in the number of $CD8^+$ T cells with RRV-RLI treatment rather than an increase in the relative ratio of exhausted and non-exhausted CD8 T cell populations. Indeed, when we assess the ratio of exhausted to non-exhausted $CD8^+$ T cells, the ratio in the RRV-RLI treatment is more favorable than that observed in the control group (1.2 vs. 0.84, p=0.04, Student's t-test). Second, a higher exhausted $CD8^+$ score in the RRV-RLI treatment group is, in many ways, expected with antigen-driven activation. In antigen-driven activation, activated $CD8^+$ T cells can upregulate markers such as PD1, which are markers of both activation and exhaustion.[3] The upregulation of these markers may contribute to a higher "exhausted" $CD8^+$ T cell score, but, in fact, is likely representative of activation. This is supported by the 5' single-cell RNA sequencing data (**Figure 7C**), which examines PBS and RRV-RLI-treated mice. In the scRNA sequencing data, we observe a small population of "exhausted" $CD8^+$ T cells, but much larger populations of $CD8^+$ effector cells, early activated T cells, and NKT cells.

We also performed additional *in vitro* experiments to directly evaluate the impact of RLI exposure on T cell exhaustion markers. In these experiments, we observe an increase in expression of activation marker CD69 with concordant expression of PD-1 likely more reflective of it as an activation marker rather than its role as an exhaustion marker because we observed no significant expression of any other exhaustion marker (TIM3, LAG3, CTLA4) and also found no concordant expression of these other exhaustion markers alongside PD-1.[3] We have amended the results to reflect these findings. (**Supplementary Fig. 1I**, Page 5, Lines 156-162).

We believe a similar rationale applies to **Supplementary Fig. 2E**, primarily that an increased "exhausted" CD8 T cell score is primarily indicative of increased overall T cell infiltration and antigen driven activation in RLI-RRV treated mice at timepoint, during high levels of immune activation, as compared to empty RRV at timepoint (TP) or RRV-RLI treated mice that have escaped treatment at endpoint (EP). We have added additional clarification around this topic to the discussion section (Page 11-12, Lines 466-470).

In summary, through leveraging transcriptomic ratios, our scRNA sequencing data, and additional *in vitro* experiments, we believe that the increased "exhaustion score" is in fact representative of increased infiltration and activation rather than true exhaustion

Comment #6: Fig. 5: The tumor volume and mouse survival in the RRV-RLI + isotype control group need to be compared to those in the RRV-RLI + anti-CD4/CD8 group and those in the RRV-RLI + anti-CD8 group. Additionally, the tumor volume and mouse survival in the PBS + isotype control group need to be compared to those in the PBS + anti-CD4/CD8 group and those in the PBS + anti-CD8 group.

Response #6: We thank the reviewer for their comment. We have added additional depletion experiments, including both CD4 and NK cell depletion alone, as well as a repeat of the CD8 depletion with a larger sample size. As the reviewer asked, we have compared all the RRV-RLI treatment groups across experiments. In this analysis, only the combined CD4 and CD8 depletion (where RRV-RLI efficacy was completely abrogated) significantly differed from the RRV-RLI

treatment groups (**Supplementary Figure 3E**, Page 7, Lines 251-253). When comparing the PBS treatment groups, we observed minor differences between isotype and combined CD4 and CD8 depletion as well as isotype and NK depletion. Median survival in the PBS treatment groups across depletion experiments ranged from 15 to 20 days. Given the small difference in survival between the PBS groups we do not consider them biologically significant but have added the data to the results section (Page 7, Lines 251-253) and **Supplementary Figure 3D**. In summary, our data reveal that only combined CD4 and CD8 depletion completely abrogates the RRV-RLI treatment response.

Comment #7: Fig. 4 and Supplementary Fig. 2 indicate that NK cell response is increased in the RRV-RL1 group. Expansion of NK cells is pronounced in RRV-RL1 +/-TMZ group (vs PBS +/-TMZ group) and NK cells are the most abundant in these 2 groups, as shown in Fig. 7C. The contribution of NK cells to tumor progression and mice survival upon RRV-RL1 treatment +/-TMZ needs to be experimentally evaluated.

Response #7: We thank the reviewer for their comment and agree with their rationale. In response to this point, we did complete an *in vivo* NK cell depletion experiment to examine the impact of NK cells on RRV-RLI therapeutic efficacy. These data demonstrated that NK depletion did not abolish the survival benefit seen with RRV-RLI treatment (median survival 42.5 days with RRV-RLI vs. 20.0 days with intratumoral PBS treatment in NK-depleted mice, $p<0.001$, Log-Rank Mantel-Cox, **Figure 5I and J**) and was not statistically significantly different than RLI-RRV + Isotype control ($p>0.05$, Log-Rank Mantel-Cox, **Supplementary Figure 3E**). However, combined CD4 and CD8 depletion abrogated RLI-RRV survival and CD8 depletion alone did not **Figure 5B-E**. This suggests that, while NK cells may contribute to the survival benefit seen with RRV-RLI treatment, NK cells are not necessary for this survival benefit. Instead, $CD8^+$ and $CD4^+$ T cells coordinate to serve as the primary co-drivers of RRV-RLI therapeutic efficacy, with both together needed for this survival benefit in a manner that the therapeutic benefit can continue as long as one of these T-cell subtypes is present, but is lost once both are gone, a versatility that has implications for the translatability of this approach where patients could derive a benefit unless they had a dramatic reduction in levels of both T-cell subtypes. We have added the NK cell depletion to the results section (Page 7, Lines 249-253) and have added additional points around this topic to the discussion (Page 12, Lines 483-487).

Comment #8: Supplementary Fig. 5B,C: Use of Tox as the only gene indicating CD8 or NKT exhaustion is not sufficient.

Response #8: We thank the reviewer for this comment. In **Supplementary Figure 5B and C**, our intent was not to comment on levels of T cell exhaustion between lymphocytes from RRV-RLI+TMZ vs. RRV-RLI groups, but to highlight that, while there weren't many gene expression changes in the lymphocytes from these two groups, among the most significant changes were that adding TMZ to RRV-RLI led to increased lymphocyte granzyme expression and decreased lymphocyte *Tox* expression, both of which would be consistent with a more activated T-cel phenotype as granzymes are associated with increased activation of NK cells and $CD8^+$ T-cells, while *Tox* is an HMG-box transcription factor that serves as a central regulator that can transcriptionally and epigenetically program CD8+ T-cell exhaustion such that *Tox* downregulation as seen with addition of TMZ to RRV-RLI is associated with increased T-cell activation.[13,14] We have amended the discussion to clarify that we are describing overall transcriptional tendencies rather than classifying cell states (Page 12, Lines 510-512).

Comment #9: The results showing that increased osteopontin (Spp1) from myeloid/macrophages may enhance immune activation in RRV-RL1 +/-TMZ groups (Fig. 7G and Supplementary Fig.

5G) are contradictory to the report that myeloid-derived Spp1 drives CD8+ T-cell exhaustion (PMID: 36638785).

Response #9: We thank the reviewer for this important point on the mixed literature around SPP1 (osteopontin). We agree that SPP1 has been shown to be involved in some immunosuppressive mechanisms, especially in binding CD44. At the same time, loss of SPP1 has been shown to be pro-tumorigenic and SPP1 is a well-described marker of GBM-associated macrophages in general.[15] In our dataset, we also see inferred binding of SPP1 to T cell integrins (ITGA4_ITGB1 and ITGAV_ITGB1), which may also reflect an association with tissue remodeling and immune migration.[16] The context-dependent nature of SPP1 expression is also supported by the co-occurrence of increased antigen presentation pathways in some of the cell types with SPP1 expression. We appreciate the reviewer in bringing up this point and have highlighted the nuance around SPP1 by amending the text to mention that SPP1 is a glioma-associated macrophage marker that may be representative of counter-regulatory immunosuppressive or immune migratory pathways, and citing the corresponding literature (Page 10, Lines 373-375).

Comment #10: Increased cross-presentation to CD8+ T-cells by myeloid cells in RRV-RL1 +/- TMZ groups is inferred from the CellChat analysis. A direct experiment proving this is missing.

Response #10: We thank the reviewer for this comment. Our conclusion of increased cross-presentation in the RRV-RLI +/- TMZ groups is based on the CellChat interactions, the broad increase in MHC Class I protein expression in myeloid populations (**Figure 7F**), and the increased number of recurrent T cell receptors in the RRV-RLI + TMZ group as compared to RRV-RLI + Vehicle. Interestingly, this pattern is consistent with prior TMZ literature, which demonstrates that TMZ can improve cross-presentation as well as augment tumor-specific T-cell responses in patients and preclinical models.[17–19] In addition, IL-15 is well known to support antigen presentation pathways and antigen presenting cell function.[20–22]

We agree with the reviewer that a direct experiment was needed to support the increase in antigen presence after treatment with TMZ. To further clarify the role of TMZ in releasing tumor antigens that lead to enhanced antigen presentation, we have performed a functional cross-presentation assay. In this experiment, we utilize SB28 tumor cells expressing ovalbumin (OVA) and assess the ability of dendritic cells pulsed with their supernatant to activate OT-1 CD8 T cells (which recognize OVA SIINFEKL peptide presented by the MHC class I). We compare OT-1 activation between SB28-OVA cells that were treated with TMZ and those without TMZ treatment. In SB28- OVA cells treated with TMZ, we see an increase in downstream OT-1 T cell activation (as evidenced by $CD69^+$, 13.8% vs. 8.8%, $p<0.05$, Welch's t-test, **Supplementary Figure 6A**) and cell proliferation (as evidenced by $Ki67^+$, 11.7% vs. 7.2%, $p<0.05$, Welch's t-test, **Supplementary Figure 6B**). These data further support a model where TMZ cell death increases antigen supply, which then synergizes with RRV-RLI enhancement of T cell, NK cell, and antigen presentation responses. We have amended the results section (Page 10, Line 376-378), **Supplementary Figure 6A-B,** and the discussion (Page 13, Line 527-529) to reflect these findings.

Comment #11: The tumor-reactivity of T-cells and the lack of immunogenicity of the RRV backbone are inferred by TCR analysis. A direct experimental strategy proving the tumor-reactivity without the RRV reactivity of expanded T-cells in RRV-RL1 +/-TMZ groups is required.

Response #11: We thank the reviewer for their astute comment. Given the extremely low T cell infiltration in our SB28 model (<5% in PBS or RRV-treated mice) and the technical challenge of tumor-infiltrating lymphocyte isolation, we cannot reliably recover sufficient T cells for *ex vivo* antigen-specific assays. As such, we infer the tumor-specificity rather than RRV-specificity of the

immune response after RRV-RLI treatment through multiple convergent datasets. First, as detailed in **Response 4**, using co-culture experiments (**Figure 2G-I, Supplementary Figure 1D-H**), *in vivo* transcriptomic (**Supplementary Figure 2E-H**), *in vivo* flow cytometric (**Figure 6I-N**), and survival data (**Figure 3B-E**) we do not observe a significant difference between PBS and RRV treatment groups, whereas RRV-RLI demonstrated differences from both. This data from our work and previous work[4] suggest that the RRV backbone is relatively immunologically inert and isolating the biological changes seen to the RLI payload. Second, the cross-presentation experiment detailed in **Response 9** demonstrates an increase in OT-1 T cell activation and proliferation after TMZ treatment that is specific to a tumor-antigen (in this case OVA), supporting the ability for TMZ treatment to amplify a tumor antigen-specific response. This is further supported by our single-cell RNA sequencing data, which demonstrates a broad increase in antigen presentation machinery and recurrent TCRs in the RRV-RLI + TMZ condition. Third, our single-cell beta chain TCR analysis not only demonstrates no matches to the envelope protein of MLV, but none of the TCRs reactive to the envelope protein of MLV were within 100 distance units for paired chains or 18 distance units for beta chains in our samples (Page 10, Lines 399-405). Only 2.3% (42/1813) of beta chain TCRs were within 18 distance units of a pathogen-specific TCRs from McPAS-TCR. In the revised manuscript, we also performed an additional paired alpha-beta chain analysis. In this analysis, we have identified 1/1364 exact alpha-beta matches to a pathogen in McPAS (influenza) and 35/1364 alpha-beta pairs within a 100-unit distance of McPAS pathogen antigens. We have added these new findings to the results section (Page 10, Lines 411-412). Finally, our Tu2449 rechallenge data, where immunologic memory was observed against untreated tumors in the Tu2449 model, also supports a tumor specific response.

Comment #12: Supplementary 1F: It is not clear if NK cells or CD8+ T-cells are analyzed here based on the legend.

Response #12: We thank the reviewer for pointing this out. There was a typographical error in the original figure legend that has now been corrected. **Supplementary Figure 1F** (now **Supplementary Figure 1E** after revisions) is in reference to $CD8^+$ T cells, and we have addended the figure legend to reflect this.

<u>Reviewer #3's Comments:</u>

The authors have a strong track record in clinical trials of replicating retroviral vectors (RRV) for glioblastoma and have continued to develop these vectors for more effective patient treatment. Despite promising results, Phase III trials revealed that the suicide gene (CD) was insufficient to kill glioma cells and stimulate long-term anti-tumor immunity. In this manuscript, the authors evaluate a new RRV armed with cDNA encoding a superagonist form of IL-15 (RLI), a cytokine that stimulates NK and $CD8^+$ T cells. Using murine glioma cell lines SB28 and Tu2449, they provide evidence of the efficacy of this new virus, RRV-RLI, including profiling immune cell populations and TCR repertoires using the 10x platform.

Comment #1: There are some concerns in experimental setting; the lack of a control virus in TIL analysis, particularly in Figures 4 and 7, limits the ability to determine the IL-15 specific effects.

Response #1: We thank the reviewer for this insightful comment. Regarding the lack of control RRV virus for TIL analysis, we have completed additional *in vivo* experiments where we directly compare immune infiltration in mice treated with RRV vs. RRV-RLI at a matched timepoint via flow cytometry. This flow cytometric analysis demonstrates significantly higher infiltration of $CD3^+$ T cells, $CD8^+$ T cells, NKT cells, and NK cells in RRV-RLI treatment groups relative to both PBS and RRV treatment groups, with no significant difference between PBS and RRV treatments

(**Figure 6I-M**). Similarly, RRV-RLI results in a proportional decrease in infiltrating CD11b$^+$ cells relative to RRV and PBS treatments with no difference between RRV and PBS groups (**Figure 6N**). We have amended the results section (Page 8, Line 293-311) and **Figure 6** to reflect these findings.

Regarding empty RRV as a control in addition to intratumoral PBS in other experiments (e.g. depletion studies, combination with TMZ, combination with anti-PD1 treatment, use of lower virus dose, and delayed viral injection) that came up as a comment from multiple reviewers, we discussed the use of PBS as a control group for these studies with the editor who, noting the lack of survival benefit with RRV treatment, suggested that further immune profiling of RRV tumors, which was the focus of this particular comment, would be more informative than use of RRV as a control group in these other studies. Prior work[4] and our own data demonstrate that RRV infection alone does not significantly impact tumor growth, survival, or immune infiltration compared to PBS controls, and both differ significantly from RRV-RLI. We do not observe any survival benefit associated with RRV treatment (**Figure 3B-E**). Transcriptomically, we show: (1) a figure demonstrating differences in immunologic gene pathways after RRV-RLI vs. RRV treatment at timepoint (14 days after tumor implantation, 10 days after viral implantation) (**Supplementary Figure 2E)**; (2) a volcano plot demonstrating differential gene expression at timepoint after RRV-RLI vs. RRV treatment (**Supplementary Figure 2F)**: (3) a heat map demonstrating differences in gene expression after RRV-RLI vs. RRV treatment at timepoint (**Supplementary Figure 2G)**; and (4) transcriptomic immune-infiltration comparison as a heat map (**Supplementary Figure 2H)** comparing tumors receiving RRV at a timepoint to tumors receiving RRV-RLI at timepoint and at endpoint when the tumor has progressed to become fatal after initial response, which revealed reversion of the immune microenvironment to an immunologically "cold" state with low T and NK cell infiltration in the setting of progression after initially successful RRV-RLI treatment, similar to the response to RRV. In addition, co-culture experiments with both T and NK cells show no increase in activation or proliferation with RRV or untreated tumor cells (**Figure 2G-I, Supplementary Figure 1D-H**), but show a large increase in activation and proliferation with RRV-RLI treated cells, isolating the biological changes seen to the RLI payload. Furthermore, as eventual treatment in patients would be RRV-RLI rather than RLI alone, we highlighted the comparison between PBS and RRV-RLI as we believe this to be the most translationally relevant comparison. In addition, in our single-cell TCR sequencing data we do not see TCRs that are targeted against the MLV envelope and only 2.3% of TCRs were within 18 distance units of a pathogen-specific TCR (**Figure 7H** and Page 10, Lines 399-412). These data support that the immune modulation and therapeutic benefit of RRV-RLI is due to the RLI payload rather than the RRV vector backbone. We have added additional reasoning around this topic of RRV as a control to the discussion section (Page 12, Lines 475-480).

Comment #2: Additionally, in vivo treatment starting at day 4 post-tumor may coincide with scar healing from needle injection, raising questions about whether RRV-RLI infects parenchymal and innate cells for persistent IL-15 production rather than targeting the tumor itself. This stage mainly recruits NK cells, making RRV-RLI more effective at stimulating NK cells while adaptive immune cells typically start infiltrating at day 6-7 in this model. This timing may eliminate seed tumors before generating a tumor microenvironment. Discussing these points would be appreciated, considering RRV's naturally non-lytic feature.

Response #2: We thank the reviewer for their comment. To address this concern, we have performed additional mouse studies. First, just as the reviewer suggested, we completed another survival experiment with RRV-RLI, but included a group that was dosed at day 7 post-tumor implantation. We found that administration of RRV-RLI at day 7 still prolonged survival compared to PBS control (median survival 17 days vs. 21 days, $p<0.05$). While there was a trend towards reduced survival in the RRV-RLI day 7 injection as compared to RRV-RLI day 4, the difference

did not quite reach significance (median survival 21 days vs. 50.5 days, p=0.064, **Supplementary Figure 3J**). This appeared consistent with a kinetic constraint such that delaying therapy provided less time for vector spread and RLI accumulation in the fast-growing SB28 model (control average median survival ~17 days).

In addition, we completed a slightly different experiment along this same theme of creating a more biomimetic scenario of a less favorable ratio of viral particles to tumor cells at the time of implantation, when we reduced the viral dose 10-fold ($2.5x10^4$ TU, **Supplementary Figure 3K**), leading to maintenance of therapeutic efficacy relative to PBS control (median survival 18 days vs. 31 days, p<0.01), albeit to a lesser degree than the efficacy seen at the 10-fold higher dose (median survival 18 days vs. 56 days, p<0.001), again suggesting a dose/kinetic impact on therapeutic efficacy. Finally, we don't believe we are eliminating seed tumors that haven't fully established as, on bioluminescent imaging, RRV-RLI treated tumors frequently grow similarly to control until approximately 10-14 days after implantation (**Figure 3B-E**). We have added these findings to the results section (Page 8, Lines 273-279) as well as additional information around this topic in our discussion (Page 12, Line 496-498)

We also appreciate the reviewer's points regarding off-target infectivity and early NK cell activation as a primary driver of therapeutic efficacy. We believe that several lines of evidence make this less likely. First, RRV is a γ-retrovirus that requires actively dividing cells and mitosis in order to infect, stably integrate its payload, and spread due to the lack of nuclear localization sequence in the RRV genome. This makes normal brain parenchyma or scar tissue after tumor implantaton a less favorable target given its largely post-mitotic nature.[23] To further address this topic and confirm that RRV-RLI specifically replicated in dividing cells which are limited to the tumor in the brain, we have assessed brain parenchyma contralateral to RRV-RLI injected tumors at tumor endpoint. Even with high viral copies/ng of DNA in the tumor, we do not see significant evidence of viral infectivity in contralateral brain tissue, suggesting that the virus is unable to replicate and spread effectively in normal brain parenchyma (**Supplementary Figure 2D**). Second, we have performed an NK cell depletion experiment which did not abrogate the therapeutic response to RRV-RLI. This finding, combined with the complete abrogation in combined CD4 and CD8 depletion, suggests that the primary driver of the therapeutic benefit of RRV-RLI is the adaptive immune system (**Figure 5**).

Comment #3 (three points related to Figure 1):

- For Figure 1 analysis, the manuscript is missing details on patient samples, data, analysis methods, and the IRB statement.
- Was tumor size considered when comparing IL-15 high vs. IL-15 low GBM tumors, especially since IL-15 high tumors have fewer tumor cells?
- Were the resected tumor samples taken from the tumor core or edges? The sampling location can affect the analysis of tumor-infiltrating cells.

Response #3: **Figure 1** analyses were performed on external, publicly available tumor datasets.[24,25] No new human samples were collected for our study, eliminating the need for IRB approval for our work. Information regarding specifics around data analysis methods can also be found in the methods section under the headers: *Human bulk RNA-sequencing analysis* and *Human scRNA and immune cell profiling analysis.* We do not have access to the spatial distribution of collected samples for bulk RNA-sequencing. For the scRNA sequencing data, cells were taken from the core and edge. There was a comparable percent of core cells in the IL-15 high and low groups (63.6% vs. 66.6%, p=0.66, Page 4, Lines 100-106), and for the purposes of our analyses, we did not distinguish between core vs. edge. There is no available information on tumor size for

these data sets. We have added additional clarification around these points to the methods section (Page 17-18, Line 766, 793-795).

Comment #4: The authors demonstrated that co-culturing T- and NK cells with RRV-RLI promotes their expansion and leads to their activation, as indicated by increased CD69 and Granzyme B expression. Given that RRV is known to infect immune cells and use them as carriers, culturing T cells with the virus in the absence of tumor cells could help determine whether the observed effects are due to direct viral influence on T cells rather than IL-15 secreted by infected tumor cells (i.e, direct viral infection to T cells in co-culture and its impact on T cell function and proliferation)

Response #4: We thank the reviewer for this helpful comment. To directly address the reviewer's point about direct viral infection of T cells in co-culture, we have added RRV-RLI directly to isolated T cells without tumor cells. In this treatment group (**Figure 2G, Supplementary Figure 1D**), we do not see a significant change in activation or proliferation marker expression between T cells incubated with RRV-RLI, T cells alone, T cells exposed to RRV-infected tumor cells, or T cells exposed to uninfected tumor cells. In contrast, T cells exposed to RRV-RLI infected tumors demonstrated significantly higher activation and proliferation, isolating T cell changes to the RLI payload. We believe this indicates that the activation and proliferation of immune cells seen in the co-culture experiments is primarily related to RLI secretion by infected tumor cells. We have added these findings to the results section (Page 5, Lines 161-162). Also, of relevance to this point, we should also mention that, in experiments assessing the activation of T cells and NK cells in response to RRV-RLI, the immune cells were co-cultured with tumor cells that were either uninfected, infected by RRV (empty virus), or infected by RRV-RLI. Given the replicating nature of RRVs, tumor cells infected with RRV or RRV-RLI are continuously producing their respective virus (in addition to RLI, in the case of RRV-RLI infected tumors). As such, T. cells or NK cells in the RRV co-culture setting are exposed to circulating RRV viral particles in the cell media. The lack of increased immune cell activation or proliferation in the RRV alone group relative to PBS suggests that possible RRV infectivity of T-cells or NK-cells is unlikely to have contributed to changes in immune cell function and proliferation seen during co-culture of these immune cells with RRV-RLI-infected tumor. Cells.

Comment #5: Considering that tumor-infiltrating myeloid cells or microglia may infiltrate in response to the virus and potentially limit viral replication, this could reduce IL-15 secretion and contribute to T cell exhaustion near the study endpoint. How long does IL-15 agonist secretion from viruses persist in vivo? It is suggested to analyze native IL-15 gene and RLI transcripts obtained from RNA-seq data (e.g., Fig 4) or through IHC and RT-PCR at analysis points.

Response #5: We thank the reviewer for this helpful comment. As suggested, we have completed an additional *in vivo* mouse study where we assessed viral genome presence, RLI transgene presence, and RLI protein expression in a timepoint experiment (14 days after tumor implantation, 10 days after viral delivery), which is consistent with our timepoints used for tumor microenvironment analysis. At this timepoint, RRV-RLI-infected tumors exhibited robust viral genome presence (mean viral genome/ng gDNA 13.25 RRV-RLI vs. 0 in PBS control, **Supplementary Figure 2A**) and RLI protein expression (mean RLI/IL-15 pg/mL 381 in RRV-RLI vs. 0 in PBS control **Supplementary Figure 2B**). We have added these findings to the results section (Page 6, Lines 179-183).

Comment #6: In Figure 4D, the heatmap shows increased expression of immune checkpoint inhibitors like CTLA-4, PD-1, and TIGIT in the treatment group, and Figure 4F shows an increased immune score for exhausted CD8 T cells. Results and discussion lack this data interpretation.

Could the T cells be exhausted due to an immunosuppressive environment promoted by the virus or tumor factors? Did the authors assess if immunosuppressive cells like TAMs or MDSCs increased with virus infection? Were other cytokines like IFN-γ or IL-10 assessed in the tumor microenvironment or in vitro, as they might contribute to T cell exhaustion? Probably, it would be appreciated to add more discussion.

Response #6: We thank the reviewer for their comment. Regarding the higher "exhausted" CD8 score in the RRV-RLI treated group as compared to the PBS treatment groups, we believe this due to multiple factors. First, there is extremely low T cell infiltration in PBS and RRV treated SB28 mice, as can be seen in our flow cytometric analysis (**Figure 6**, <5% infiltration for $CD3^+$, $CD4^+$, and $CD8^+$ T cell populations in PBS and RRV treated SB28 tumors). This is consistent with the literature for SB28[7,12] and part of the reason that SB28 is seen as a very immunologically "cold" model that recapitulates human GBM. As such, the increase in "exhausted" CD8 score on transcriptomic analysis is likely reflective of an absolute change in the number of CD8 T cells rather than an increase in the relative ratio of exhausted and non-exhausted CD8 T cell populations. Indeed, when we assess the ratio of exhausted to non-exhausted CD8 T cells, the ratio in the RRV-RLI treatment is more favorable than that observed in the control group (1.2 vs. 0.84, p=0.04, Student's t-test, Page 7, Lines 217-219). Second, a higher exhausted CD8 score in the RRV-RLI treatment group is in many ways expected with antigen-drive activation. In antigen driven activation, activated $CD8^+$ T cells can upregulate markers such as PD1, which are markers of both activation and exhaustion.[3] The upregulation of markers like PD1 may contribute to a higher "exhausted" $CD8^+$ T cell score, but in fact be representative of activation. This is supported by the 5' single cell RNA sequencing data (**Figure 7C**), which examines PBS and RRV-RLI treated mice. In the scRNA sequencing data we observe a small population of "exhausted" $CD8^+$ T cells, but much larger populations of $CD8^+$ effector cells, early activated T cells, and NKT cells. We also observed a decrease in MDSC-like neutrophil populations (**Supplementary Fig. 5F,** Page 9, Lines 358-361). To investigate T-cell exhaustion further, we performed additional culture experiments directly evaluating the impact of RLI exposure on T cell exhaustion markers. In these experiments, we observe an increase in the activation marker CD69 alongside expression of PD-1, which in this context is likely an activation marker rather than an exhaustion marker as it occurred without significant concordant single or multi-expression of other exhaustion markers (TIM3, LAG3, CTLA4). We have amended the results to reflect these findings. (**Supplementary Fig. 1I**, Page 5, Lines 156-162).

Comment #7: RRV-RLI monotherapy significantly improved survival in the Tu2449 GBM model, with 90% of mice showing long-term survival and developing immunologic memory. However, this effect was not observed in the SB28 model. What factors or aspects of the tumor microenvironment contribute to the different outcomes in SB28 and Tu2449? Could this difference be due to variations in infectivity or replication of the virus in these models?

Response #7: We thank the reviewer for their comment and astute points. We believe the primary driver of differences in tumor response and survival are related to the underlying immunogenicity of each model. While Tu2449 is also poorly immunogenic relative to most murine GBM models and has been used frequently in the literature, it does exhibit higher T-cell infiltration compared to the SB28 model, indicating increased relative immunogenicity. SB28 is one of the least immunogenic models available and is characterized by low T cell infiltration and a low tumor mutational burden, similar to human GBM.[7,12] While differences in viral replication and spread are certainly possible, we think this is less likely, as viral replication was similar between the two models during head-to-head comparisons in culture (**Figure 2C and D**). We have clarify this topic in our discussion section (Page 11, Lines 457-462)

Attachment - revised version of previous point-by-point rebuttal with corrected line numbers

Comment #8: The TCR analysis concludes that the identified TCRs represent a tumor-specific response enhanced by RRV-RLI and RRV-RLI+TMZ rather than an antiviral response to RRV-RLI. However, this statement needs supportive evidence and might be better placed in the discussion section. Using scRNA with TCR profiling to identify matching alpha-beta chains at the single-cell level would support this claim by addressing specific antigen-responsive T cells with reconstituted TCR analysis.

Response #8: We thank the reviewer for this comment. In the results section of the original manuscript, we focused on the beta-chain TCR repertoire from our 5' single cell RNA sequencing experiment. This showed minimal overlap with pathogen-reactive TCRs in McPAS. In response to the reviewer's astute comment, we have also performed a paired alpha-beta analysis for the revised manuscript. In this analysis, we have identified 1/1364 exact alpha-beta matches to a pathogen in McPAS (influenza) and 35/1364 alpha-beta pairs within a 100-unit distance of McPAS pathogen antigens. We have added this to the text (Page 10, Lines 411-412). In addition, as discussed in **Responses 1 and 4,** using multiple orthogonal modalities, we do not observe a significant immune reaction to RRV alone. In addition, the immunologic memory observed to untreated tumors cells in the Tu2449 rechallenge experiment suggests a tumor specific immune response, rather than an anti-viral immune reponse. Altogether, we believe that the greater clonal expansion with RRV-RLI + TMZ as well as low beta-only and alpha-beta chain pathogen overlap is most representative of an anti-tumor immune response that is augmented by TMZ in the setting of RRV-RLI. While the strength of our conclusion that TCR profiling suggested a tumor-specific rather than viral-specific immune response was enhanced by following the reviewer's astute suggestion to add alpha-beta analysis, we also appreciate the reviewer's need to temper some of these conclusions and have therefore presented the new alpha-beta analysis in the results section of the revised manuscript (Page 10, Lines 411-412), while also moving our rationale and statement around the tumor-specific response to the discussion as suggested by the reviewer (Page 12, Lines 514-519).

Comment #9 - Minor Concerns:
In general, where scRNA-seq data is presented, the font size of text and axis labels appears too small, making it difficult to read. Below are specific instances where this issue needs attention:

- Figure 1A-C, 2B, 6D-G, 6H, 7: The font size of the axis labels, annotation and text is too small, making it difficult to read.
- Figure 4: The axis labels and text throughout the figure—except for Figure 4F—are not legible and should be adjusted.
- Figure 2B: The axes of the flow cytometry data are not labeled. It would be beneficial to include axis titles instead of relying solely on color coding.
- Line 185: 'Figure 3B' should be corrected to 'Figure 4B.'

These revisions will enhance readability and clarity across the figures.

Response #9: We thank the reviewer for their comment. We have made all the suggested changes to improve the readability and clarity of the figures. We appreciate the reviewer's insight.

In summary, we believe these revisions significantly solidify the mechanistic foundation and translational applicability of RRV-RLI therapy. As we plan to take this novel treatment to patients, we believe these additional studies provide a more rigorous assessment of the therapy and its potential for clinical translation. We thank the reviewers again for their constructive comments.

Sincerely,

Attachment - revised version of previous point-by-point rebuttal with corrected line numbers

Manish K. Aghi, MD PhD
Professor and Vice Chair of Neurosurgery, UCSF
Neuro-Oncology Program Leader, UCSF Cancer Center
Faculty, UCSF Graduate Division in Biomedical Sciences (BMS)

Attachment - revised version of previous point-by-point rebuttal with corrected line numbers

References for Response to Reviewers:

1. Saikh KU, Khan AS, Kissner T, Ulrich RG. IL-15-induced conversion of monocytes to mature dendritic cells. *Clin Exp Immunol*. 2001;126(3):447. doi:10.1046/J.1365-2249.2001.01672.X
2. Zannikou M, Duffy JT, Levine RN, et al. IL15 modification enables CAR T cells to act as a dual targeting agent against tumor cells and myeloid-derived suppressor cells in GBM. *J Immunother Cancer*. 2023;11(2):6239. doi:10.1136/JITC-2022-006239
3. Simon S, Labarriere N. PD-1 expression on tumor-specific T cells: Friend or foe for immunotherapy? *Oncoimmunology*. 2017;7(1):e1364828. doi:10.1080/2162402X.2017.1364828
4. Hiraoka K, Inagaki A, Kato Y, et al. Retroviral replicating vector-mediated gene therapy achieves long-term control of tumor recurrence and leads to durable anticancer immunity. *Neuro Oncol*. 2017;19(7):918-929. doi:10.1093/neuonc/nox038
5. Gubin MM, Zhang X, Schuster H, et al. Checkpoint Blockade Cancer Immunotherapy Targets Tumour-Specific Mutant Antigens. *Nature*. 2014;515(7528):577. doi:10.1038/NATURE13988
6. Mathios D, Park CK, Marcus WD, et al. Therapeutic administration of IL-15 superagonist complex ALT-803 leads to long-term survival and durable antitumor immune response in a murine glioblastoma model. *Int J Cancer*. 2016;138(1):187-194. doi:10.1002/ijc.29686
7. Genoud V, Marinari E, Nikolaev SI, et al. Responsiveness to anti-PD-1 and anti-CTLA-4 immune checkpoint blockade in SB28 and GL261 mouse glioma models. *Oncoimmunology*. 2018;7(12):e1501137. doi:10.1080/2162402X.2018.1501137
8. Chu TH, Vo MC, Lakshmi TJ, et al. Novel IL-15 dendritic cells have a potent immunomodulatory effect in immunotherapy of multiple myeloma. *Transl Oncol*. 2022;20:101413. doi:10.1016/J.TRANON.2022.101413
9. Ohteki T, Suzue K, Maki C, Ota T, Koyasu S. Critical role of IL-15-IL-15R for antigen-presenting cell functions in the innate immune response. *Nat Immunol*. 2001;2(12):1138-1143. doi:10.1038/NI729
10. Huang Q, Liu Y, Du Y, Garmire LX. Evaluation of Cell Type Annotation R Packages on Single-cell RNA-seq Data. *Genomics Proteomics Bioinformatics*. 2021;19(2):267-281. doi:10.1016/J.GPB.2020.07.004
11. Desbois M, Desbois M, Desbois M, et al. IL-15 superagonist RLI has potent immunostimulatory properties on NK cells: Implications for antimetastatic treatment. *J Immunother Cancer*. 2020;8(1):632. doi:10.1136/jitc-2020-000632
12. Haddad AF, Young JS, Amara D, et al. Mouse models of glioblastoma for the evaluation of novel therapeutic strategies. *Neurooncol Adv*. 2021;3(1):1-16. doi:10.1093/NOAJNL/VDAB100
13. Khan O, Giles JR, McDonald S, et al. TOX transcriptionally and epigenetically programs CD8+ T cell exhaustion. *Nature*. 2019;571(7764):211. doi:10.1038/S41586-019-1325-X
14. Wang X, He Q, Shen H, et al. TOX promotes the exhaustion of antitumor CD8+ T cells by preventing PD1 degradation in hepatocellular carcinoma. *J Hepatol*. 2019;71(4):731-741. doi:10.1016/J.JHEP.2019.05.015
15. Szulzewsky F, Schwendinger N, Güneykaya D, et al. Loss of host-derived osteopontin creates a glioblastoma-promoting microenvironment. *Neuro Oncol*. 2017;20(3):355. doi:10.1093/NEUONC/NOX165
16. Inoue M, Shinohara ML. Role of osteopontin and integrin alpha-v in T cell-mediated anti-inflammatory responses in endotoxemia. *J Immunol*. 2015;194(12):5595. doi:10.4049/JIMMUNOL.1500623

17. Kim TG, Kim CH, Park JS, et al. Immunological factors relating to the antitumor effect of temozolomide chemoimmunotherapy in a murine glioma model. *Clin Vaccine Immunol*. 2010;17(1):143-153. doi:10.1128/CVI.00292-09
18. Sanchez-Perez LA, Choi BD, Archer GE, et al. Myeloablative temozolomide enhances $CD8^+$ T-cell responses to vaccine and is required for efficacy against brain tumors in mice. *PLoS One*. 2013;8(3). doi:10.1371/JOURNAL.PONE.0059082
19. Sampson JH, Aldape KD, Archer GE, et al. Greater chemotherapy-induced lymphopenia enhances tumor-specific immune responses that eliminate EGFRvIII-expressing tumor cells in patients with glioblastoma. *Neuro Oncol*. 2011;13(3):324-333. doi:10.1093/NEUONC/NOQ157
20. Brandt K, Bulfone-Paus S, Foster DC, Rückert R. Interleukin-21 inhibits dendritic cell activation and maturation. *Blood*. 2003;102(12):4090-4098. doi:10.1182/BLOOD-2003-03-0669
21. Anguille S, Smits ELJM, Cools N, Goossens H, Berneman ZN, Van Tendeloo VFI. Short-term cultured, interleukin-15 differentiated dendritic cells have potent immunostimulatory properties. *J Transl Med*. 2009;7. doi:10.1186/1479-5876-7-109
22. Chu TH, Vo MC, Lakshmi TJ, et al. Novel IL-15 dendritic cells have a potent immunomodulatory effect in immunotherapy of multiple myeloma. *Transl Oncol*. 2022;20:101413. doi:10.1016/J.TRANON.2022.101413
23. Collins SA, Shah AH, Ostertag D, Kasahara N, Jolly DJ. Clinical development of retroviral replicating vector Toca 511 for gene therapy of cancer. *Expert Opin Biol Ther*. 2021;21(9):1199-1214. doi:10.1080/14712598.2021.1902982
24. Hoadley KA, Yau C, Hinoue T, et al. Cell-of-Origin Patterns Dominate the Molecular Classification of 10,000 Tumors from 33 Types of Cancer. *Cell*. 2018;173(2):291-304.e6. doi:10.1016/J.CELL.2018.03.022
25. Darmanis S, Sloan SA, Croote D, et al. Single-Cell RNA-Seq Analysis of Infiltrating Neoplastic Cells at the Migrating Front of Human Glioblastoma. *Cell Rep*. 2017;21(5):1399-1410. doi:10.1016/J.CELREP.2017.10.030
